# Supplementary material for: Dinitroimidazoles as bifunctional bioconjugation reagents for protein functionalization and peptide macrocyclization
Source: Nat Commun. 2019 Jan 11;10:142. doi: 10.1038/s41467-018-08010-2 (PMC6329768; doi:10.1038/s41467-018-08010-2)
Supplement: Supplementary file 1 — Supplementary Information [file 41467_2018_8010_MOESM1_ESM.pdf]

Supplementary Information

Dinitroimidazoles as Bifunctional Bioconjugation Reagents for Protein Functionalization and Peptide Macrocyclization

By Luo et al

Supplementary Methods

Chemicals and instrumentation

Solvents were obtained from Sigma-Aldrich, Alfa-Aesar and Acros and used directly without further purification unless indicated. Amino acids and peptides were obtained from commercial sources or prepared by solid phase peptide synthesis. Bovine serum albumin was purchased from BBI Life Sciences and used without further purification. Sortase A 5M (SrtA 5M) was a kind gift from Prof. Yi Cao at Nanjing University. Analytical thin layer chromatography was performed on 0.25 mm silica gel 60-F254. Visualization was carried out with UV light. ^{1}H NMR spectra were recorded on Bruker AMX-400 instrument (400 MHz) or Bruker DRX-600 instrument (600 MHz). The following abbreviations (or combinations thereof) were used to explain multiplicities: s = singlet, d = doublet, t = triplet, q = quartet, m = multiplet, br = broad. Coupling constants, J, were reported in Hertz unit (Hz). ^{13}C NMR spectra were recorded on Bruker AMX-400 instrument (100 MHz) or Bruker DRX-600 instrument (150 MHz), and were fully decoupled by broad band proton decoupling. High-resolution mass spectra (HRMS) were recorded on an Agilent Mass spectrometer using ESI-TOF (electrospray ionization-time of flight). HPLC profiles were obtained on Agilent 1260 HPLC system using commercially available columns.

Sequence of protein Sortase A 5M (SrtA)

SrtA is composed of 214 amino acids and contains 1 free Cysteine (C192) and 26 Lysines. Calculated M.W.(average) 24136.03 Da.

GSSHHHHHHSSGLVPRGSHMASMTGGQQMGRGSKPHIDNYLHDKDKDEKIE
QYDKNVKEQASKDKKQQAKPQIPKDKSKVAGYIEIPDADIKEPVYPGPATRE
QLNRGVSFAEENESLDDQNISIAGHTFIDRPNYQFTNLKAAKKGSMVYFKVG
NETRKYKMTSIRNVKPTAVGVLDEQKGKDKQLTLITC^{192}DDYNEETGVWETR
KIFVATEVK*

Sequence of bovine serum albumin (BSA)

BSA is composed of 583 amino acids and contains one free cysteine Cys34 (35 Cys residues in total, and 34 cysteine residues are in the form of disulfide bonds) and 59 lysines. Calculated M.W.(average): 66429.98 Da

DTHKSEIAHRFKDLGEEHFKGLVLIAFSQYLQQC^{34}PFDEHVKLVNELTEFAKT
CVADESHAGCEKSLHTLFGDELCKVASLRETYGDMADCCEKQEPERNECFLS
HKDDSPDLPKLKPDPNTLCDEFKADEKKFWGKYLYEIARRHPYFYAPELLYY
ANKYNGVFQECCQAEDKGACLLPKIETMREKVLTSSARQRLRCASIQKFGER
ALKAWSVARLSQKFPKAEFVEVTKLVTDLTKVHKECCHGDLLECADDRADL
AKYICDNQDTISSKLKECCDKPLLEKSHCIAEVEKDAIPENLPPLTADFAEDKD
VCKNYQEAKDAFLGSFLYEYSRRHPEYAVSVLLRLAKEYEATLEECCAKDDP
HACYSTVFDKLKHLVDEPQNLIKQNCDQFEKLGEYGFQNALIVRYTRKVPQV
STPTLVEVSRSLGKVGTRCCTKPESERMPCTEDYLSLILNRLCVLHEKTPVSEK
VTKCCTESLVNRRPCFSALTPDETYVPKAFDEKLFTFHADICTLPDTEKQIKKQ
TALVELLKHKPKATEEQLKTVMENFVAFVDKCCAADDKEACFAVEGPKLVV
STQTALA*

Methods for HPLC analysis

Analytical HPLC analysis was performed using Phenomenex C18 (5 μm, 2.0 × 150 mm) analytical columns with mobile phase of water-acetonitrile-(0.1% formic acid) at a flow rate of 1.0 mL/min. Gradient used： isocratic 2% CH_3CN for 5 min, then 2% to 90% CH_3CN in 25 min, then 90% CH_3CN for 5 min, then isocratic 90% to 2% CH_3CN in 5 min. For quantification, phenol was added as an internal standard in all assays.

Modification of peptides and proteins by 1, 4-dinitroimidazoles

Typically, peptide (0.1 mM) was incubated with 1, 4-dinitroimidazole (0.1 mM) and in HEPES buffer (100 mM) at indicated pH at 25 °C for indicated time before analyzed by HPLC and MS.

For protein modification, 20 μM protein was incubated with 10 eq. of 1,4-dinitroimidazole derivative in PBS buffer at pH 7.0 at 25 °C for 1 h. The reaction mixture was quenched by addition of 1% HCOOH or removing the excess compound by PD10 desalting column before subjected to LC-MS analysis.

Fluorescent labelling of BSA via CuAAC reaction

BSA-**4c** conjugate was prepared by incubating 15 μM BSA and 5 eq. compound **4c** in 100 mM PBS buffer at pH 7.0. The excess compound **4c** was then removed by PD10 desalting column, and the BSA-**4c** conjugate was eluted with 100 mM PBS buffer, pH 7.2. BSA-**4c** conjugate (15 μM) was further incubated with 100 μM sulfo-Cy3-azide, 250 μM $CuSO_4$, 500 μM BTTAA, and 2.5 mM freshly prepared sodium ascorbate for 1 h at 25 ℃ before quenching with 5 mM BCA. The reaction samples were analyzed by SDS-PAGE gels and imaged by Gel Doc^{TM} XR+ (Bio-Rad). The protein gels were also stained by Coomassie Brilliant Blue (CBB).

Circular dichroism analysis

Procedure: peptide **P8** (0.6 mg) and peptide **P8c** (0.9 mg) was dissolved in CH_3CN (HPLC grade) and Mill-Q water (v/v = 1/1) to give 1.0 mM solution, respectively. CD instrument: Jasco (J-810). Scan wavelength: 180-800 nm. Cuvette: 0.5 mm. The experiment was conducted at 25 ºC.

Macrocyclization of lysine peptides with bis(1,4-DNIm) agent 5a

Procedure for lysine peptide macrocyclization: To a round-bottom flask warmed at 37 ºC was added DMSO (415 μL), 5 μL of 0.4 M solution of DIEA (final concentration 4.0 mM) in DMSO and 50 μL of 10 mM solution of peptide (final concentration 1.0 mM) in Milli-Q water. The mixture was stirred for several minutes before 30 μL of 20.0 mM solution of bis(1,4-DNIm) **5a** (final concentration 1.2 mM) in CH3CN was added. After 2 hours of stirring, the solution was neutralized with diluted formic acid and

subjected to analysis with LC-MS. Conversion is determined by using the peak AUC of the linear peptide at monitor wavelength.

Macrocyclization of peptides P1-P4 with compound 5a

General procedure: To a round-bottom flask was added successively HEPES buffer (pH 7.0, 100 mM), peptide substrates (1.0 mM) in Milli-Q water, sodium ascorbate (2.0 mM) and compound **5a** (1.0 mM). The mixture was stirred at room temperature for 10 min before acidified with diluted formic acid and subjected to analysis with LC-MS. Conversion is determined by using the peak AUC of the linear peptide at monitor wavelength.

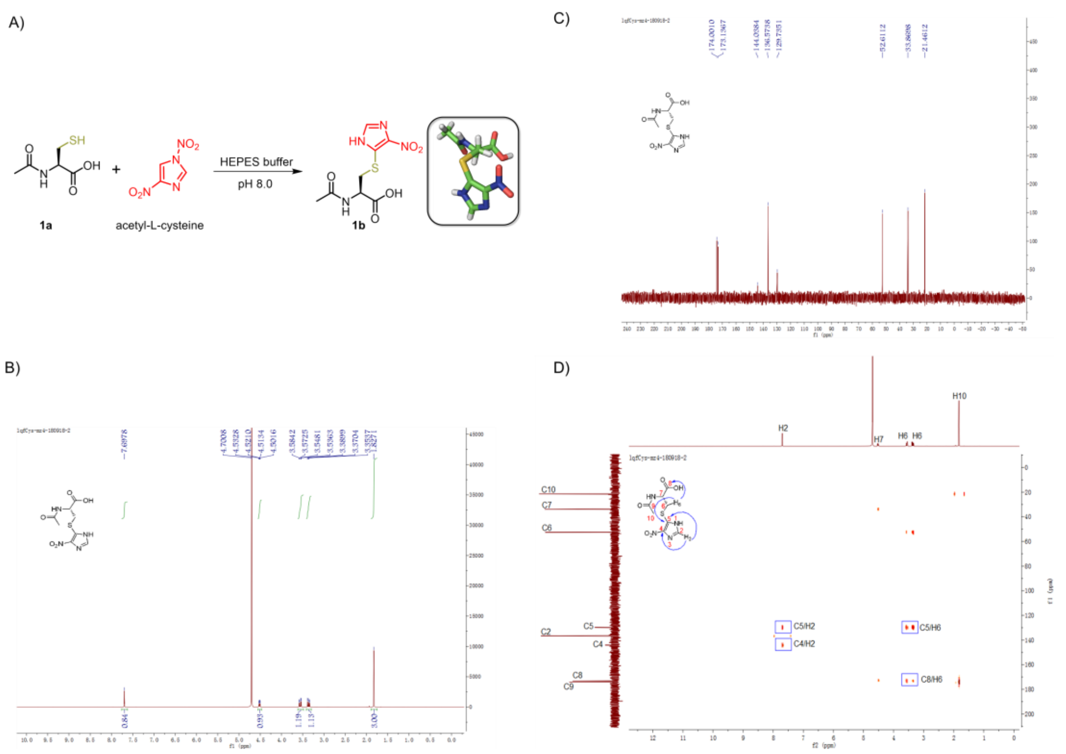

Supplementary Figure 1. Structural characterization of compound **1b**. A). Reaction scheme and crystal structure of compound **1b**; B). ^{1}H NMR spectrum of compound **1b**; C) ^{13}C NMR spectrum of compound **1b**; D) HMBC spectrum of compound **1b**. Key correlations are highlighted.

^{1}H NMR (400 MHz, D_2O) δ 7.79 (s, 1H), 4.61 (dd, *J* = 7.8, 4.7 Hz, 1H), 3.65 (dd, *J* = 14.5, 4.7 Hz, 1H), 3.45 (dd, *J* = 14.5, 7.8 Hz, 1H), 1.92 (s, 3H). ^{13}C NMR (100 MHz, D_2O) δ 174.00, 173.14, 144.04, 136.57, 129.74, 52.61, 33.87, 21.46

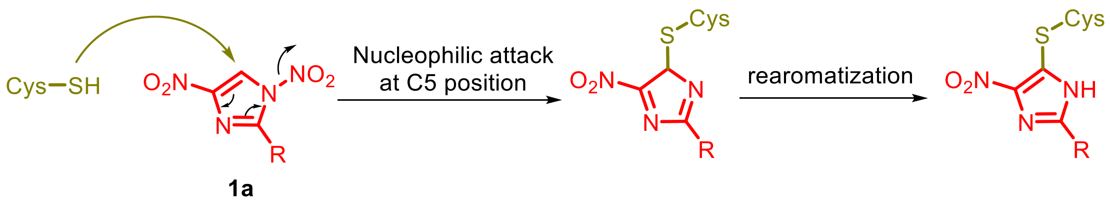

Supplementary Figure 2. Proposed reaction mechanism of compound **1a** reacting with cysteine.

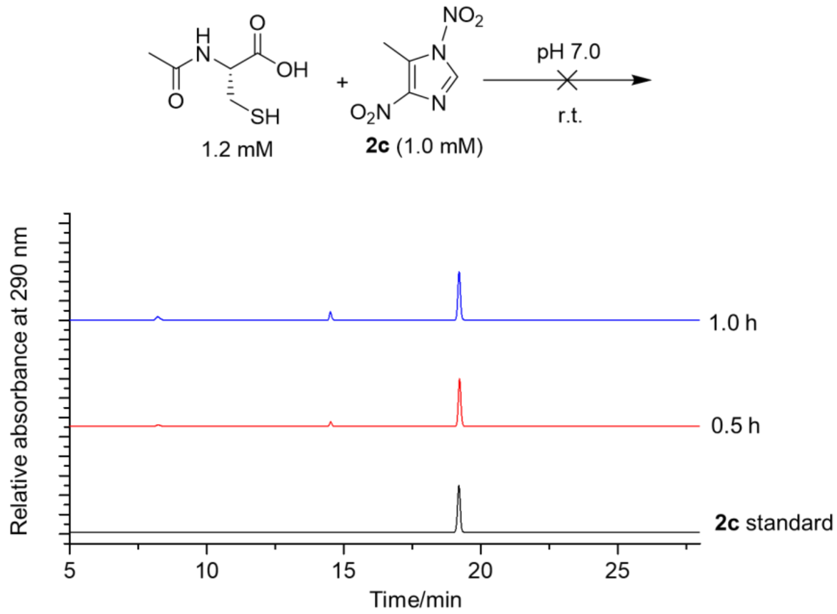

Supplementary Figure 3. HPLC analysis of the reaction between 5-methyl- 1,4-dinitroimidazole (**2c**) and cysteine thiol under neutral aqueous conditions.

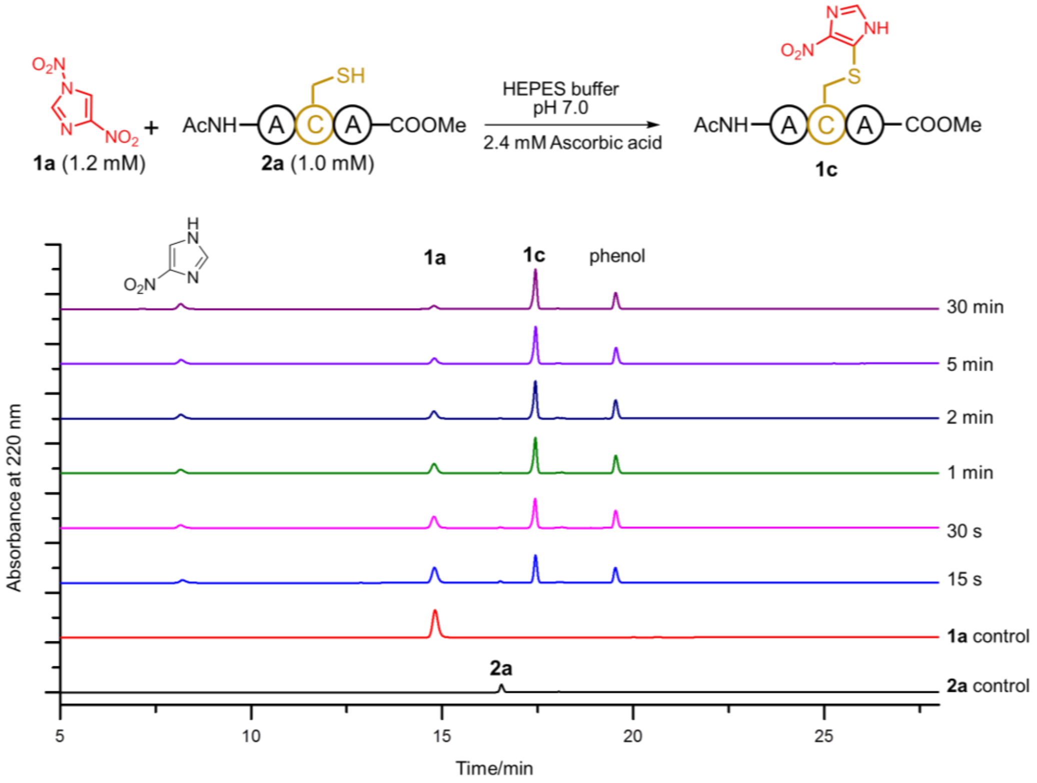

Supplementary Figure 4. HPLC analysis of reactions between compound **1a** (1.2 mM) and tripeptide **2a** (1.0 mM) under pH 7.0 for indicated time at 25 °C. Phenol was added as an internal standard for quantification. In the presence of tripeptide **2a**, a small portion of **1a** was converted into 4-nitroimidazole along the reaction process. Ascorbic acid (2.4 mM) was added to prevent the oxidative formation of disulfide bonds.

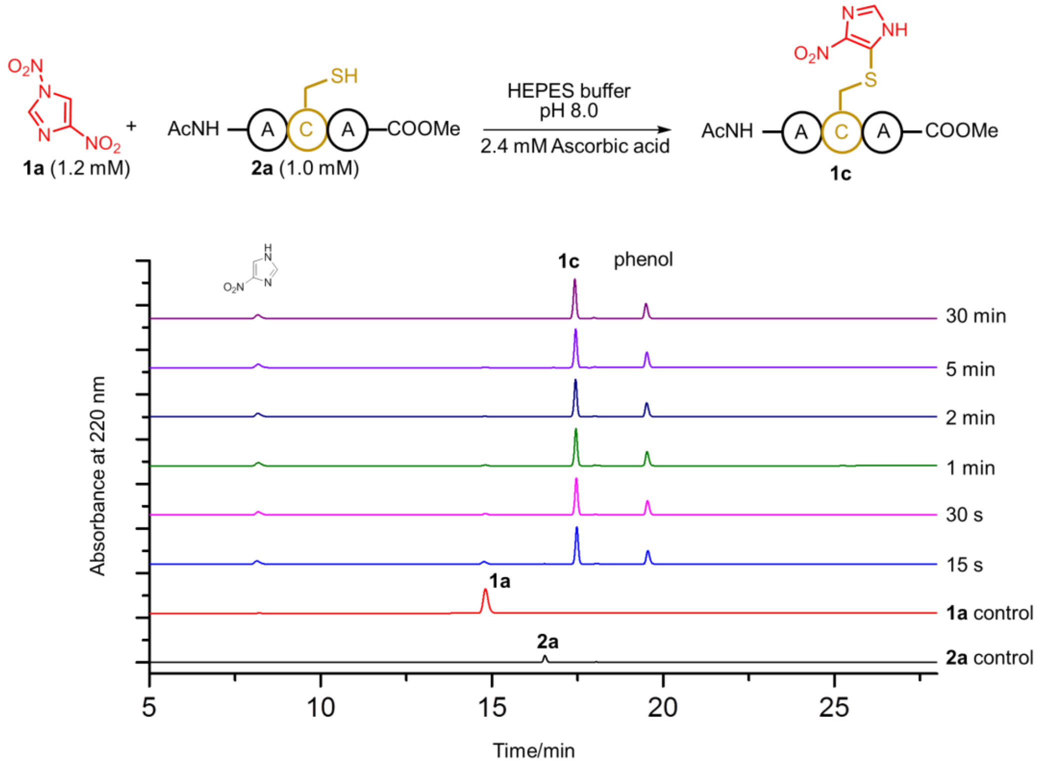

Supplementary Figure 5. HPLC analysis of reactions between compound **1a** (1.2 mM) and tripeptide **2a** (1.0 mM) under pH 8.0 for indicated time at 25 °C. Phenol was added as an internal standard for quantification. Ascorbic acid (2.4 mM) was added to prevent the oxidative formation of disulfide bonds.

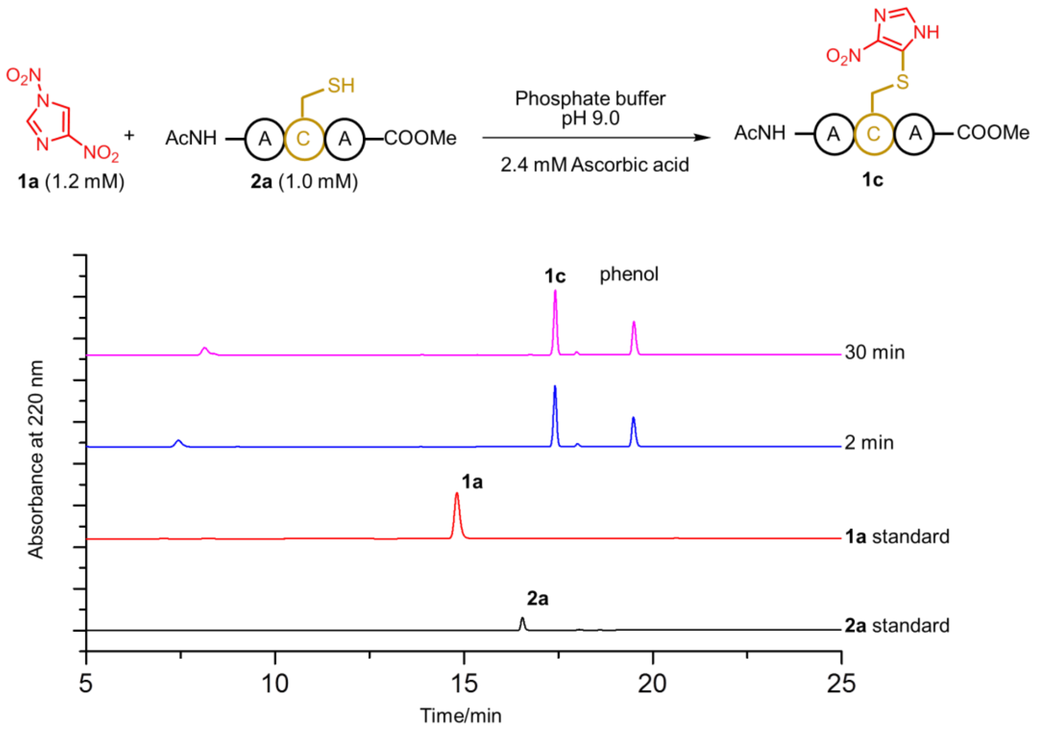

Supplementary Figure 6. HPLC analysis of reactions between compound **1a** (1.2 mM) and tripeptide **2a** (1.0 mM) under pH 9.0 for indicated time at 25 °C. Phenol was added as an internal standard for quantification. Ascorbic acid (2.4 mM) was added to prevent the oxidative formation of disulfide bonds.

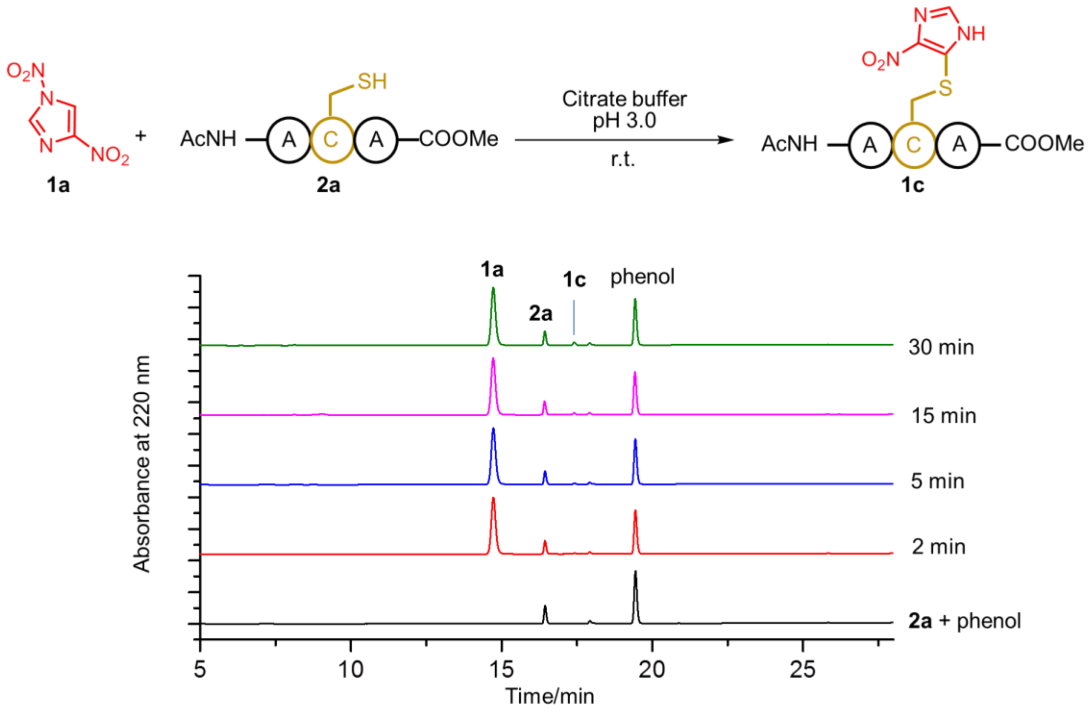

Supplementary Figure 7. HPLC analysis of reactions between compound **1a** (1.2 mM) and tripeptide **2a** (1.0 mM) under pH 3.0 at room temperature. Phenol was added as an internal standard for quantification. Ascorbic acid (2.4 mM) was added to prevent the oxidative formation of disulfide bonds.

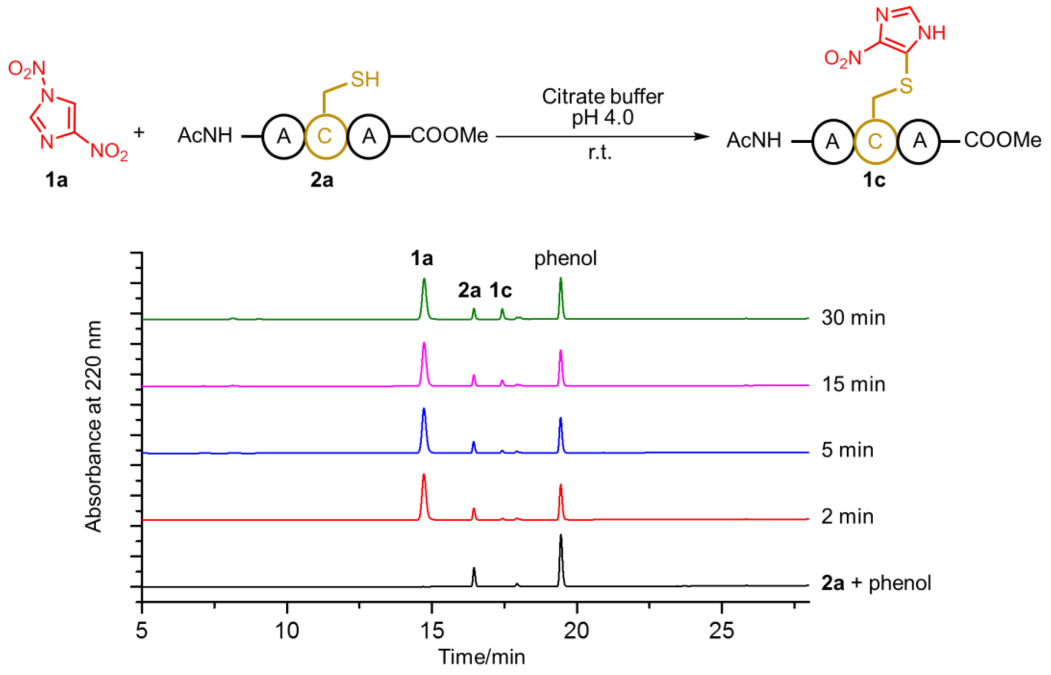

Supplementary Figure 8. HPLC analysis of reactions between compound **1a** (1.2 mM) and tripeptide **2a** (1.0 mM) under pH 4.0 at room temperature. Phenol was added as an internal standard for quantification. Ascorbic acid (2.4 mM) was added to prevent the oxidative formation of disulfide bonds.

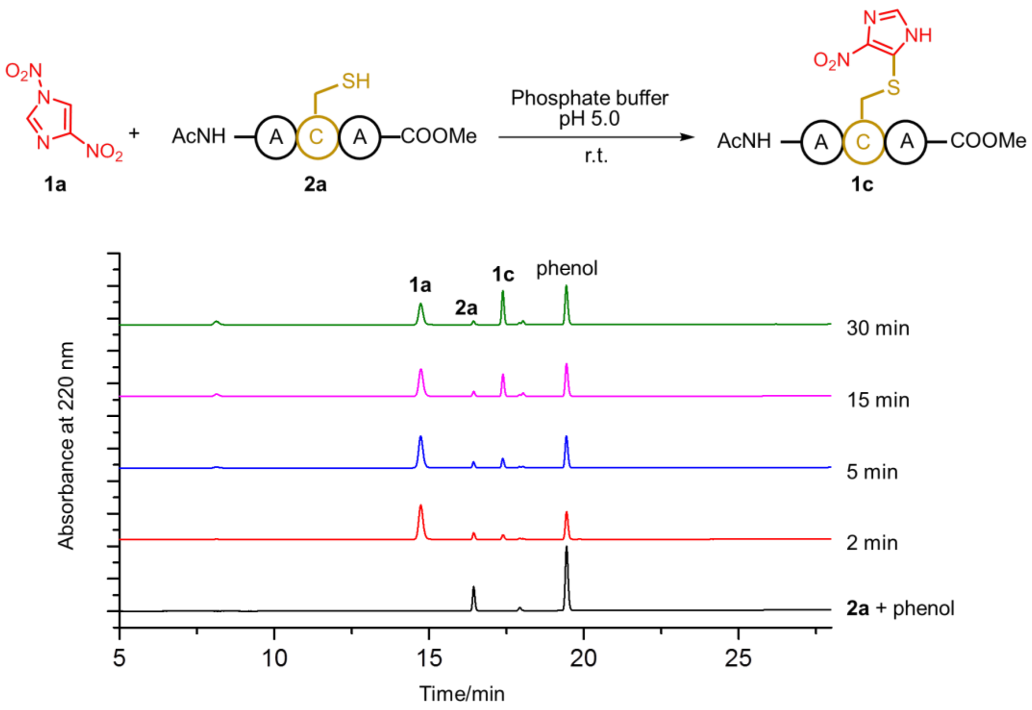

Supplementary Figure 9. HPLC analysis of reactions between compound **1a** (1.2 mM) and tripeptide **2a** (1.0 mM) under pH 5.0 at room temperature. Phenol was added as an internal standard for quantification. Ascorbic acid (2.4 mM) was added to prevent the oxidative formation of disulfide bonds.

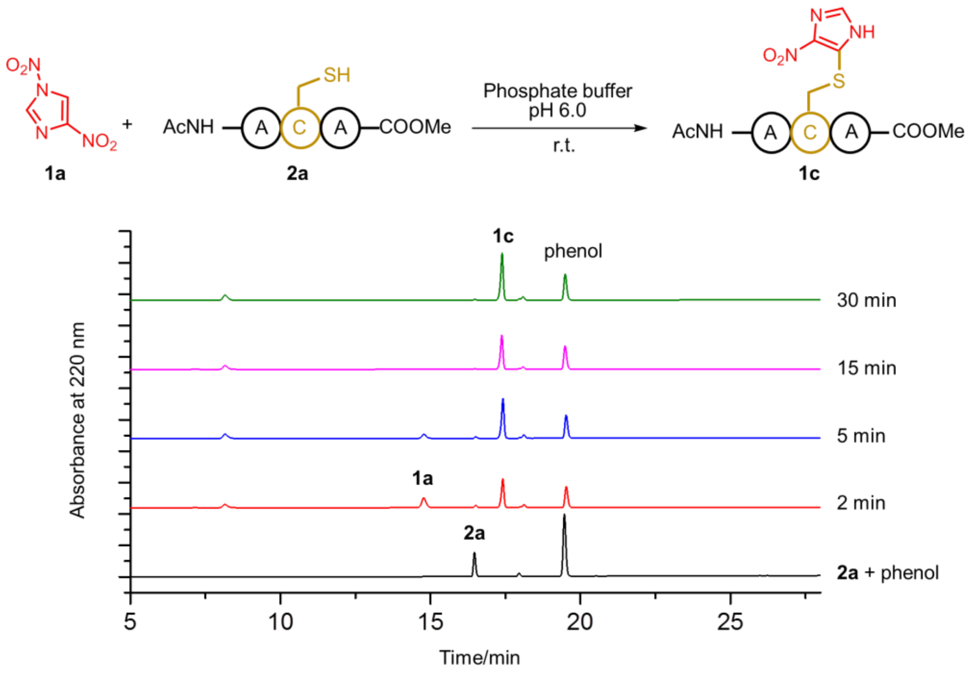

Supplementary Figure 10. HPLC analysis of reactions between compound **1a** (1.2 mM) and tripeptide **2a** (1.0 mM) under pH 6.0 at room temperature. Phenol was added as an internal standard for quantification. Ascorbic acid (2.4 mM) was added to prevent the oxidative formation of disulfide bonds.

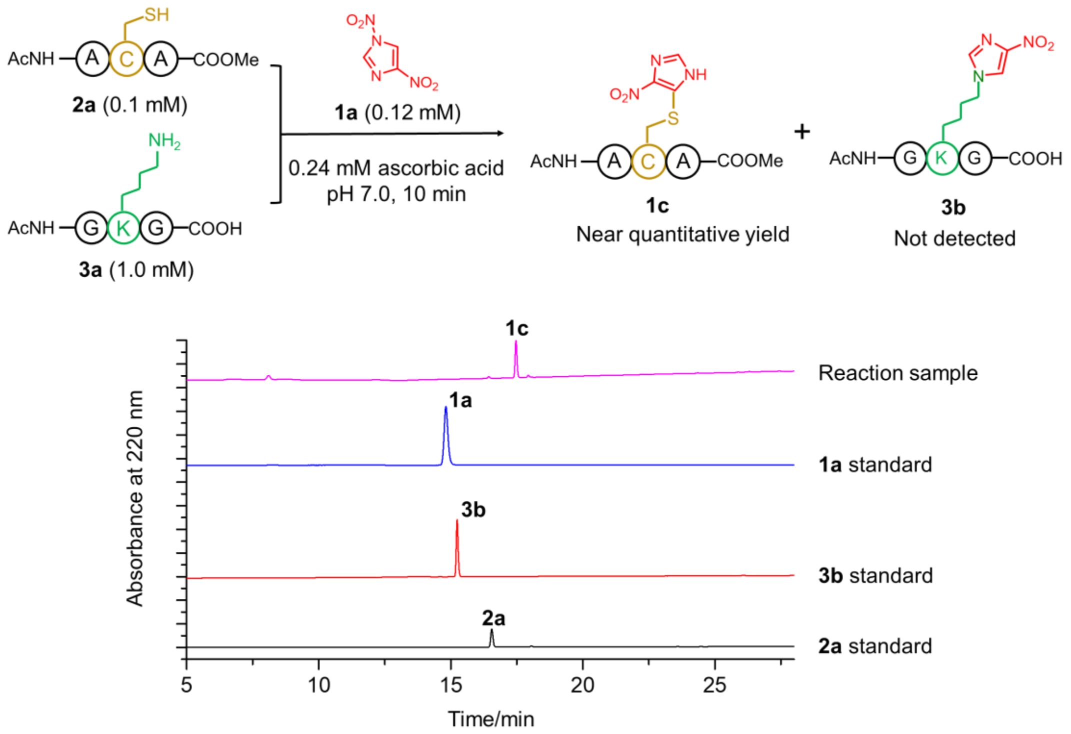

Supplementary Figure 11. Competitive reactions of compound **1a** (0.12 mM, 1.2 eq.) with tripeptides **2a** (0.1 mM, 1.0 eq.) and **3a** (1.0 mM, 10 eq.) under pH 7.0 for 10 min at 25 °C. As indicated above, near quantitative conversion of peptide **2a** were detected by HPLC. No product of peptide **3a** was observed. Peptide **3a** has a retention time less than 2 min and therefore is not shown.

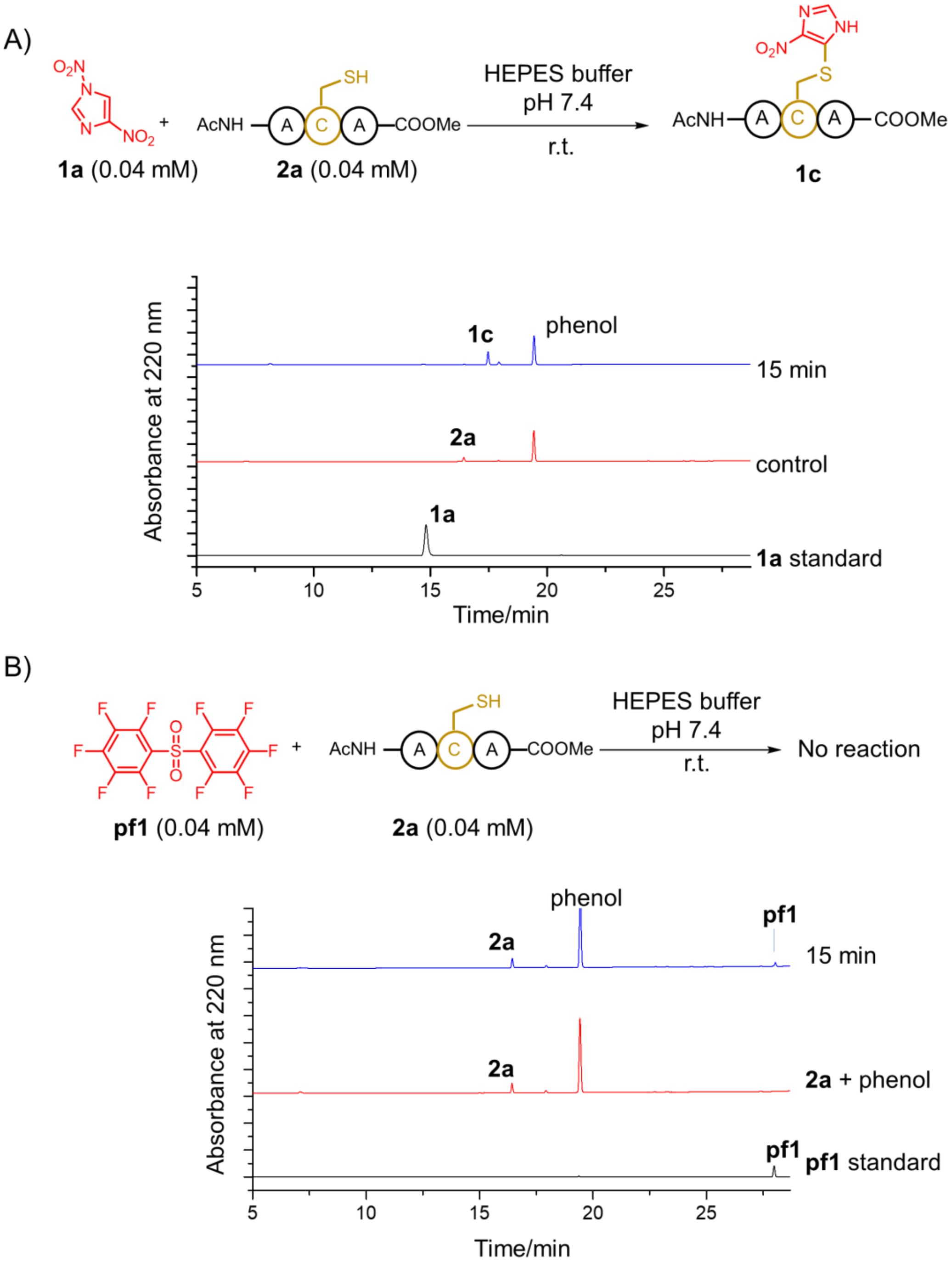

Supplementary Figure 12. Reactions between tripeptides **2a** and 1,4-DNIm **1a** or perfluoroaryl reagent **pf1**. A) 1,4-DNIm **1a** (0.04 mM) modified tripeptide **2a** near-quantitatively in 15 min. B) Perfluoroaryl reagent **pf1** (0.04 mM) was not able to modify tripeptide **2a** in 15 min.

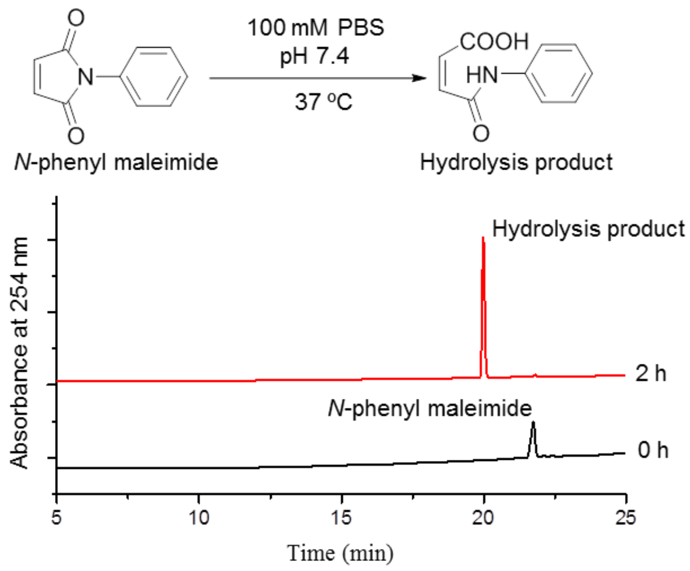

Supplementary Figure 13. Stability of *N*-phenyl maleimide in 100 mM PBS buffer, pH 7.4 at 37 °C for indicated time before subjected to HPLC analysis.

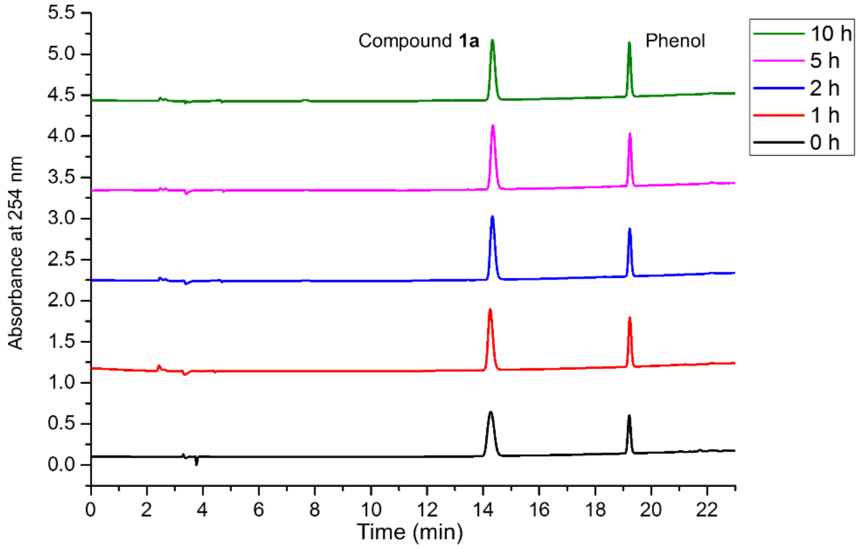

Supplementary Figure 14. Stability of compound **1a** in 100 mM PBS buffer, pH 7.4 at 37 °C for indicated time before subjected to HPLC analysis.

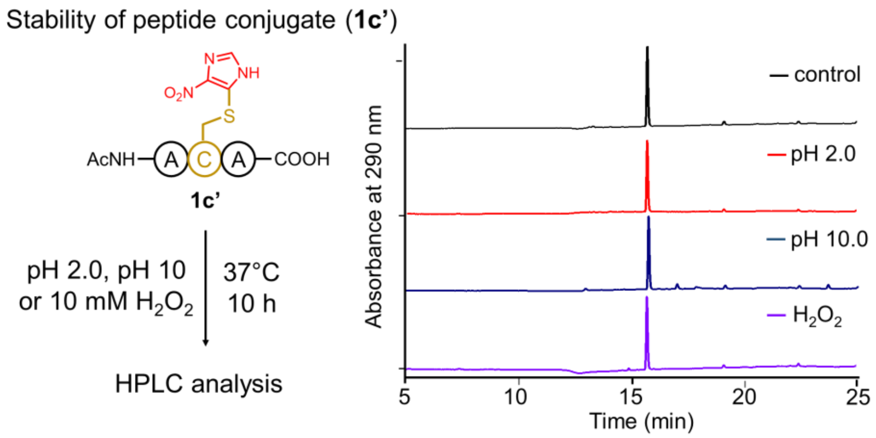

Supplementary Figure 15. Stability of 4-nitroimidazole-ACA adduct **1c'** (0.05 mM) in buffers at pH 2.0, pH 10.0, or in 10 mM H_2O_2 at 37 °C for 10 h before subjected to HPLC analysis. Gradient used: isocratic 2% CH_3CN for 5 min, then 2% to 90% CH_3CN in 25 min, then isocratic 90% CH_3CN for 5 min, then 90% to 2% CH_3CN in 5 min. Monitor wavelength: 365 nm.

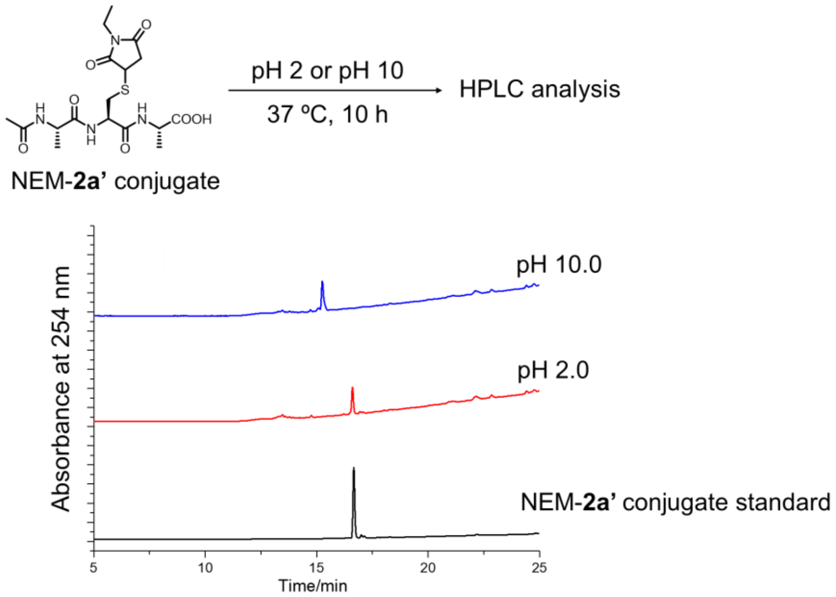

Supplementary Figure 16. Stability of NEM-**2a'** conjugate (0.05 mM) in buffers at pH 2.0 and 10.0. NEM-**2a'** conjugate was incubated at pH 2.0 and pH 10.0 at 37 °C for 10 h before subjected to HPLC analysis. Gradient used: isocratic 2% CH_3CN for 5 min, then 2% to 90% CH_3CN in 25 min, then isocratic 90% CH_3CN for 5 min, then 90% to 2% CH_3CN in 5 min. Monitor wavelength: 254 nm.

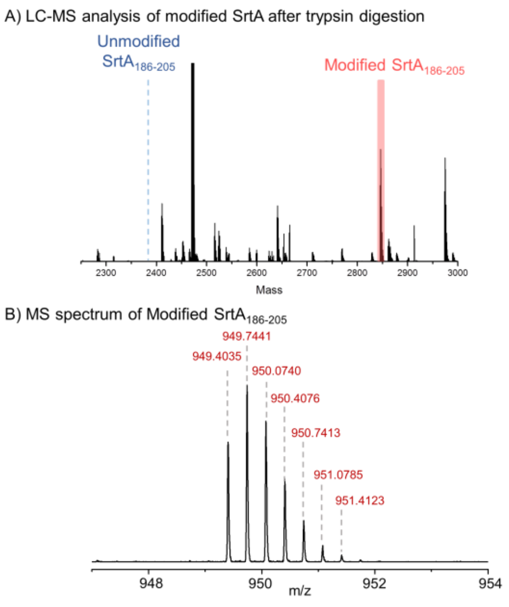

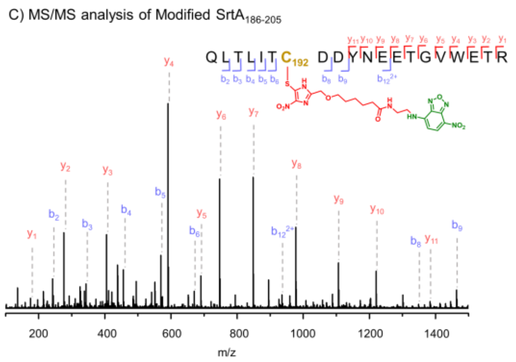

	theoretical m/z	observed m/z	relative deviation (ppm)
b2	242.1500	242.1512	4.9556
b3	343.1976	343.2004	8.1586
b4	456.2817	456.2810	-1.5341
b5	569.3658	569.3649	-1.5807
b6	670.4134	670.4114	-2.9832
b8	1348.5951	1348.5810	-10.4553
b9	1463.6220	1463.6231	0.7516
$b12^{2+}$	935.3891	935.3921	3.2072
y1	175.1190	175.1191	0.5710
y2	276.1667	276.1686	6.8799
y3	405.2093	405.2083	-2.4679
y4	591.2886	591.2869	-2.8751
y5	690.3570	690.3559	-1.5934
y6	747.3785	747.3764	-2.8098
y7	848.4261	848.4233	-3.3002
y8	977.4687	977.4687	0.0000
y9	1106.5113	1106.5053	-5.4224
y10	1220.5542	1220.5471	-5.8170
y11	1383.6176	1383.6119	-4.1196

Supplementary Figure 17. LC-MS/MS analysis of SrtA after modification by a 1,4-DNIm-NBD conjugate. A). LC-MS analysis of modified SrtA sample after trypsin digestion. $SrtA_{186-205}$ is the SrtA segment containing the Cys192 residue. The theoretical mass of unmodified $SrtA_{186-205}$ is 2385.0794 Da. The theoretical mass of modified $SrtA_{186-205}$ is 2845.2249 Da, which is observed at 2845.2020 Da. B) The zoomed-in MS spectrum of the modified $SrtA_{186-205}$ segment. C). MS/MS analysis of the modified $SrtA_{186-205}$ segment.

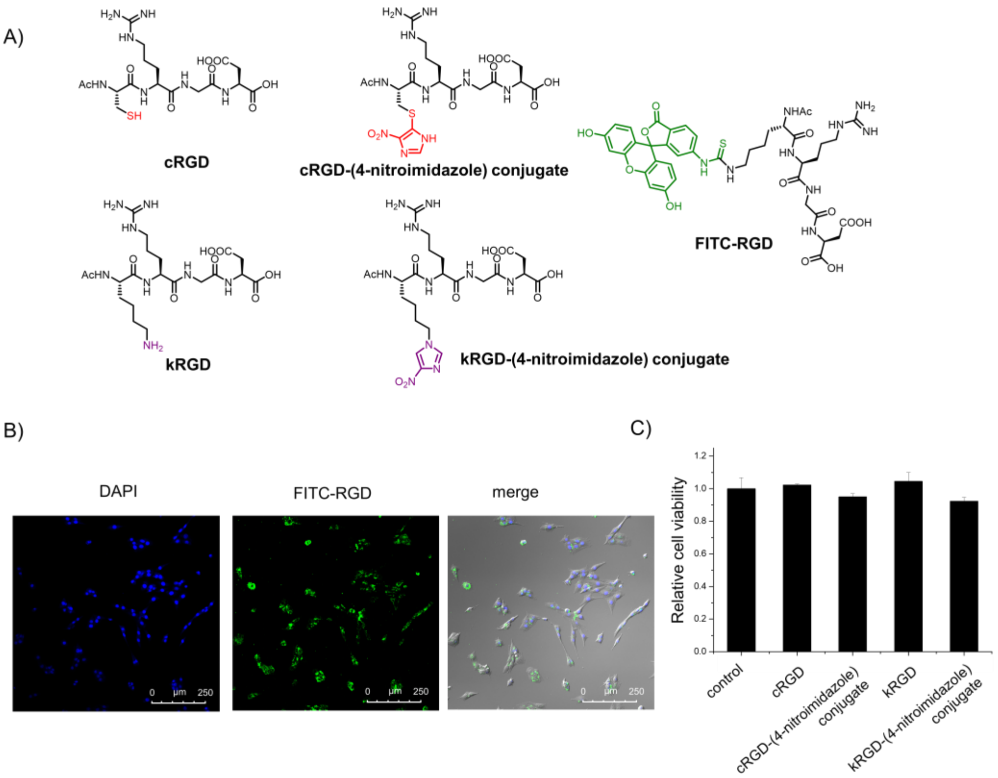

Supplementary Figure 18. A). Peptide derivatives synthesized for the evaluation of cellular toxicity. B). Cell staining assay by incubating FITC-RGD (10 μM) and 273T cells for 48 hours. C). Relative cell viability after treatment of each RGD peptide derivatives (10 μM), as determined by MTT method. The relative cell viability values represent means ±SDs (standard deviations) of three independent experiments. Peptide conjugates are characterized by HRMS: **cRGD**: HRMS (ESI) m/z calcd. for $C_{17}H_{30}N_7O_8S$ $[M+H]^+$ 492.1877, found 492.1887; **cRGD-(4-nitroimidazole) conjugate**: HRMS (ESI) m/z calcd. for $C_{20}H_{31}N_{10}O_{10}S$ $[M+H]^+$ 603.1945, found 603.1939; **kRGD**: HRMS (ESI) m/z calcd. for $C_{20}H_{36}N_8NaO_8$ $[M+Na]^+$ 539.2554, found 539.2556; **kRGD-(4-nitroimidazole) conjugate**: HRMS (ESI) m/z calcd. for $C_{23}H_{37}N_{10}O_{10}$ $[M+H]^+$ 613.2694, found 613.2715; **FITC-RGD**: HRMS (ESI) m/z calcd. for $C_{41}H_{48}N_9O_{13}S$ $[M+H]^+$ 906.3092, found 906.3089.

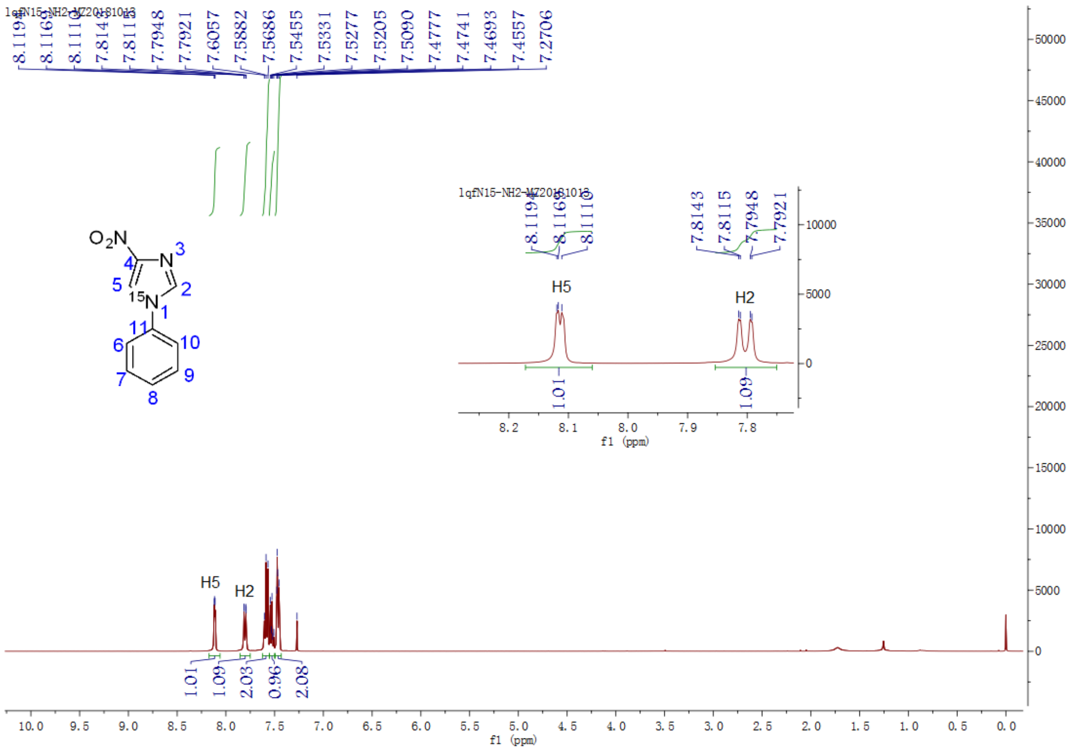

Supplementary Figure 19. ^{1}H NMR analysis of product **1e** generated from the reaction between ^{15}N-analine and (**1a**).

^{1}H NMR (400 MHz, $CDCl_3$) δ 8.11 (dd, $J_{H2\text{-}N1}$ = 3.4 Hz, $J_{H2\text{-}H5}$ =1.1 Hz, 1H), 7.80 (dd, $J_{H5\text{-}N1}$ = 7.8 Hz, $J_{H2\text{-}H5}$ = 1.1 Hz, 1H), 7.60-7.57 (m, 2H), 7.55–7.50 (m, 1H), 7.47 (m, 2H). H2 and H5 were coupled with ^{15}N nucleus. See ^{1}H NMR spectra of compound **1e'** in **Supplementary Figure 20** for comparison.

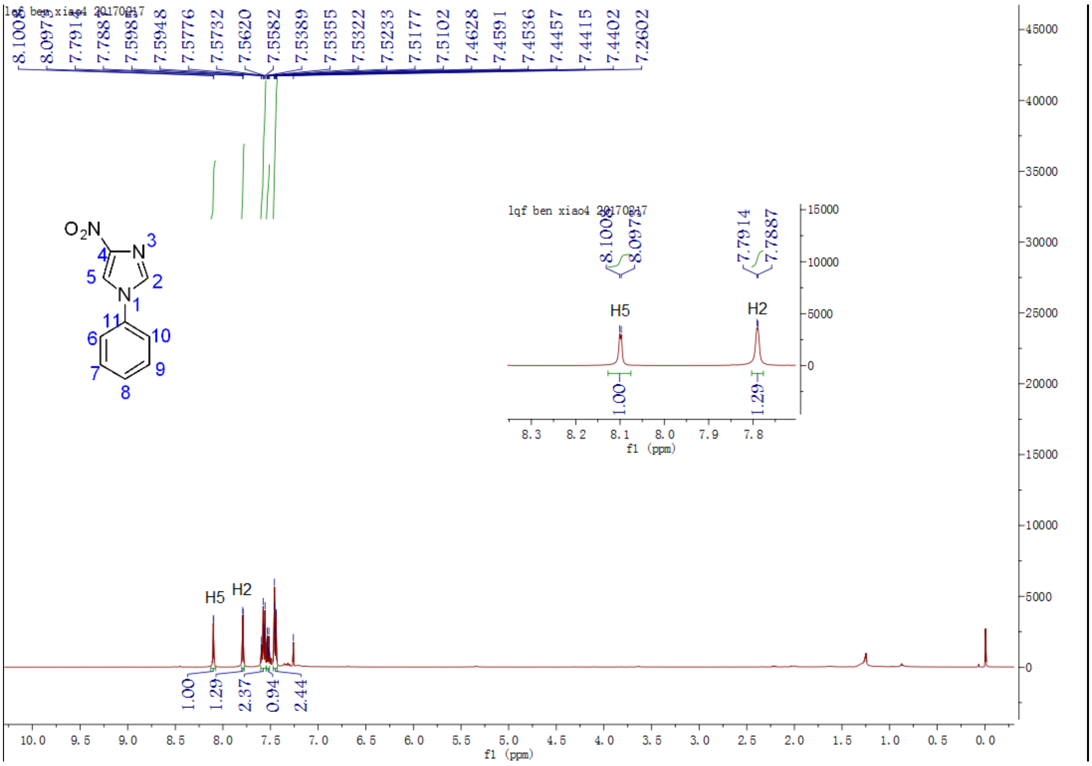

Supplementary Figure 20. ^{1}H NMR analysis of product **1e’** generated from the reaction between analine and (**1a**). ^{1}H NMR (400 MHz, $CDCl_3$) δ 8.10 (d, *J* = 1.4 Hz, 1H), 7.79 (d, *J* = 1.3 Hz, 1H), 7.58 (m, 2H), 7.53 (m, 1H), 7.45 (m, 2H).

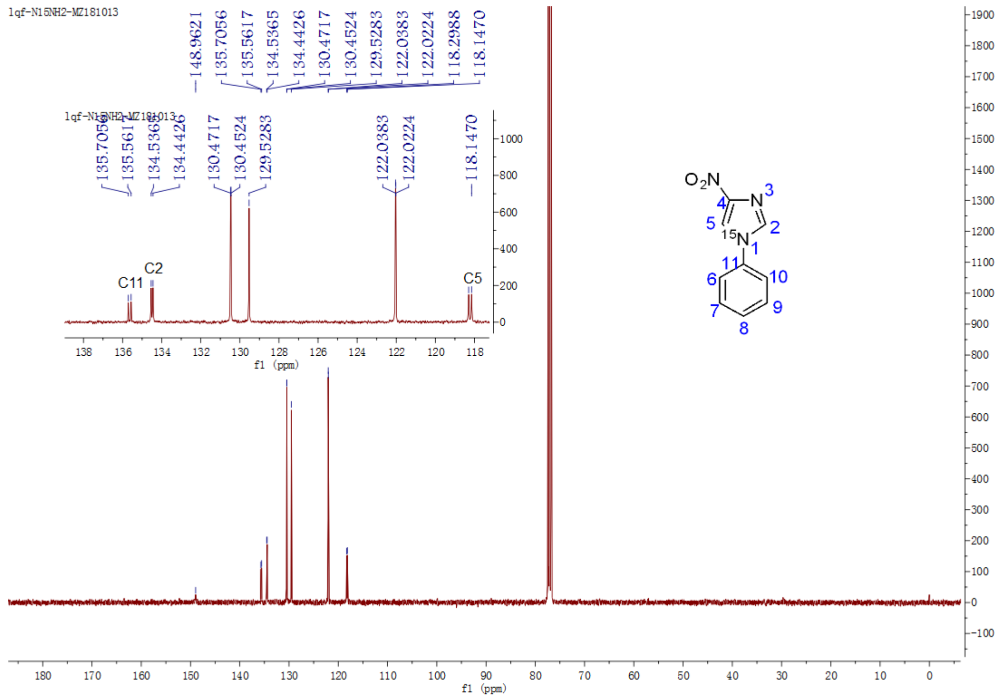

Supplementary Figure 21. ^{13}C NMR analysis of product **1e**. ^{13}C NMR (100 MHz, $CDCl_3$) δ 148.96, 135.71, 135.56, 134.54, 134.44, 130.47, 130.45, 129.53, 122.04, 122.02, 118.30, 118.15. C2, C5 and C11 atoms were coupled by N1 nitrogen nucleus respectively.

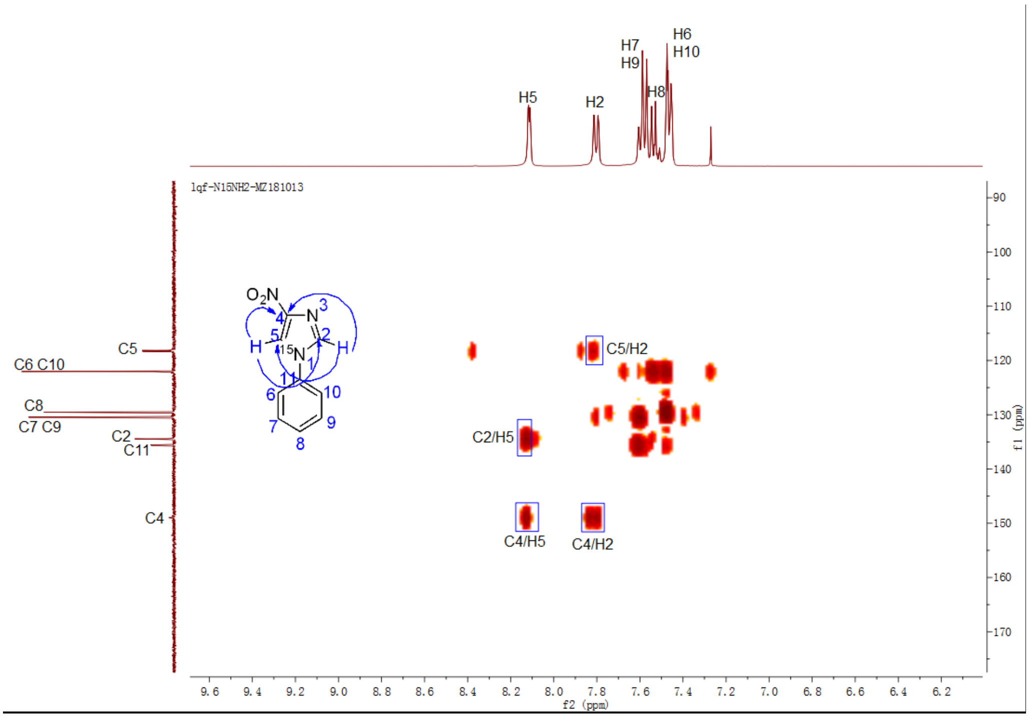

Supplementary Figure 22. ^{1}H-^{13}C HMBC spectrum of compound **1e**. Key correlations were highlighted.

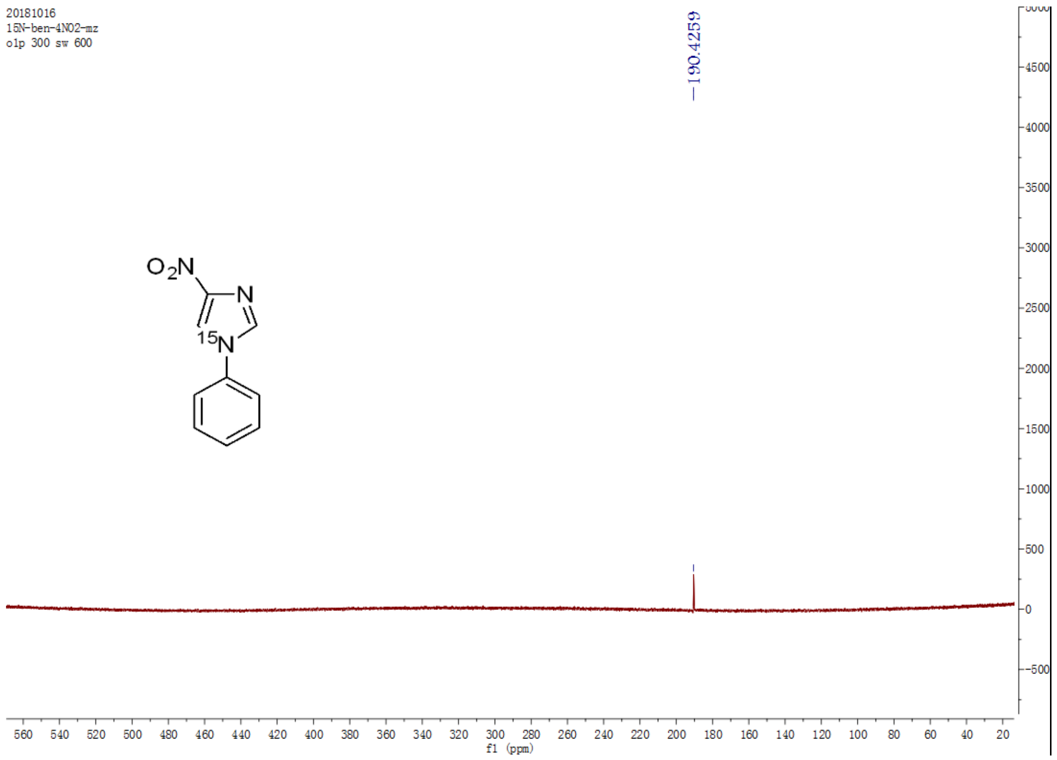

Supplementary Figure 23. ^{15}N NMR spectrum of compound **1e**.

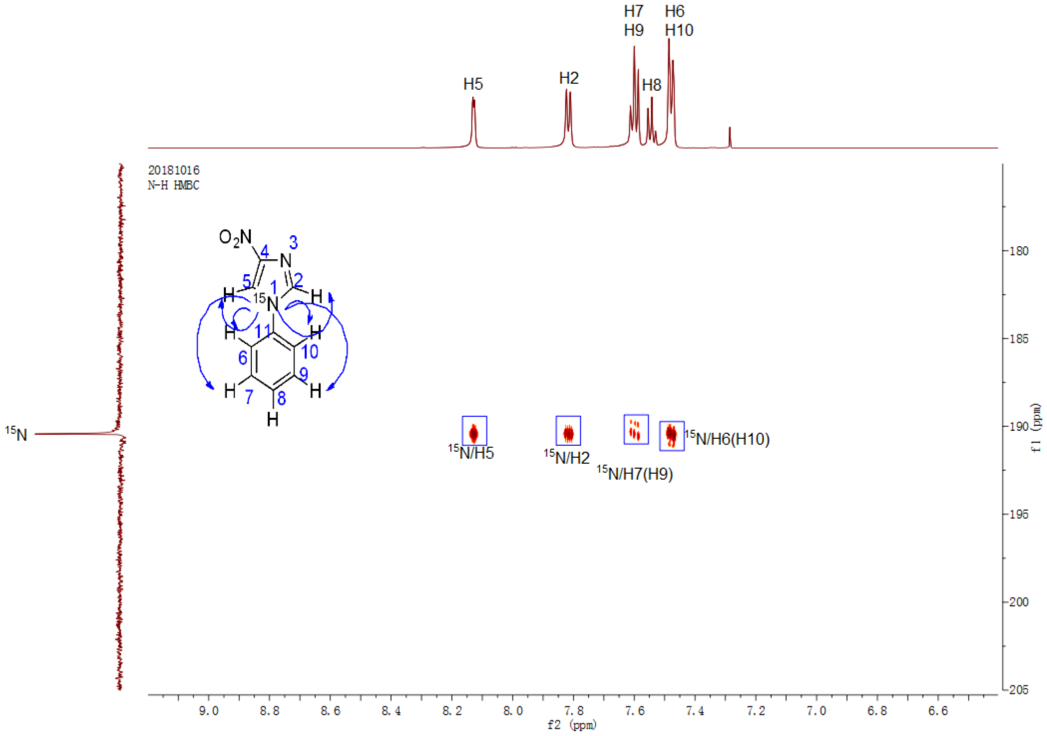

Supplementary Figure 24. ^{1}H-^{15}N HMBC spectrum of compound **1e**.

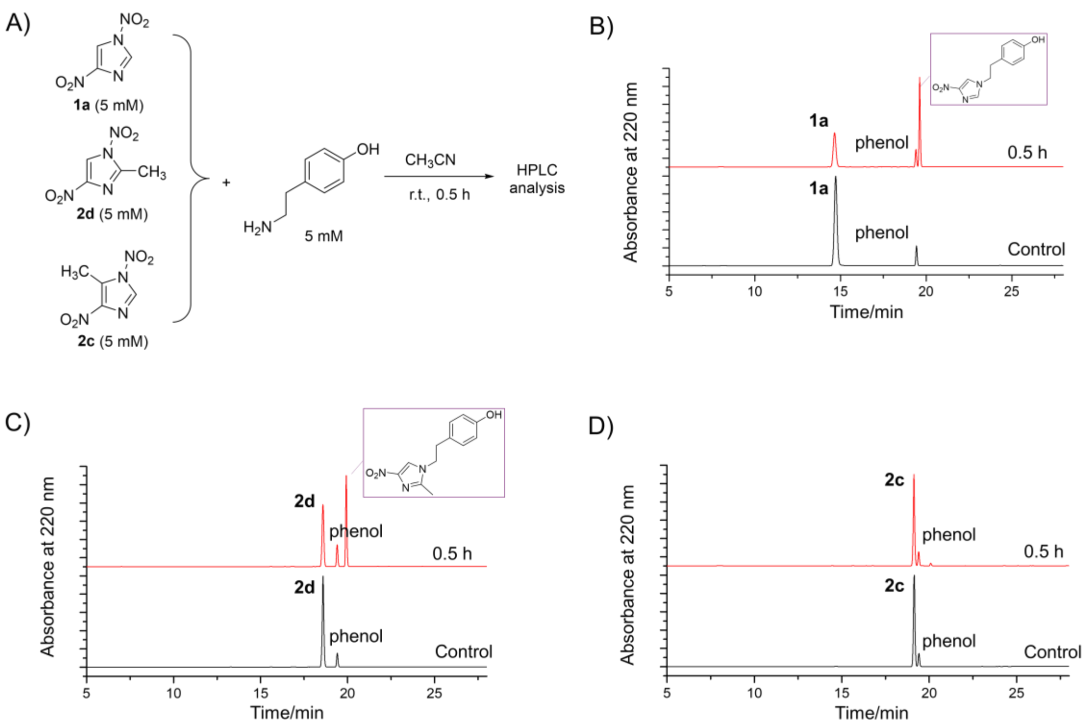

Supplementary Figure 25. Effects of methyl substitutions at C2 and C5 positions of 1,4-DNIms on their reactions with 4-(2-aminoethyl)phenol. Conditions: For each reaction, reactants (5 mM) were incubated in acetonitrile for 30 min. A) Independent reactionsof 4-(2-aminoethyl)phenol with three 1,4-DNIm derivatives. B) Reaction between 4-(2-aminoethyl)phenol and 1,4-DNIm **1a**. The conversion was 52% as determined by HPLC analysis. C) Reaction between 4-(2-aminoethyl)phenol and 2-methyl-1,4-DNIm **2d**. The conversion was 50% as determined by HPLC analysis. D) Reaction between 4-(2-aminoethyl)phenol and 5-methyl-1,4-DNIm **2c**. The conversion was 2% as determined by HPLC analysis.

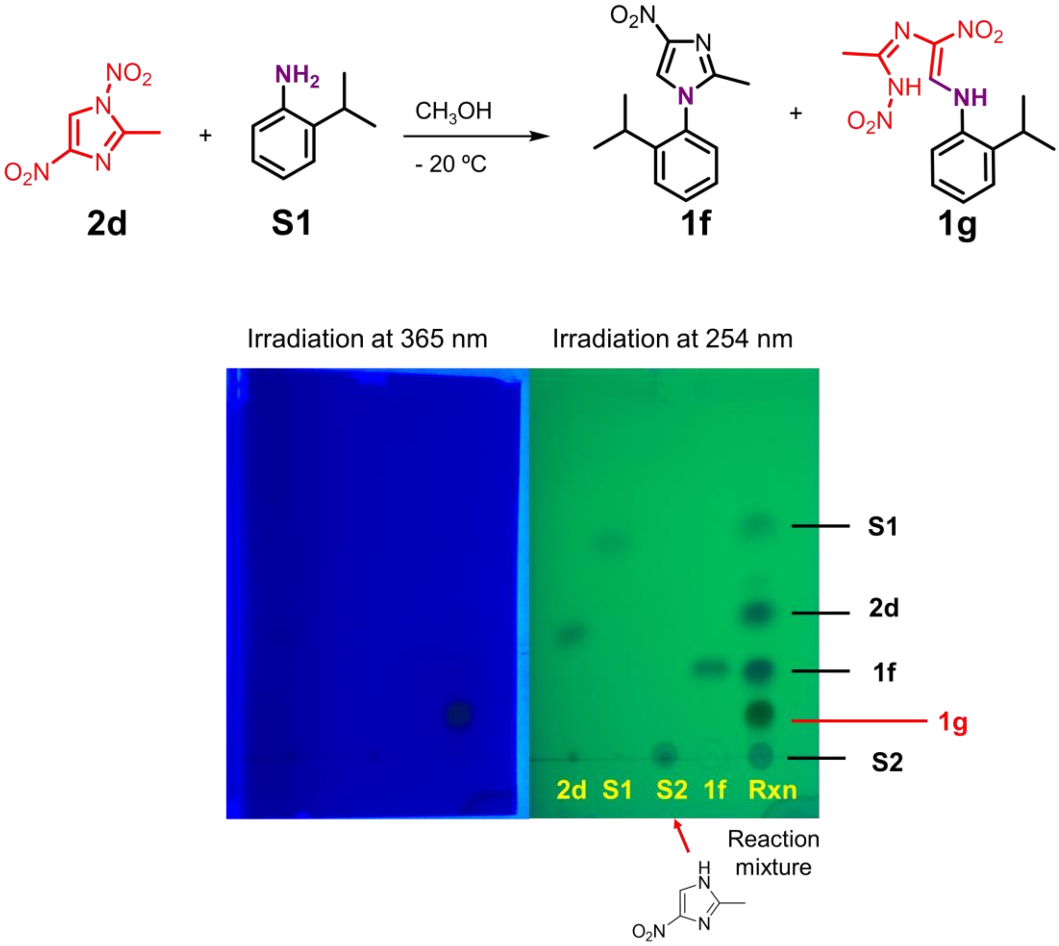

Supplementary Figure 26. TLC analysis of the reaction between 2-isopropylaninline **S1** and compound **2d**. Except the final product **1f** and by-product 4-nitroimidazole **S2**, a labile product **1g** was observed by both 254 nm and 365 nm irradiation. Eluent: PE/EA= 3/1(v/v).

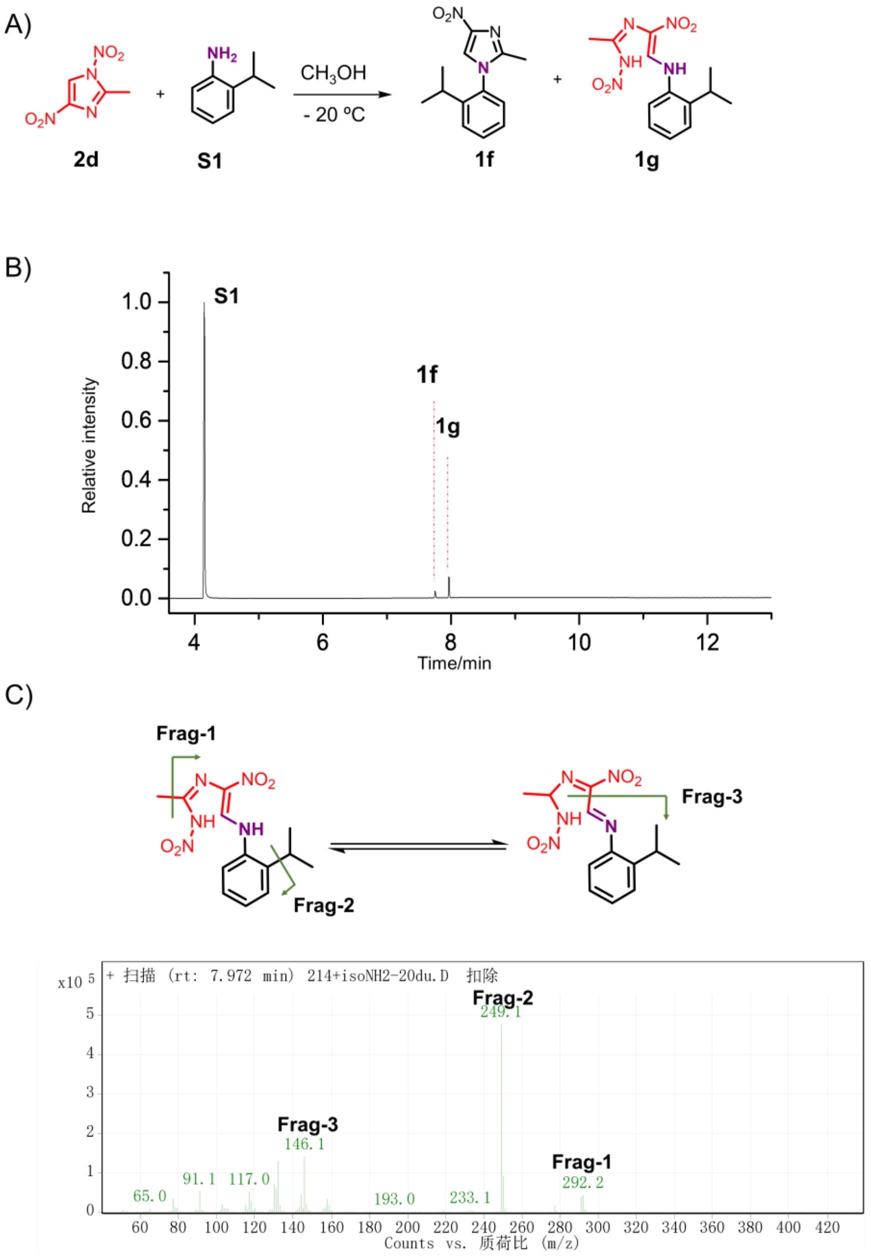

Supplementary Figure 27. GC-MS analysis of intermediate **1g**. A) Reaction scheme. B) GC chromatography of the reaction. C) Fragmentation of intermediate **1g**. **Frag-1**: expected m/z=292.1, observed 292.2; **Frag-2**: expected m/z=249.1, observed 249.1; **Frag-3**: expected m/z=146.1, observed 146.1.

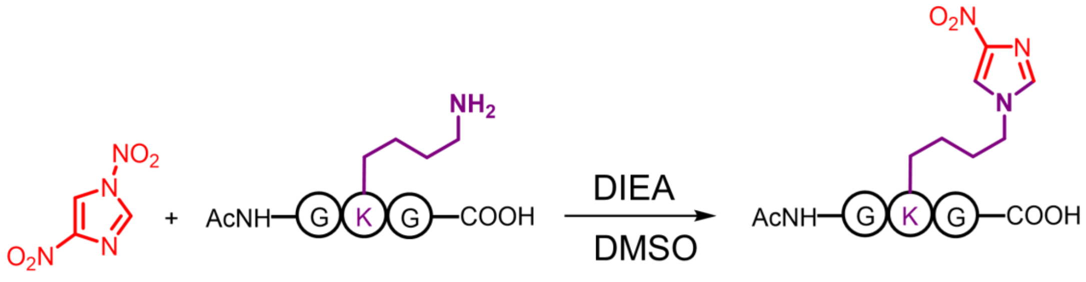

Supplementary Figure 28. Reaction scheme for preparing adduct **1d**. Compound **3a** (15.0 mg, 0.05 mmol) was dissolved in DMSO (2 mL) followed by the addition of DIEA (0.1 mmol) and compound **1a** (10 mg, 0.06 mmol). The mixture was stirred at r.t. for 3 h before quenched with diluted formic acid and subjected to preparative HPLC to give yellow solid (12 mg, 61% yield). See **Supplementary Figure 29-32** for NMR spectra.

^{1}H NMR (400 MHz, D_2O) δ 8.16 (d, J = 1.4 Hz, 1H), 7.70 (d, J = 1.3 Hz, 1H), 4.32 (dd, J = 8.8, 5.5 Hz, 1H), 4.10 (t, J = 6.9 Hz, 2H), 3.93 (d, J = 1.0 Hz, 2H), 3.86 (s, 2H), 2.00 (s, 3H), 1.90 – 1.78 (m, 3H), 1.76 – 1.67 (m, 1H), 1.40 – 1.22 (m, 2H).

^{13}C NMR (100 MHz, D_2O) δ 174.78, 174.20, 173.05, 171.56, 146.37, 137.53, 121.54, 53.17, 47.88, 42.38, 41.02, 30.17, 28.93, 21.53.

HRMS (ESI) m/z calcd. for $C_{15}H_{23}N_6O_7$ $[M+H]^+$ 399.1628, found 399.1622.

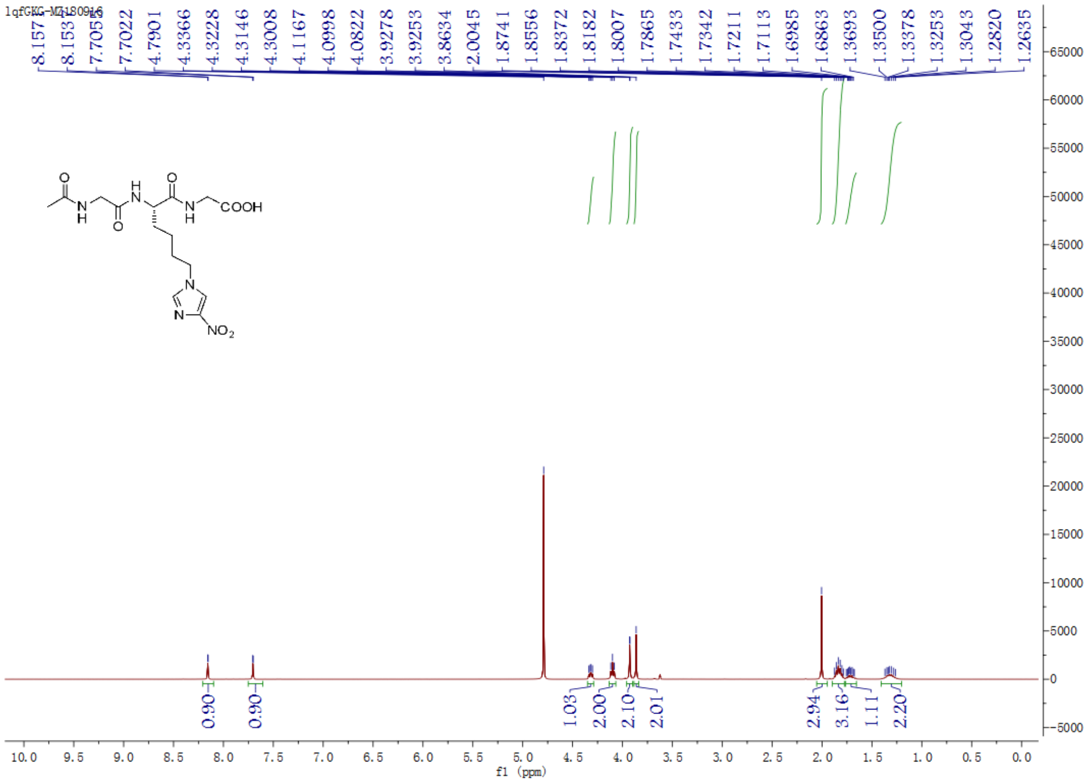

Supplementary Figure 29. ^{1}H NMR spectra of adduct **1d**.

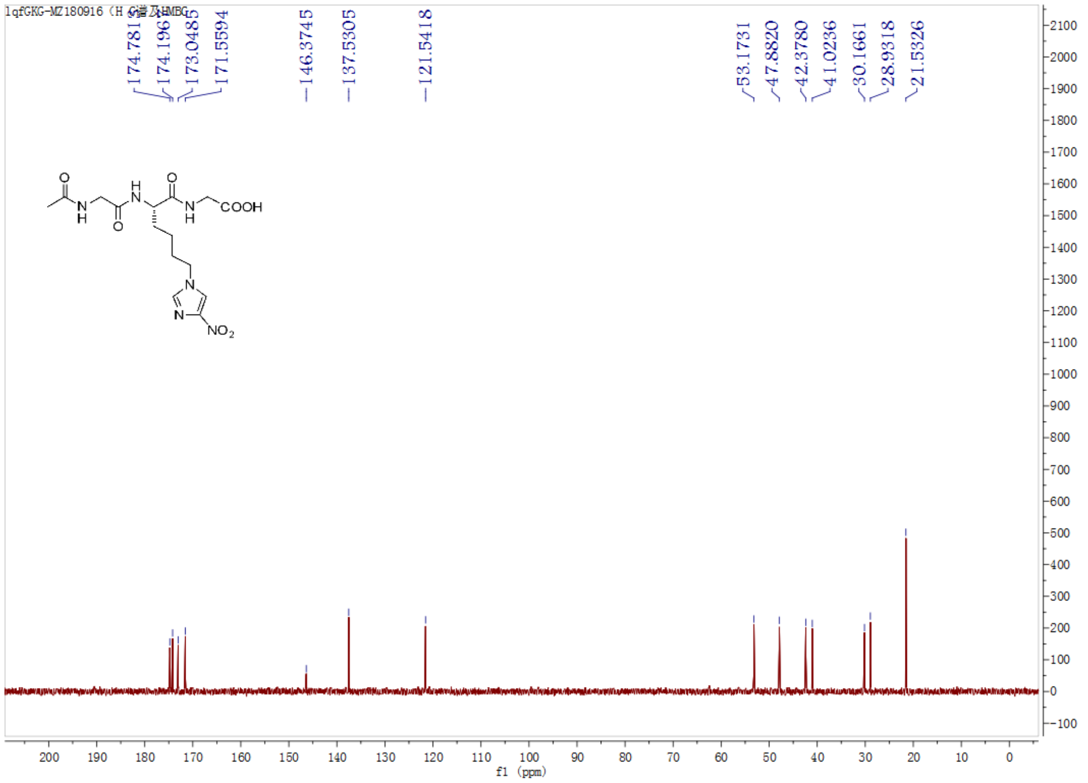

Supplementary Figure 30. ^{13}C NMR spectra of adduct **1d**.

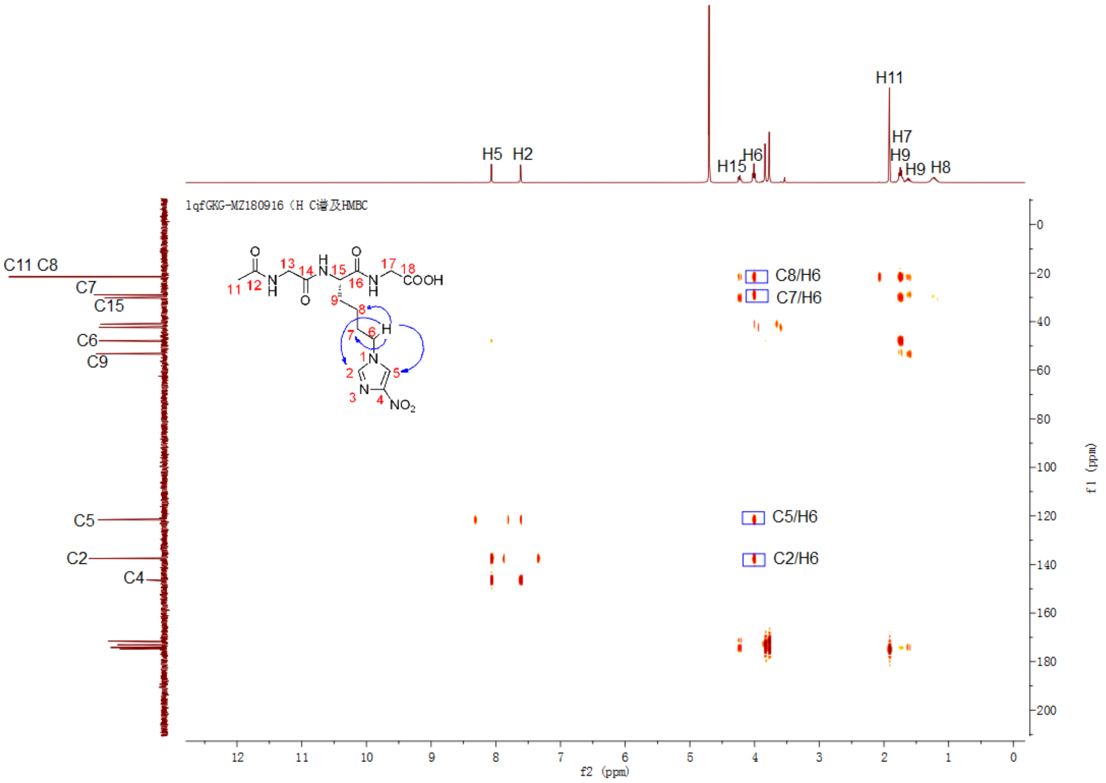

Supplementary Figure 31. ^{1}H-^{13}C HMBC spectrum of adduct **1d**. Key correlations are highlighted.

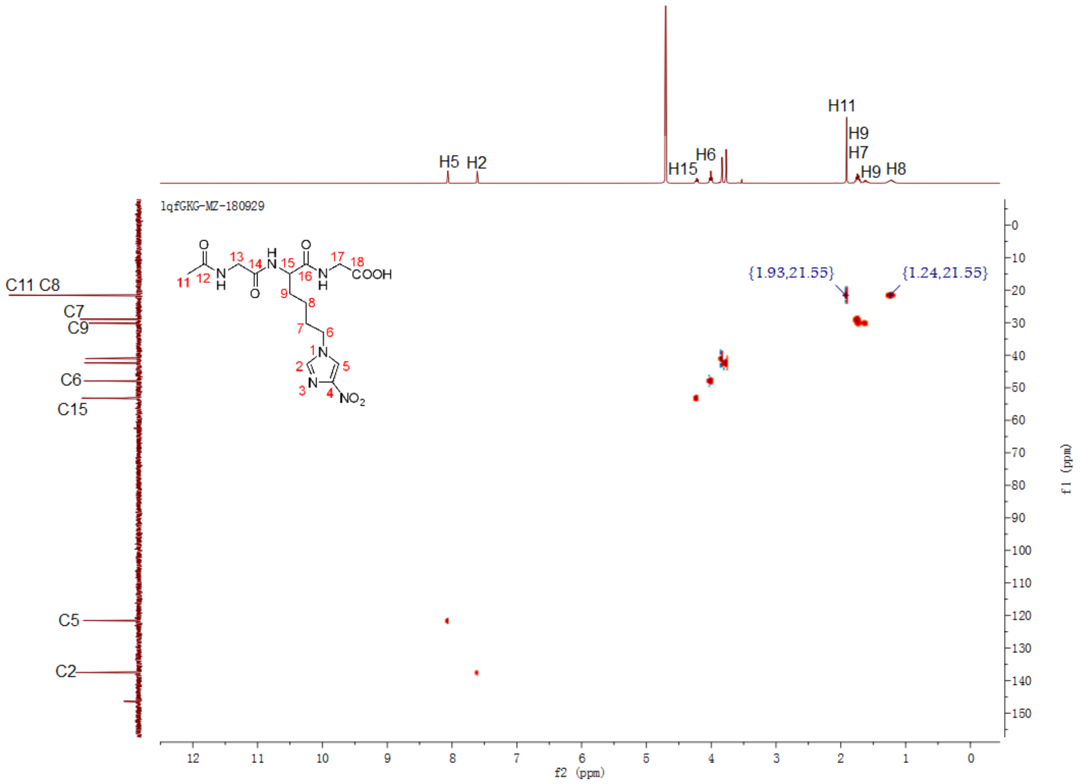

Supplementary Figure 32. ^{1}H-^{13}C HSQC spectrum of adduct **1d.** C11 and C8 nuclei have the same chemical shift, as determined by HMBC and HSQC NMR technique.

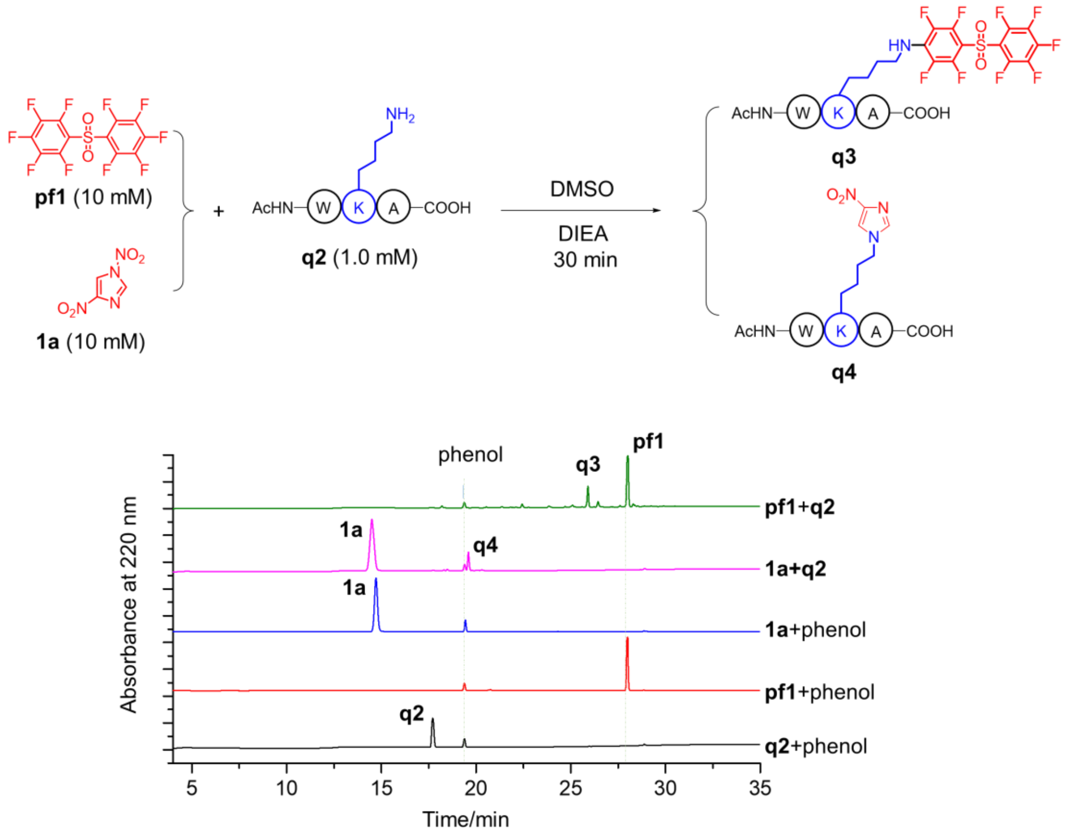

Supplementary Figure 33. Reactions between peptide **q2** and 1,4-DNIm **1a** or perfluoroaryl reagent **pf1**. Both reactions resulted in full conversion of peptide substrate **q2**.

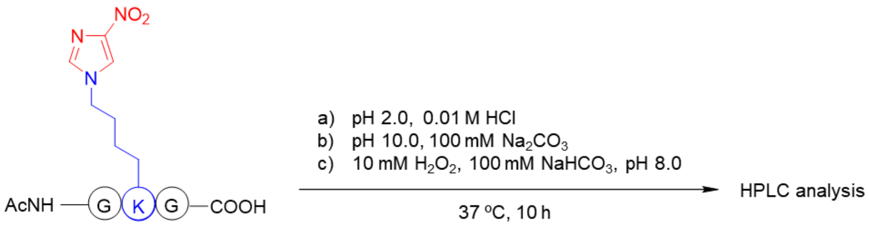

1d

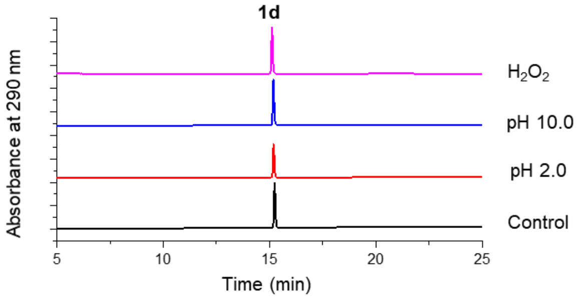

Supplementary Figure 34. Stability of adduct **1d** in buffers at pH 2.0 and 10.0, or in 10 mM H_2O_2 at 37 °C for 10 h. Gradient used： isocratic 2% CH_3CN for 5 min, then 2% to 90% CH_3CN in 25 min, then isocratic 90% CH_3CN for 5 min, then 90% to 2% CH_3CN in 5 min. Monitor wavelength: 290 nm.

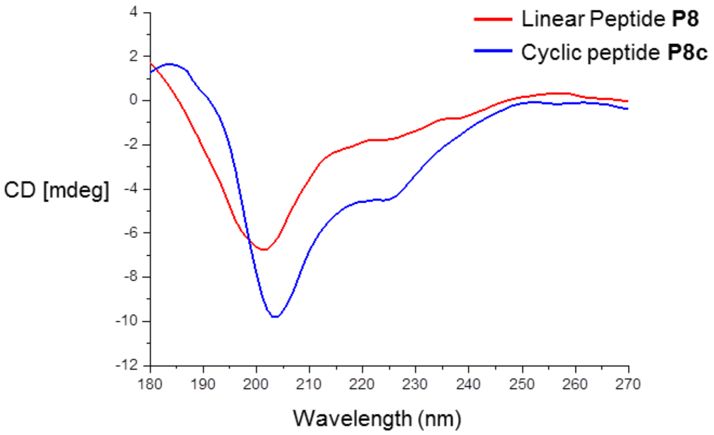

Supplementary Figure 35. CD spectrum of linear and cyclic peptide (P8 and P8c) at 25 °C. Cyclic peptide **P8c** showed enhanced absorption at 208 nm and 222 nm in comparison with linear peptide **P8**, which indicated α-helix content of the linear peptide was increased after cyclization with the bis(1,4-DNIm) reagent.

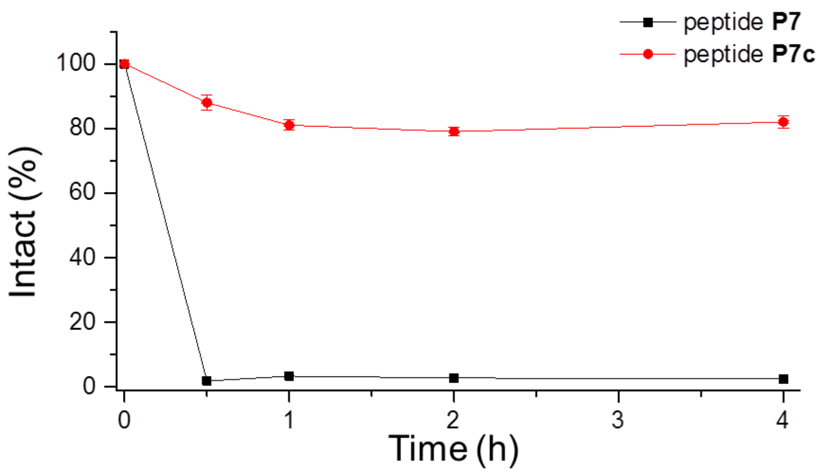

Supplementary Figure 36. Cyclized **P7** peptide (**P7c**) exhibited enhanced resistance towards proteolytic degradation. See **Supplementary Figure 101 and 102** for HPLC data.

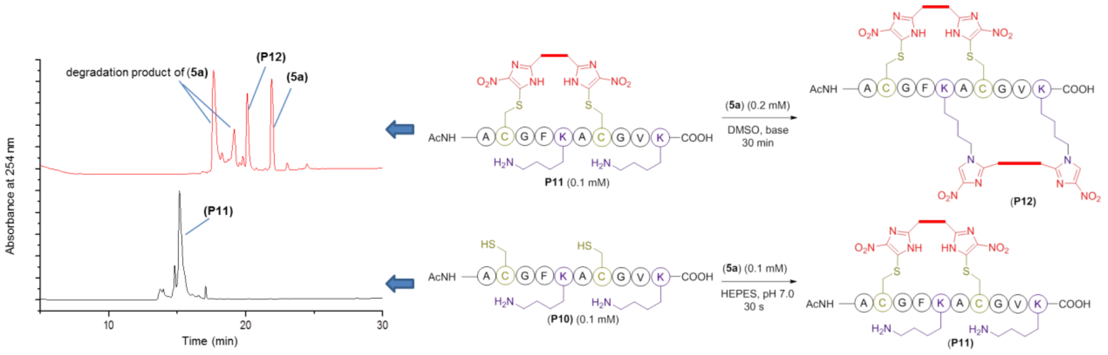

Supplementary Figure 37. Bicyclic peptide (**P12**) was prepared from linear peptide (**P10**) by consecutive macrocyclization with compound **5a.** The reaction mixture for the preparation of monocyclic peptide **P11** was directly lyophilized before subjected to the next step. Linear peptide **P10** has a retention time less than 1 min and therefore is not shown.

P11

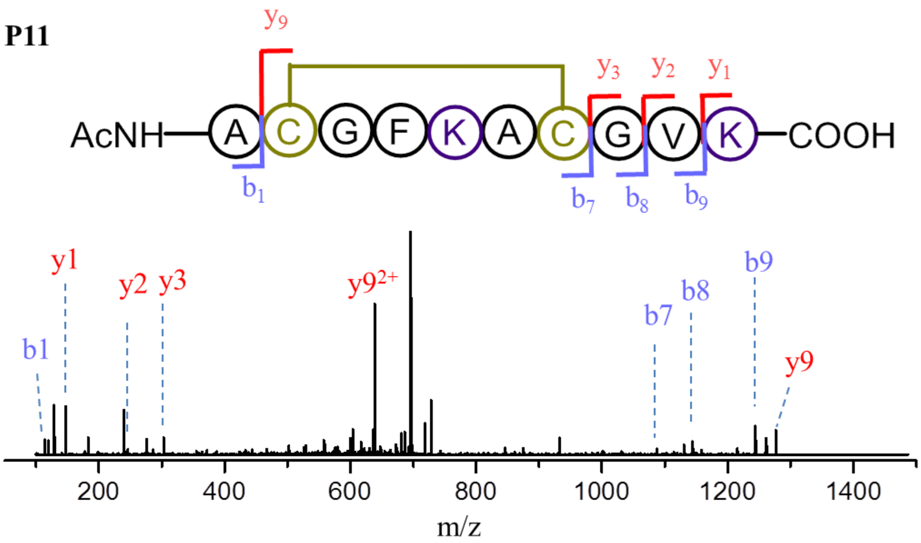

	Calculated m/z	Observed m/z	Relative deviation (ppm)
b1	114.0550	114.0559	7.8947
b7	1087.3358	1087.3397	3.5878
b8	1144.3572	1144.3677	9.1783
b9	1243.4256	1243.4368	9.0104
y9	1276.4835	1276.4946	8.6990
y3	303.2027	303.2039	3.9604
y2	246.1813	246.1809	-1.6260
y1	147.1129	147.1131	1.3605

Supplementary Figure 38. Tandem MS analysis of compound **P11**.

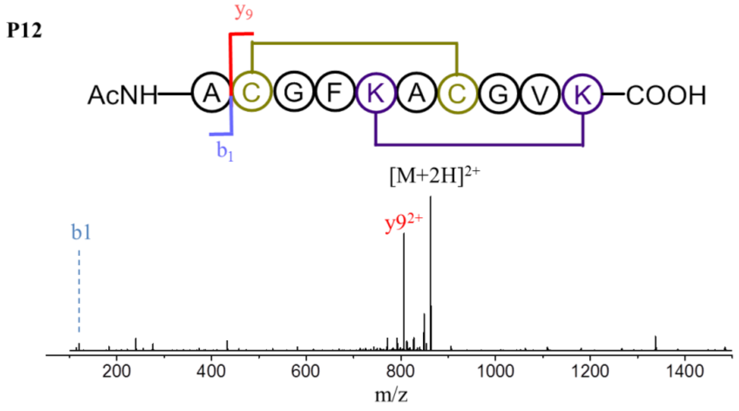

	Calculated m/z	Observed m/z	Relative deviation (ppm)
b1	114.0550	114.0540	-9.1228
$y9^{2+}$	805.7546	805.7589	5.2795

Supplementary Figure 39. Tandem MS analysis of compound **P12**.

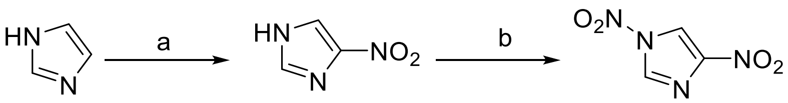

1a

Supplementary Figure 40. Synthesis of compound 1,4-dinitro-1*H*-imidazole (**1a**). Conditions: a) 98% H_2SO_4, 68% HNO_3, 60% yield; b) Fuming HNO_3, CH_3COOH, Ac_2O, 90% yield. Compound 4-nitro-1*H*-imidazole was prepared according to the reported method and the ^{1}H NMR data is consistent with the reference[1]. ^{1}H NMR (400 MHz, DMSO-*d*6) δ 13.25 (s, 1H), 8.33 (s, 1H), 7.86 (s, 1H). Compound 1,4-dinitro-1*H*-imidazole (**1a**) was obtained by nitration of compound 4-nitro-1*H*-imidazole following the reported method and the ^{1}H NMR data is consistent with the reference[2]. ^{1}H NMR (400 MHz, $CDCl_3$) δ 8.51 (d, *J* = 1.6 Hz, 1H), 8.38 (d, *J* = 1.6 Hz, 1H).

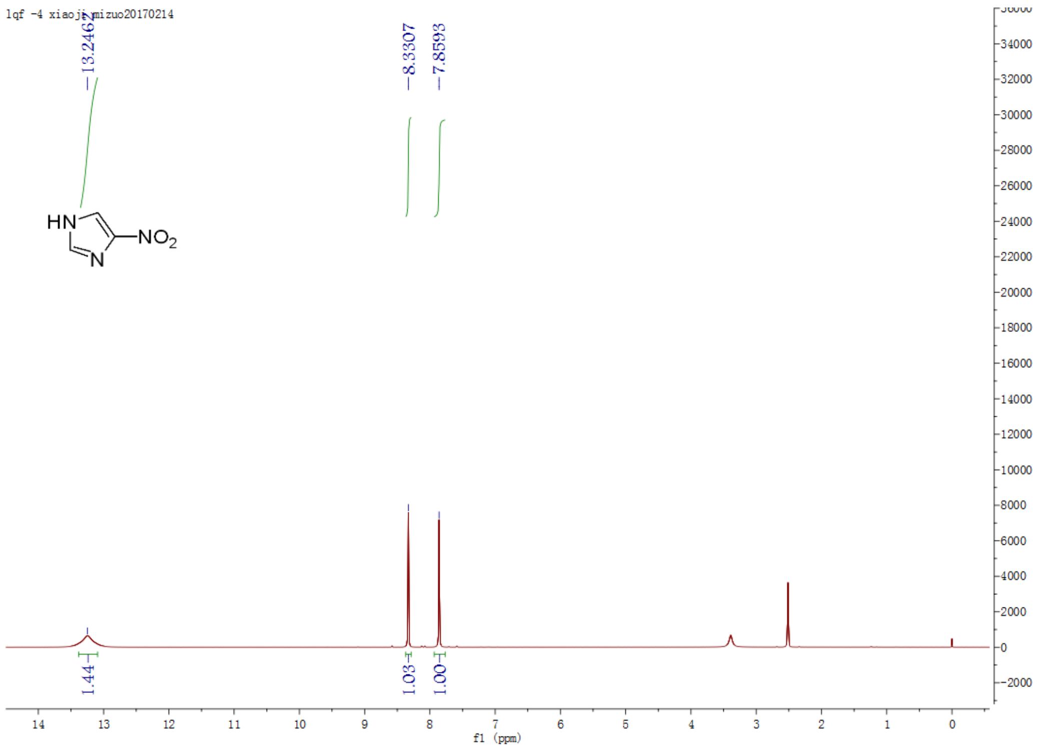

Supplementary Figure 41. ^{1}H NMR spectrum of compound 4-nitro-1*H*-imidazole

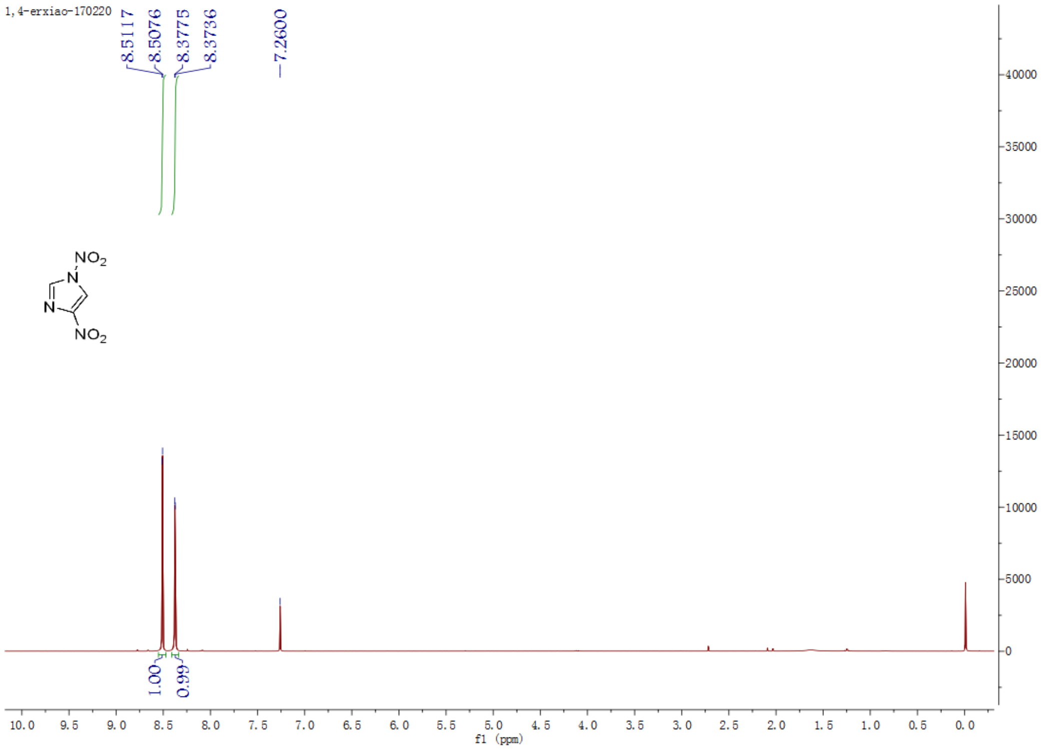

Supplementary Figure 42. ^{1}H NMR spectrum of compound 1,4-dinitro-1*H*-imidazole (**1a**).

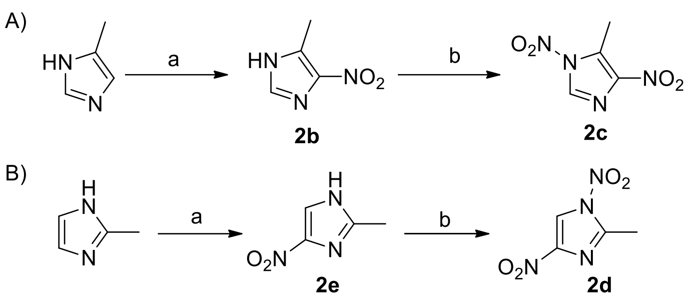

Supplementary Figure 43. Synthesis of compound 5-methyl-1,4-dinitro-1*H*-imidazole (**2c**) and 2-methyl-1,4-dinitro-1*H*-imidazole (**2d**). a) 98% H_2SO_4, 68% HNO_3, 70% yield for **2b** and 52% yield for **2e**; b) Fuming HNO_3, CH_3COOH, Ac_2O, 23% yield for **2c** and 91% yield for **2d**.

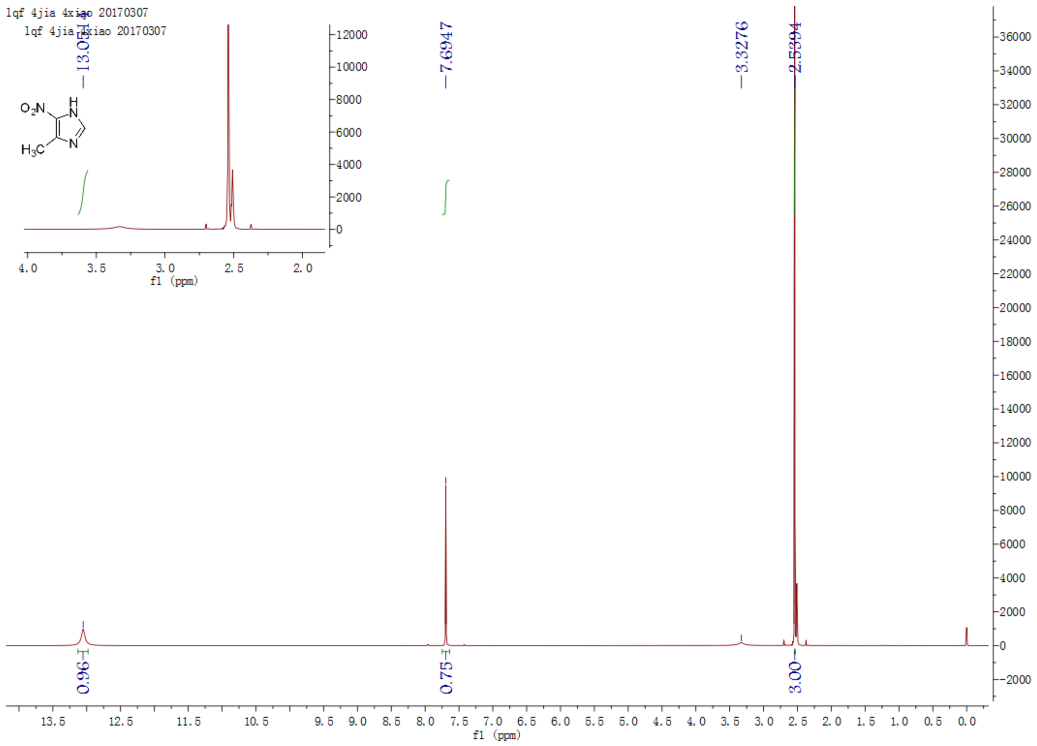

Supplementary Figure 44. ^{1}H NMR spectrum of compound **2b**. ^{1}H NMR (400 MHz, DMSO-*d*6) δ 13.05 (s, 1H), 7.69 (s, 1H), 2.54 (s, 3H).

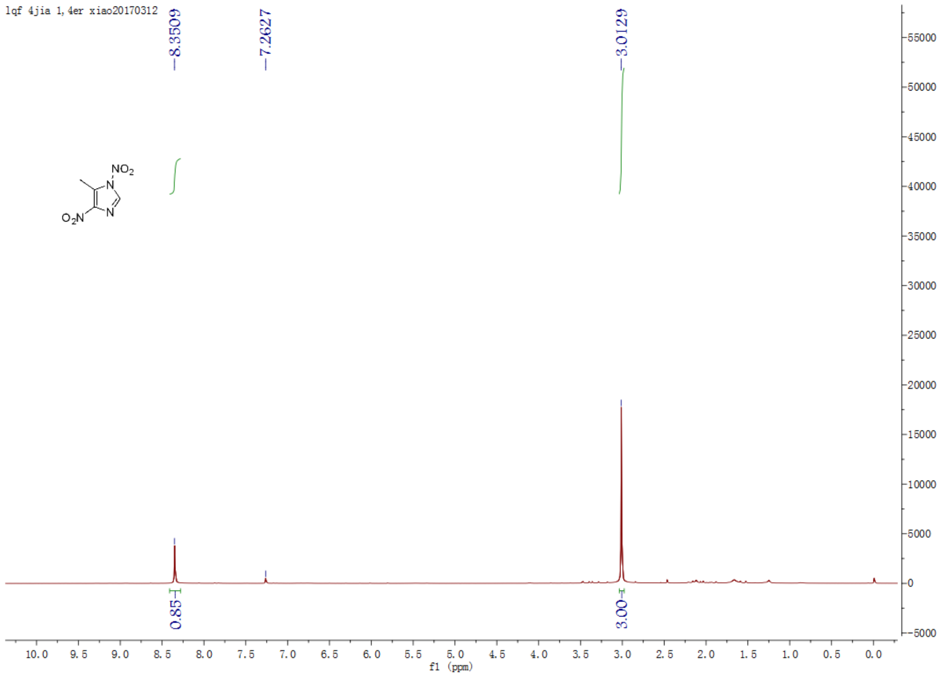

Supplementary Figure 45. ^{1}H NMR spectrum of compound **2c**. ^{1}H NMR (400 MHz, $CDCl_3$) δ 8.35 (s, 1H), 3.01 (s, 3H).

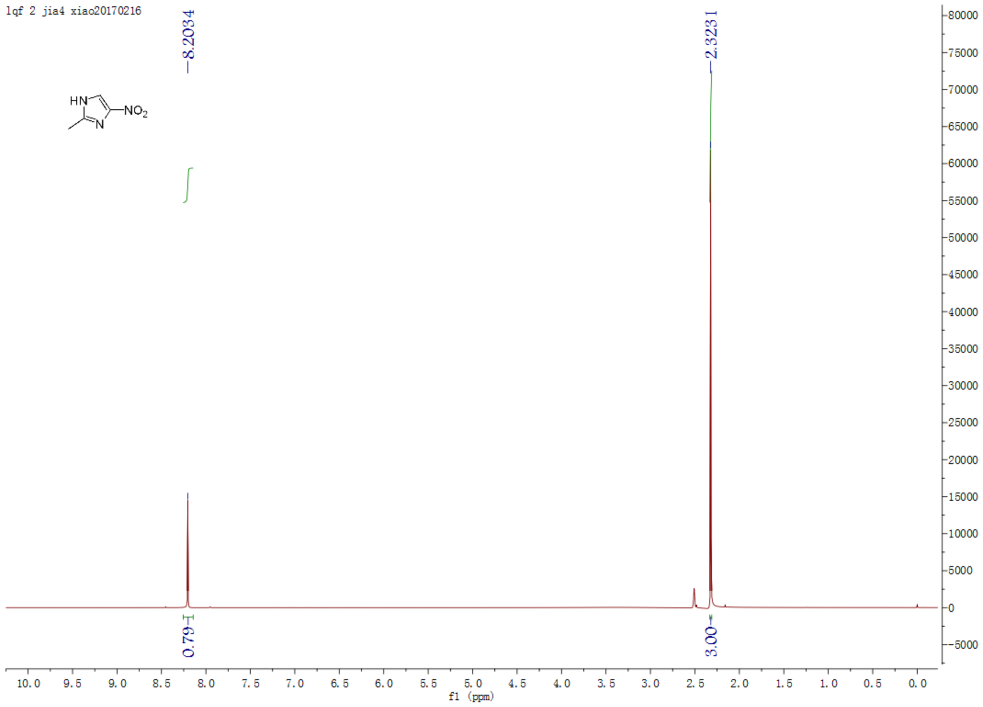

Supplementary Figure 46. ^{1}H NMR spectrum of compound **2e**. ^{1}H NMR (400 MHz, DMSO-*d6*) δ 8.20 (s, 1H), 2.32 (s, 3H).

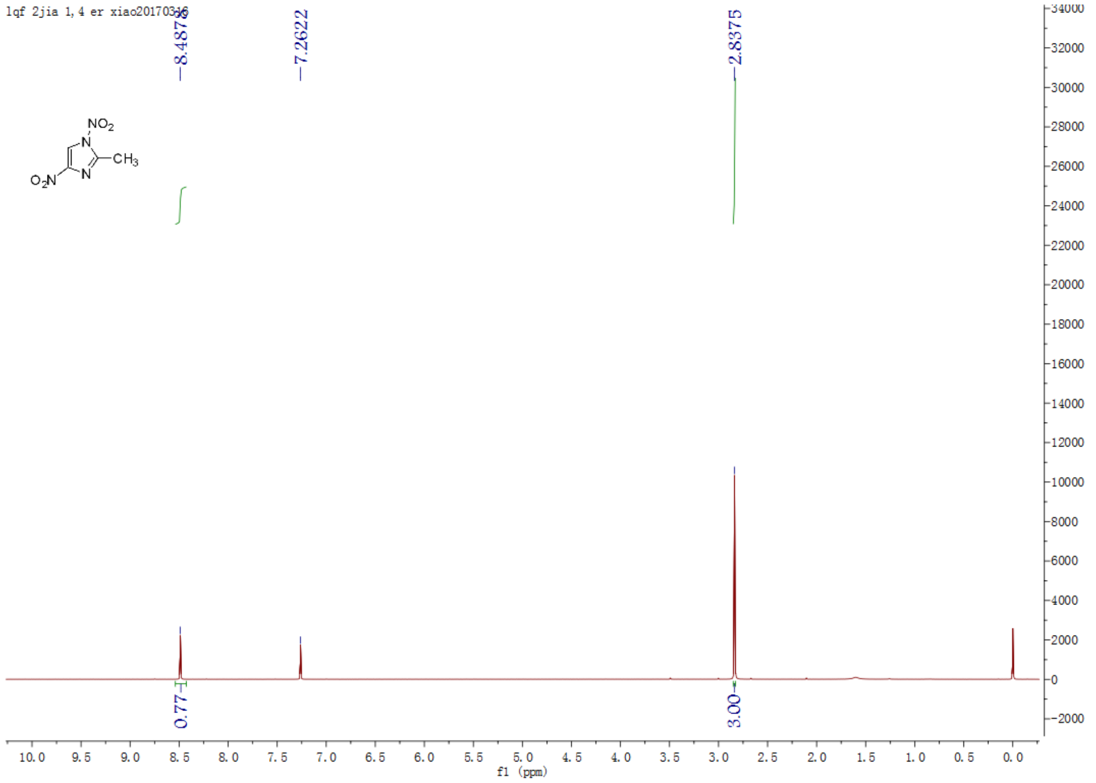

Supplementary Figure 47. ^{1}H NMR spectrum of compound **2d**. ^{1}H NMR (400 MHz, $CDCl_3$) δ 8.49 (s, 1H), 2.84 (s, 3H).

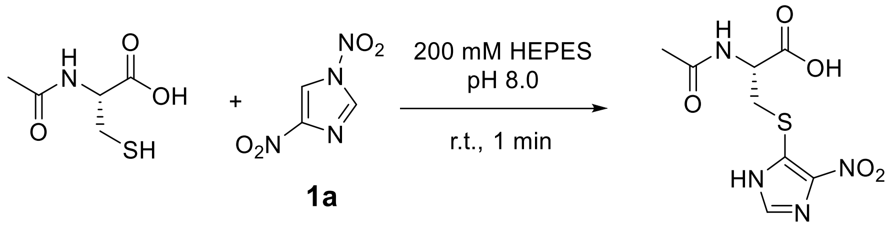

1a **1b**

Supplementary Figure 48. Synthesis of N-acetyl-L-cysteine-(4-nitroimidazole) adduct **1b**. *N*-acetyl-L-cysteine (0.041 g, 0.25 mmol) was dissolved in 10 mL 200 mM HEPES buffer (pH 8.0) at room temperature. Compound **1a** (0.048 g, 0.3 mmol) was then added to the solution and incubated for 1 min before acidified with formic acid. The resulting mixture was subject to preparative HPLC to afford compound **1b** as dark red solid (0.049 g, 72%).

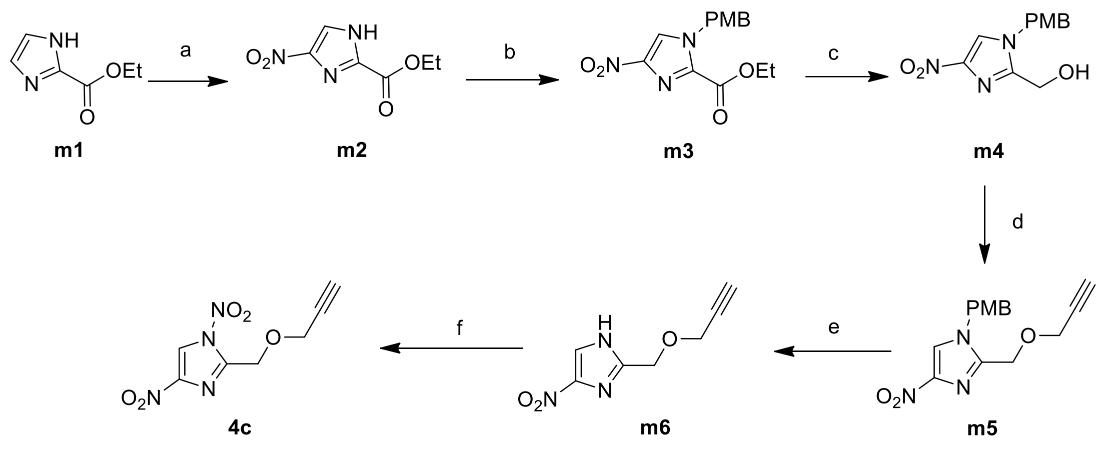

Supplementary Figure 49. Synthesis of compound 1,4-dinitro-2-((prop-2-yn-1-yloxy)methyl)-1*H*- imidazole (**4c**). a) Concentrated H_2SO_4, fuming HNO_3, 75% yield; b) K_2CO_3, DMF, PMBCl, N_2, 115 ^oC, 84% yield; c) $LiAlH_4$, dry THF, -5 - 0 ^oC, 47% yield; d) NaOH, DMF, r.t., 90%; e) TFA, anisole, 80 ^oC, 99% yield; f) CH_3COOH, Ac_2O, fuming HNO_3, -5 - 0 ^oC, 74%.

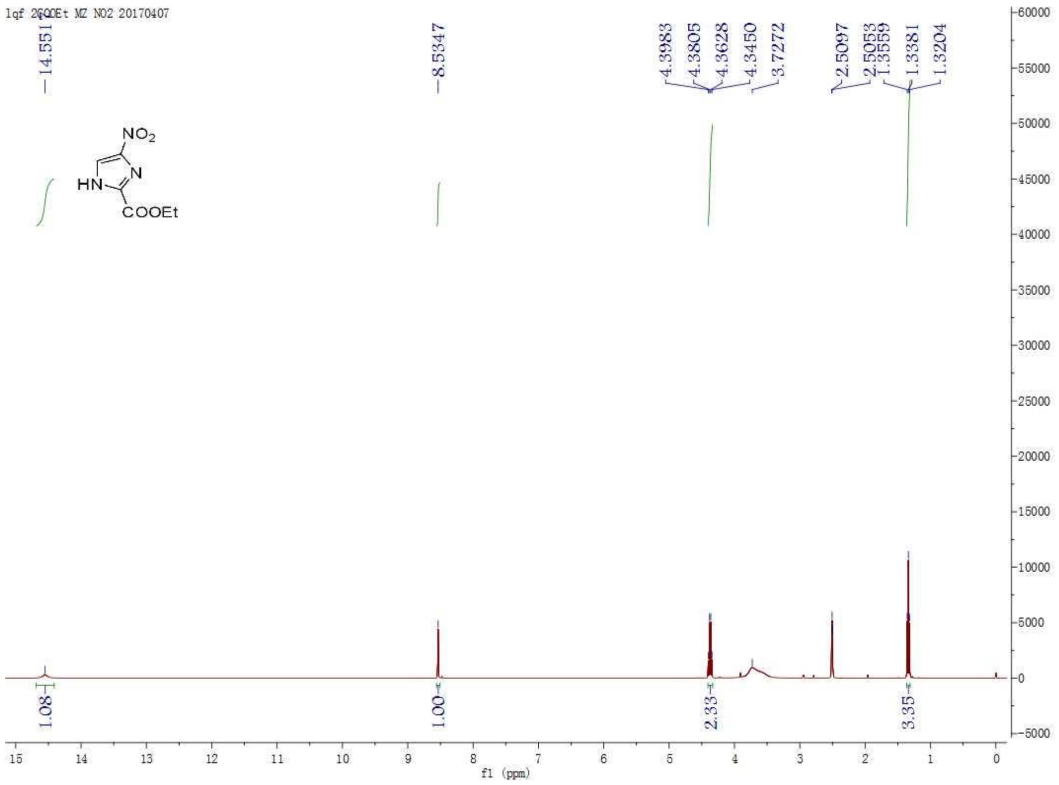

Supplementary Figure 50. ^{1}H NMR spectrum of compound **m2**. A reaction tube was charged with ethyl 1H-imidazole-2-carboxylate **m1** (1.15 g, 8.20 mmol) and concentrated H_2SO_4 (5.0 mL, 93 mmol) at 0 ^oC. To the solution was added fuming HNO_3 (1.0 mL, 24 mmol) dropwise with the aid of a syringe. The reaction was stirred at 60 °C for 7 h before cooled to room temperature. The reaction mixture was then added to ice crush and the resulting precipitate was filtered and dried under vacuum to yield **m2** as a white solid (1.14 g, 75% yield). ^{1}H NMR (400 MHz, DMSO-*d*6) δ 14.55 (s, 1H), 8.53 (s, 1H), 4.37 (q, 2H), 1.34 (t, 3H).

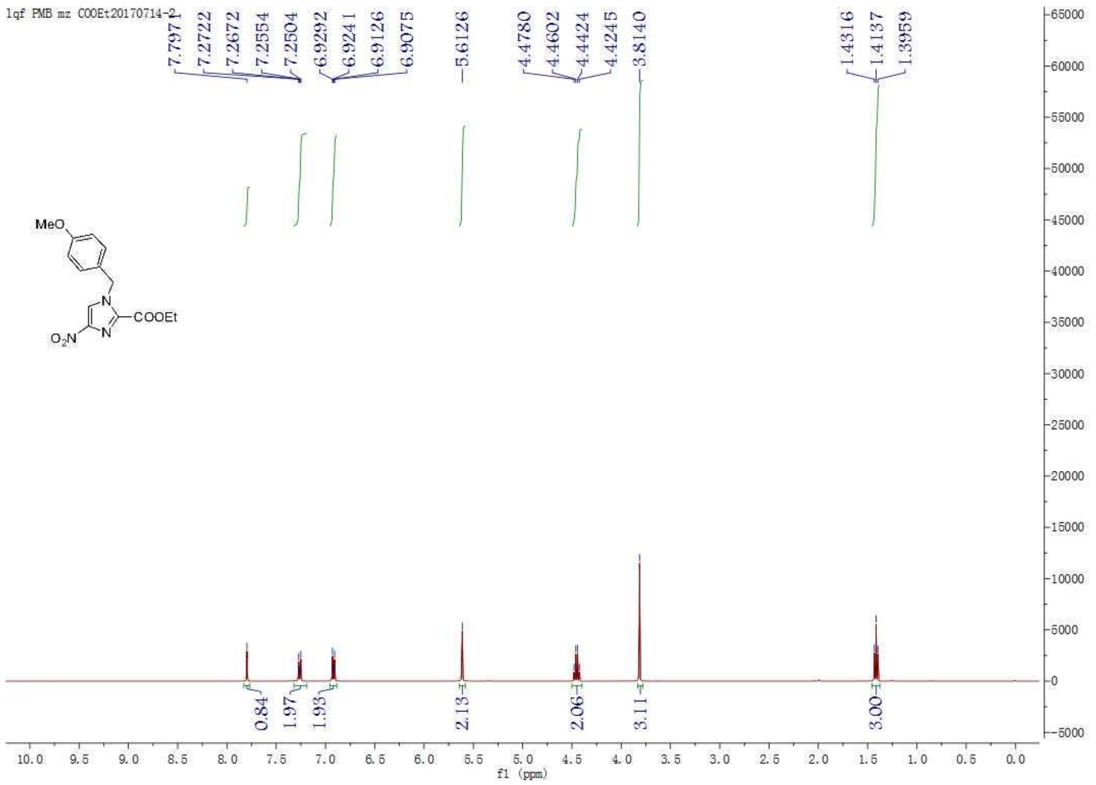

Supplementary Figure 51. ^{1}H NMR spectrum of compound **m3**. To a 25 mL two-neck round bottom flask equipped with a condenser and a magnetic stirring bar was added potassium carbonate (0.289 g, 2.10 mmol) and compound **m2** (0.485 g, 2.62 mmol). *p*-Methoxybenzyl chloride (0.39 mL, 2.9 mmol) in dry DMF (3.0 mL) was added via a *syringe* under nitrogen atmosphere. The reaction was stirred at 115 ^{o}C overnight before cooled in an ice bath. The resulting precipitate was filtered and washed with ethyl acetate. The filtrate was combined, concentrated and subject to flash column chromatography (PE/EA = 10/3, v/v) to afford compound **m3** as a white solid (0.67 g, 84% yield).

^{1}H NMR (400 MHz, $CDCl_3$) δ 7.80 (s, 1H), 7.27-7.25 (m, 2H), 6.93 - 6.91 (m, 2H), 5.61 (s, 2H), 4.45 (q, J = 7.2 Hz, 2H), 3.81 (s, 3H), 1.41 (t, J = 7.2 Hz, 3H).

^{13}C NMR (100 MHz, $CDCl_3$) δ 160.16, 158.30, 146.20, 134.60, 129.97, 125.93, 123.29, 114.69, 62.59, 55.35, 52.67, 14.14.

HRMS (ESI) m/z calcd. for $C_{14}H_{15}N_3NaO_5$ $[M+Na]^+$ 328.0909, found 328.0904.

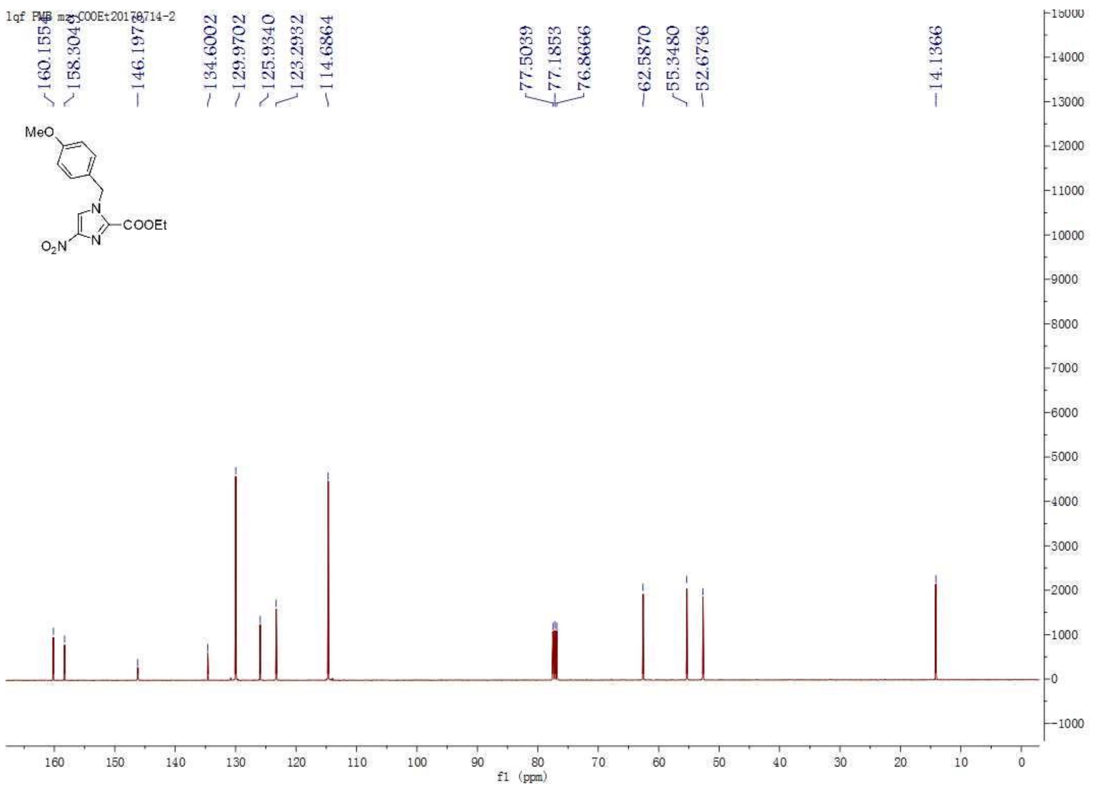

Supplementary Figure 52. ^{1}H NMR spectrum of compound **m3**.

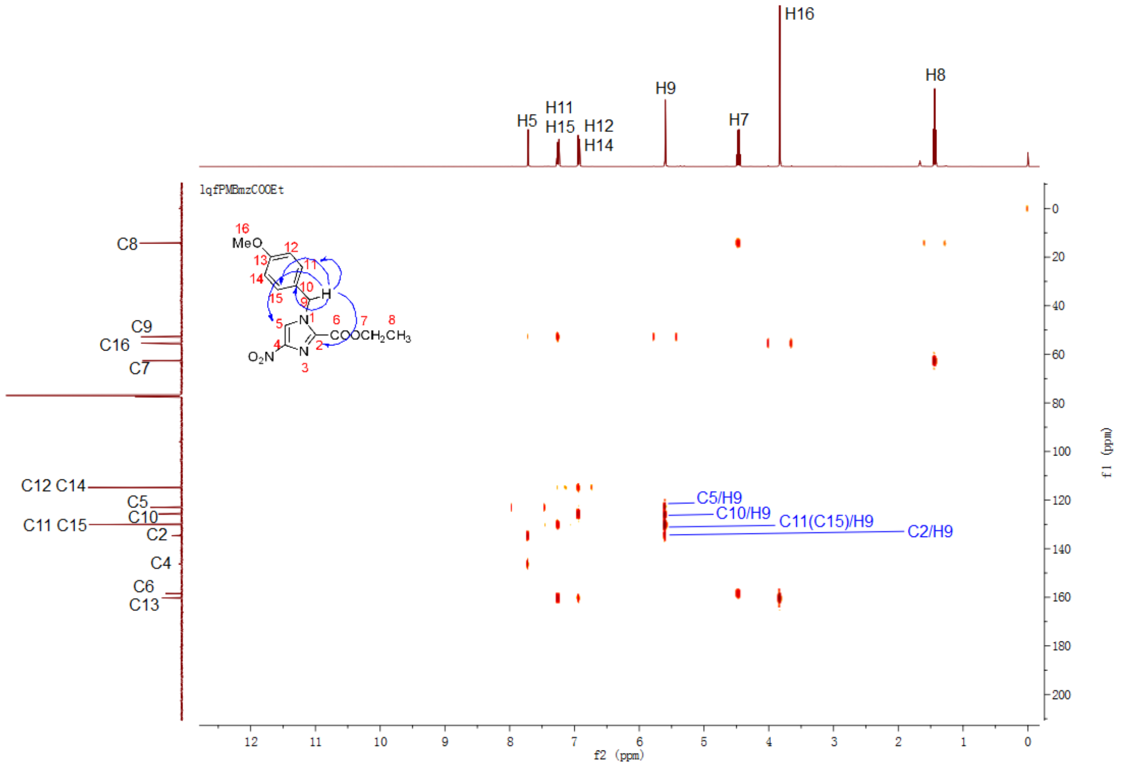

Supplementary Figure 53. HMBC analysis of compound **m3**. Key correlations were highlighted.

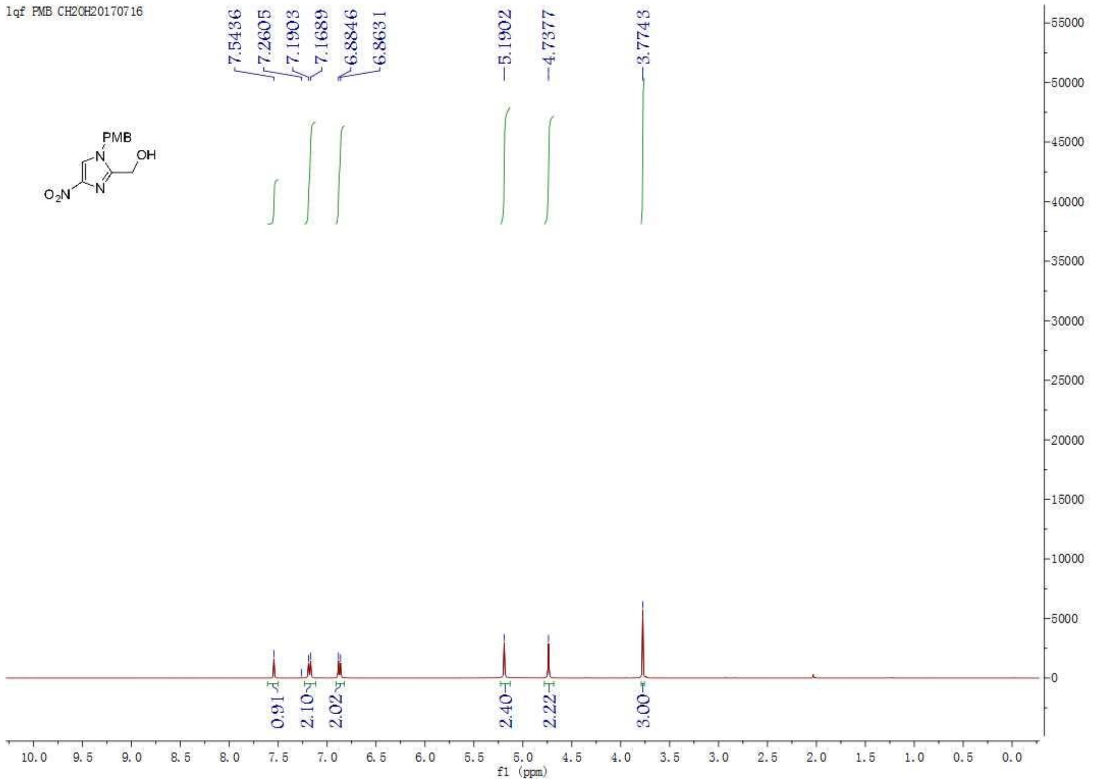

Supplementary Figure 54. ^{1}H NMR spectrum of compound **m4**. To a round bottom flask cooled in ice bath was added compound **m3** (0.305 g, 1.0 mmol) and dry THF (6 mL). The solution was stirred vigorously while $LiAlH_4$ (0.057 g, 1.5 mmol) was added. The reaction was quenched after 2 h with ice water (2.0 mL) and saturated $NaHCO_3$ (3.0 mL) successively at 0 °C. The resulting bright red slurry was filtered and washed with ethyl acetate. The filtrate was dried over anhydrous sodium sulfate, concentrated and subject to flash column chromatography (PE:EA=1:1, v:v) to afford compound **m4** as a white solid (0.123 g, 47% yield).

^{1}H NMR (400 MHz, $CDCl_3$) δ 7.54 (s, 1H), 7.19 - 7.17 (m, 2H), 6.88 - 6.86 (m, 2H), 5.19 (s, 2H), 4.74 (s, 2H), 3.77 (s, 3H).

^{13}C NMR (100 MHz, $CDCl_3$) δ 160.11, 147.05, 145.72, 129.73, 125.64, 120.80, 114.77, 56.51, 55.38, 50.62.

HRMS (ESI) m/z calcd for $C_{12}H_{13}N_3NaO_4$ $[M+Na]^+$ 286.0804, found 286.0797.

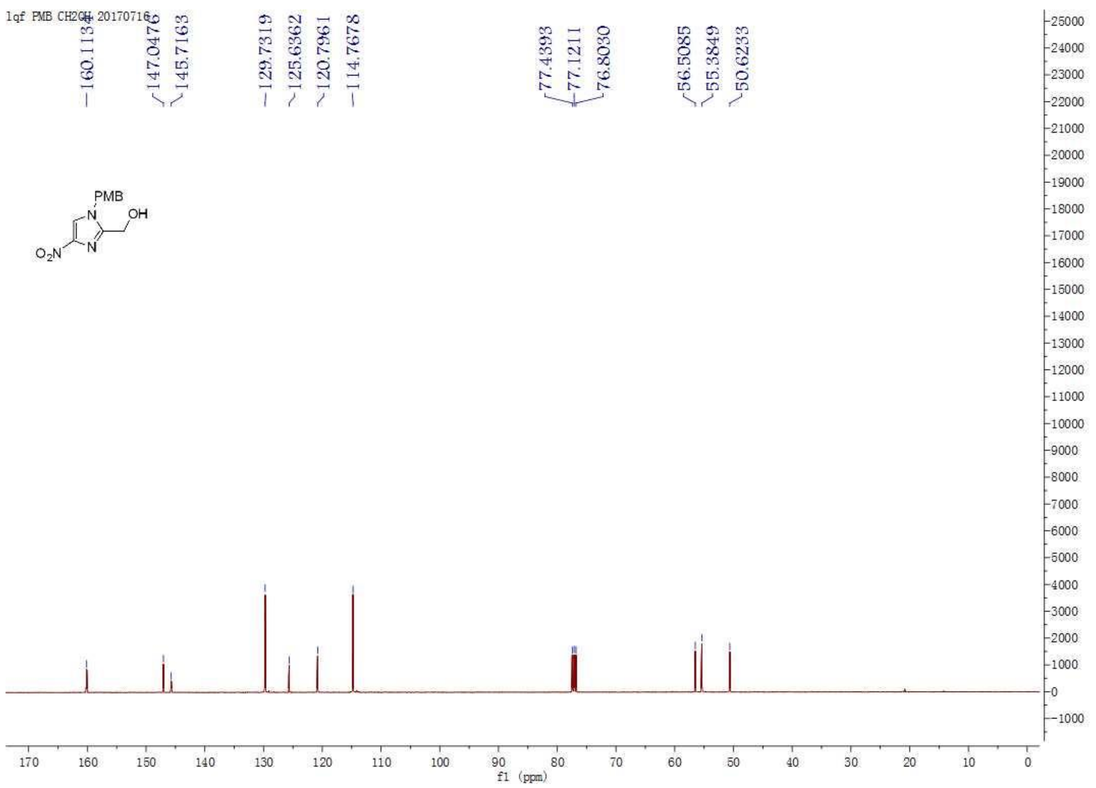

Supplementary Figure 55. ^{13}C NMR spectrum of compound **m4**.

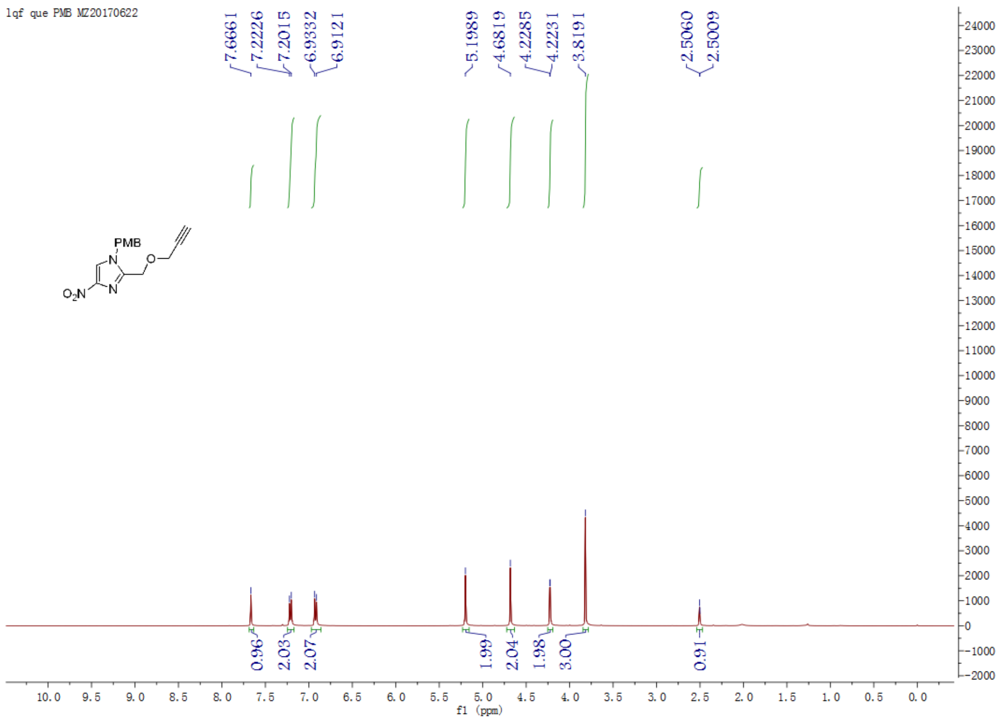

Supplementary Figure 56. ^{1}H NMR spectrum of compound **m5**. To a 25 mL single-neck round bottom flask was added DMF (1.5 mL), compound **m4** (0.078 g, 0.30 mmol) and NaOH (0.020 g, 0.45 mmol)**.** The mixture was stirred several minutes before 3-bromopropyne (0.032 mL, 0.37 mmol) was added. The reaction continued for 5 h at room temperature, diluted with DCM (70 mL) and washed with saturated NH_4Cl solution (5 mL×2) and brine (5 mL×2). The organic layer was dried with anhydrous Na_2SO_4 and concentrated in vacuum and subject to flash column chromatography (PE/EA=3/1, v/v) to afford compound **m5** as white solid (0.081 g, 90% yield).

^{1}H NMR (400 MHz, $CDCl_3$) δ 7.67 (s, 1H), 7.21 (d, *J* = 8.4 Hz, 2H), 6.92 (d, *J* = 8.4 Hz, 2H), 5.20 (s, 2H), 4.68 (s, 2H), 4.22 (d, *J* = 2.2 Hz, 2H), 3.82(s, 3H), 2.50 (t, *J* = 2.2 Hz, 1H).

HRMS (ESI) m/z calcd for $C_{15}H_{15}N_3NaO_4$ $[M+Na]^+$ 324.0960, found 324.0955.

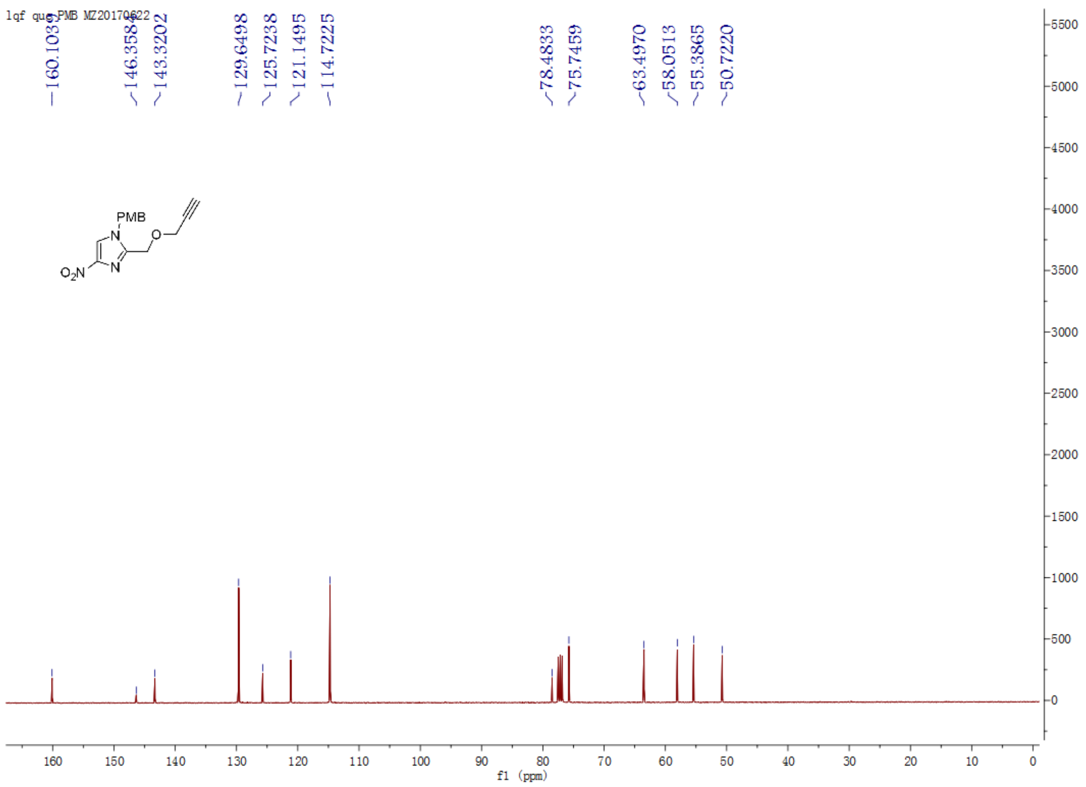

Supplementary Figure 57. ^{13}C NMR spectrum of compound **m5**. ^{13}C NMR (100 MHz, CDCl3) δ 160.10, 146.36, 143.32, 129.65, 125.72, 121.15, 114.72, 78.48, 75.75, 63.50, 58.05, 55.39, 50.72.

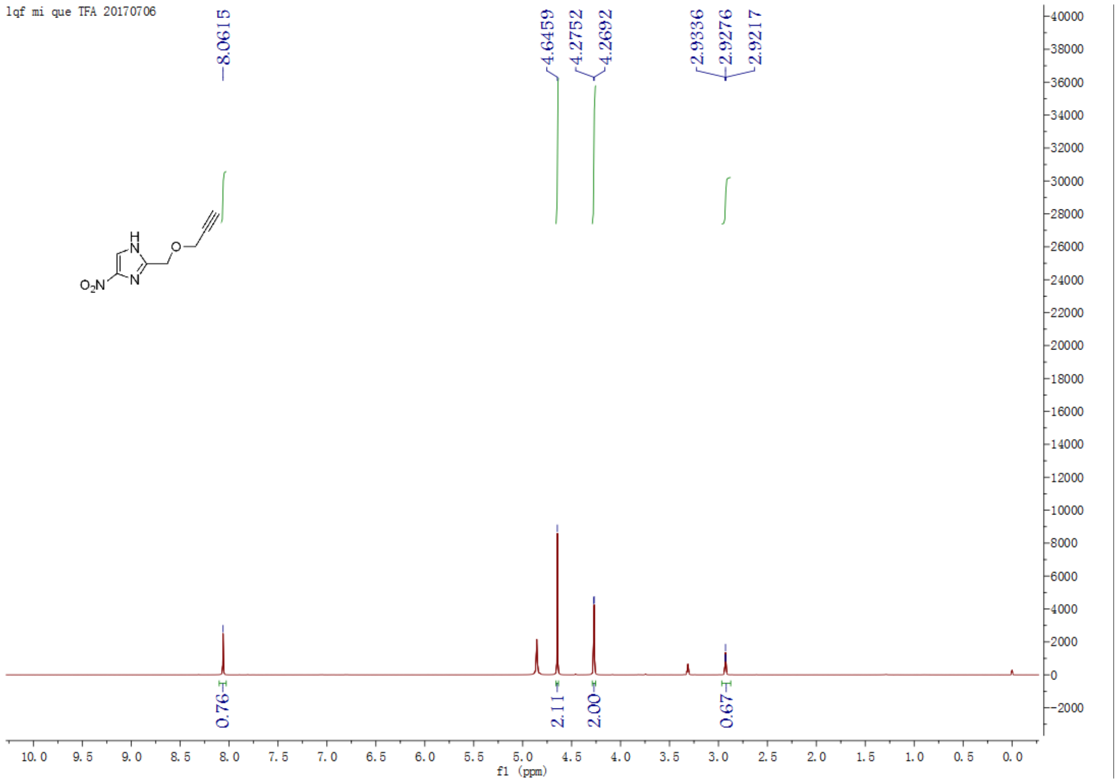

Supplementary Figure 58. ^{1}H NMR spectrum of compound **m6**.

A single-neck flask was charged with compound **m5** (0.078 g, 0.26 mmol), TFA (20 mL) and anisole (2.0 mL). The mixture was stirred vigorously at 80 °C for 12 h before cooled to room temperature. The solution was concentrated, triturated with ethyl ether and filtered to yield compound **m6** as light brown solid (0.038 g, 81% yield).

^{1}H NMR (400 MHz, MeOD) δ 8.06 (s, 1H), 4.65 (s, 2H), 4.27 (d, *J* = 2.4 Hz, 2H), 2.93 (t, *J* = 2.4 Hz, 1H). ^{13}C NMR (100 MHz, CD_3OD) δ 148.42, 146.27, 119.55, 79.66, 76.90, 64.94, 58.98.

HRMS (ESI) m/z calcd. for $C_7H_8N_3O_3$ $[M+H]^+$ 182.0566, found 182.0561.

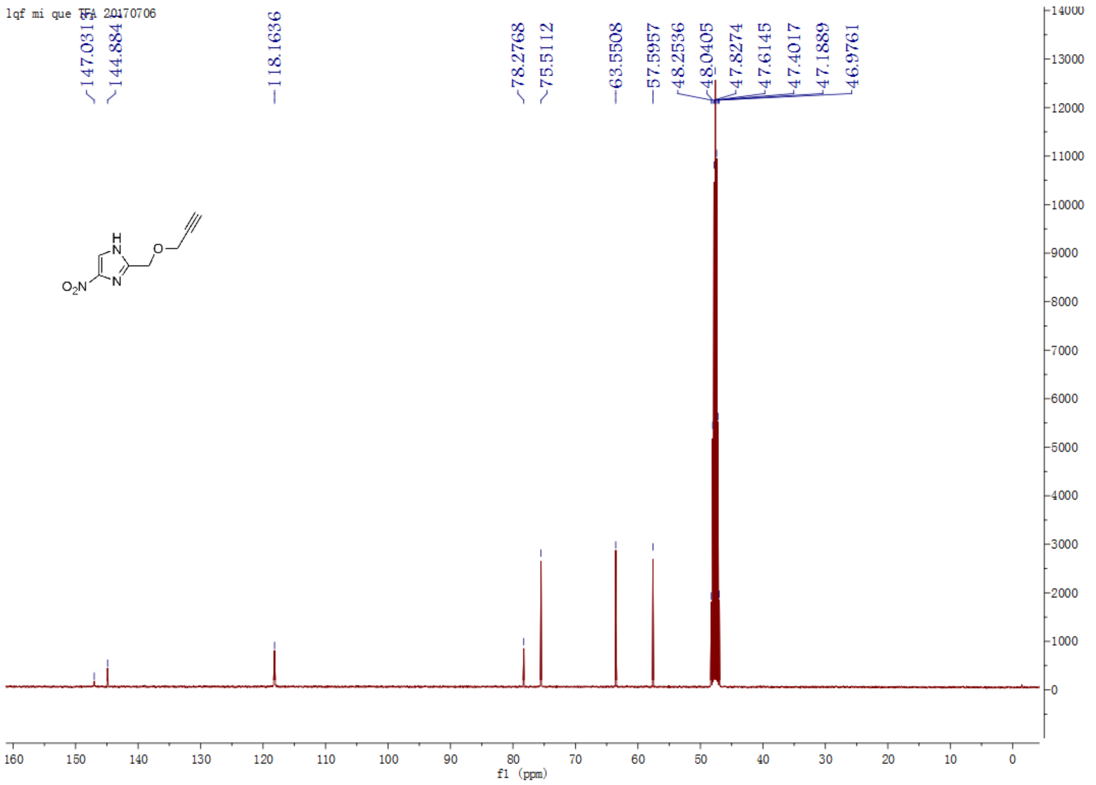

Supplementary Figure 59. ^{13}C NMR spectrum of compound **m6**.

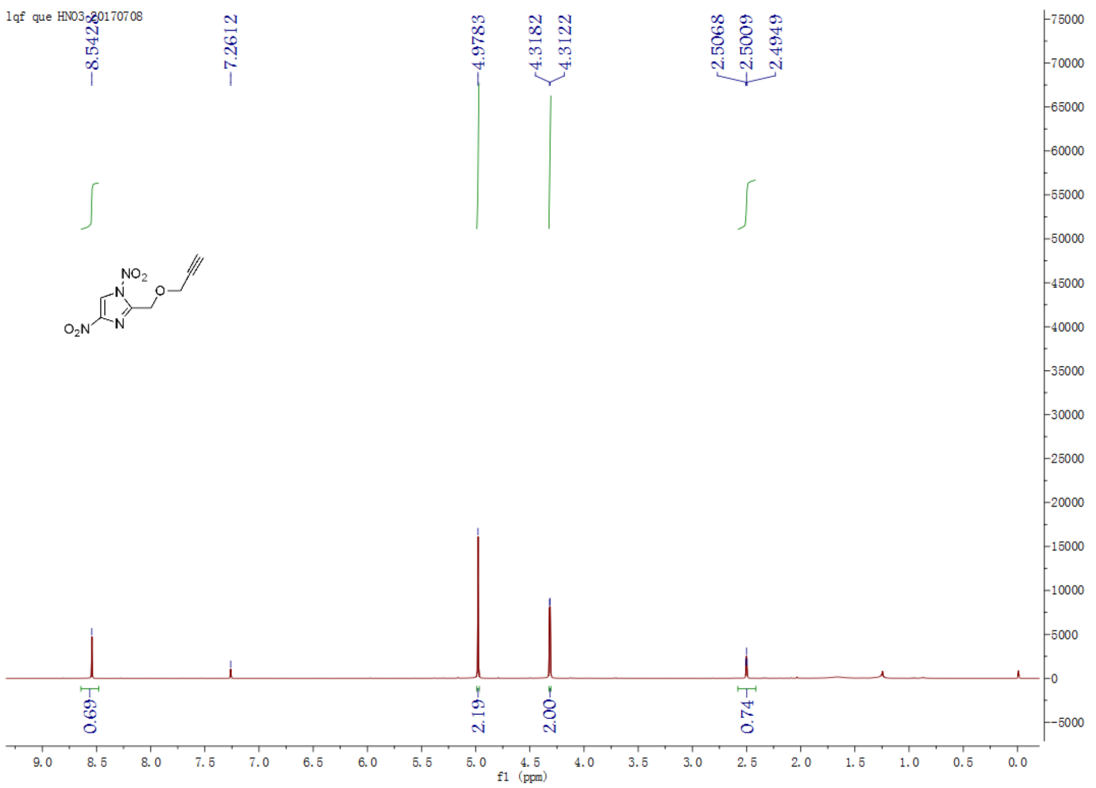

Supplementary Figure 60. ^{1}H NMR spectrum of compound **4c**. To a single-neck flask cooled in ice-salt bath was added successively CH_3COOH (2.8 mL), acetic anhydride (0.12 mL, 1.2 mmol) and fuming HNO_3 (0.04 mL, 0.95 mmol). The mixture was stirred for 1 h at r.t. before compound **m6** (25 mg, 0.14 mmol) was added. The reaction was continued for 1.5 h at r.t. and quenched with the addition of DCM (40 mL). The mixture was washed with brine and the organic layer was dried with anhydrous Na_2SO_4, concentrated and subjected to flash column chromatography (PE/EA=10/3, v/v) to afford compound **4c** as a light brown solid (23 mg, 74% yield).

^{1}H NMR (400 MHz, $CDCl_3$) δ 8.54 (s, 1H), 4.98 (s, 2H), 4.31 (d, J = 2.4 Hz, 2H), 2.50 (t, J = 2.4 Hz, 1H).

^{13}C NMR (100 MHz, $CDCl_3$) δ 140.93, 115.03, 78.04, 75.99, 63.84, 58.67.

HRMS (ESI) m/z calcd. for $C_7H_7N_4O_5$ $[M+H]^+$ 227.0416, found 227.0414.

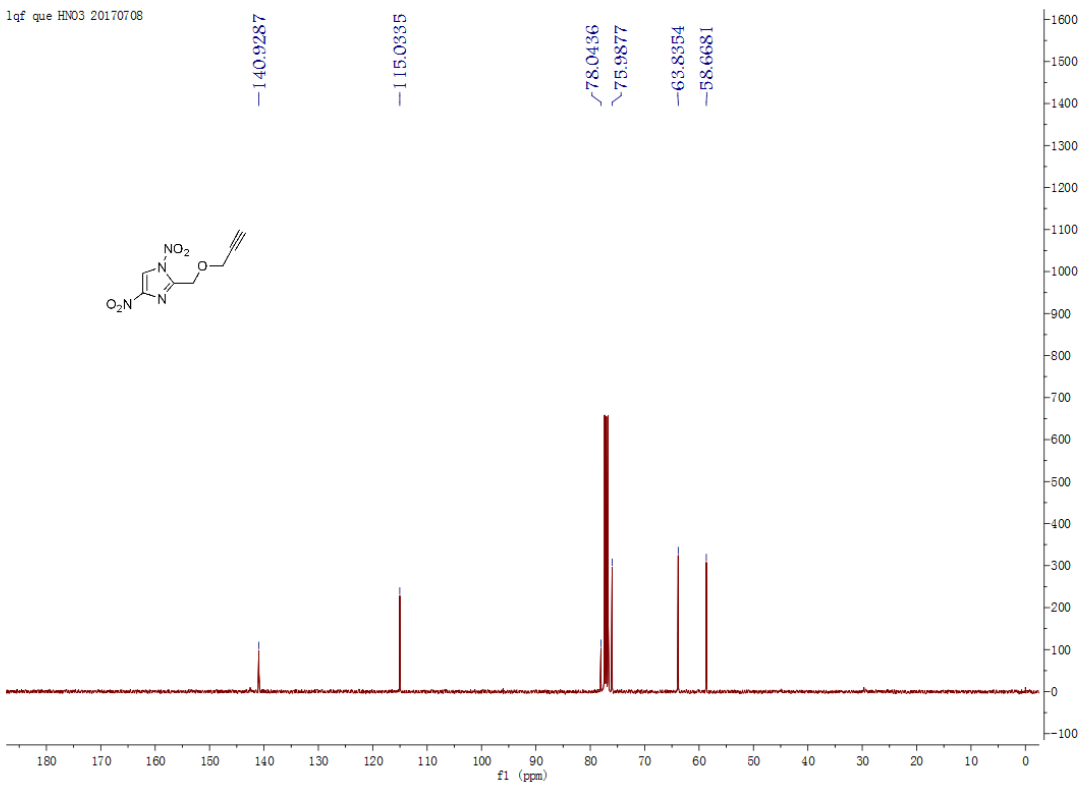

Supplementary Figure 61. ^{13}C NMR spectrum of compound **4c**.

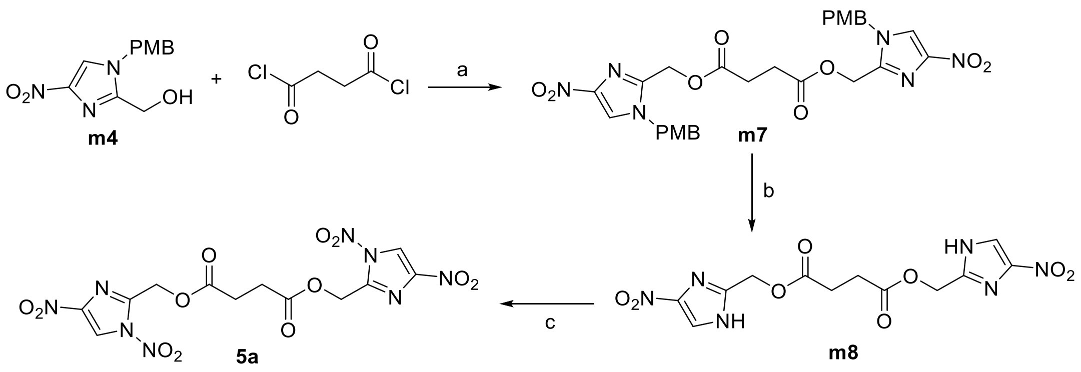

Supplementary Figure 62. Synthesis of stapling agent **5a**. a) Dry DCM, dry TEA, DMAP, N_2, 0 ^oC~r.t., 63% yield; b) TFA, anisole, 80 ^oC, 99% yield; c) Ac_2O, fuming HNO_3, -5 ^oC~r.t., 62% yield.

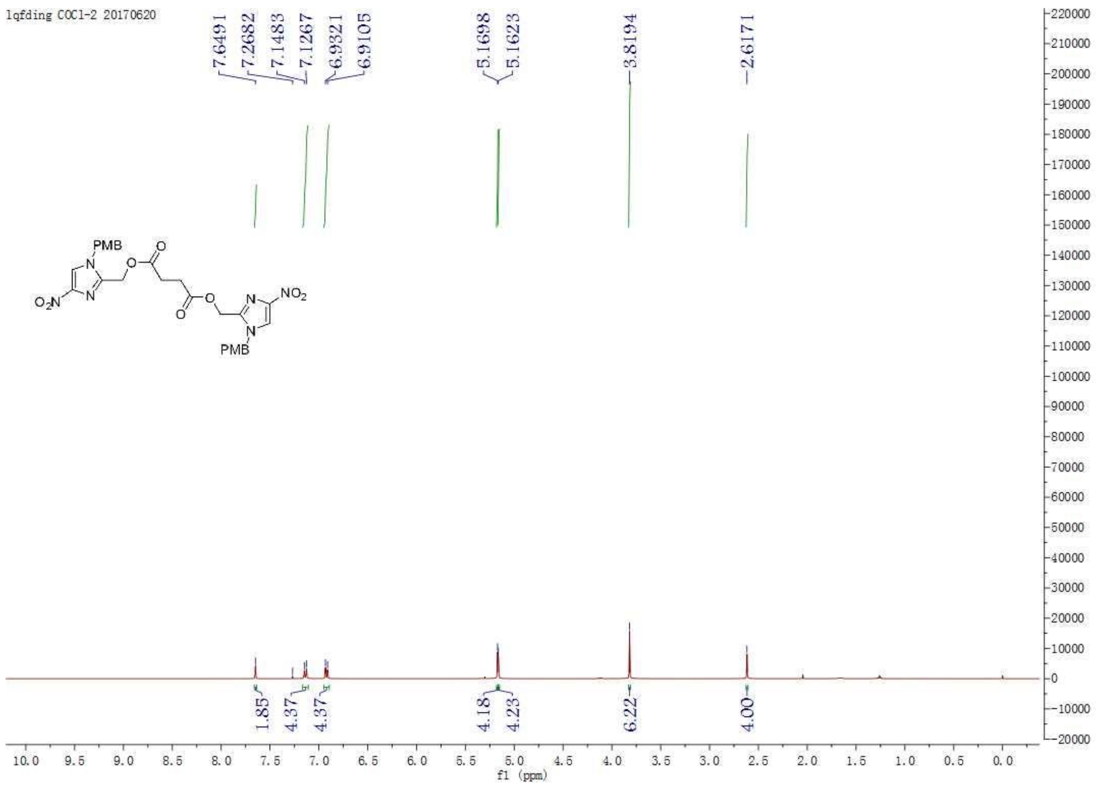

Supplementary Figure 63. ^{1}H NMR spectrum of compound **m7**. To a two-neck flask cooled in ice bath was added compound **m4** (0.13 g, 0.50 mmol) and DMAP (6 mg, 0.05 mmol). Under N_2 atmosphere, to the solution was added dry TEA (0.21 mL, 1.5 mmol) in dry DCM (2.0 mL) in one portion followed by succinic chloride (0.028 mL, 0.25 mmol) in dry DCM (3.0 mL) dropwise. The mixture was stirred overnight at room temperature, diluted with DCM and washed with brine (2×5 ml). The organic layer was

dried, concentrated and purified with flash column chromatography (DCM/MeOH = 70/1, v/v) to yield compound **m7** as a white solid (0.095 g, 63% yield).

^{1}H NMR (400 MHz, $CDCl_3$) δ 7.65 (s, 2 H), 7.15-7.13 (m, 4 H), 6.93- 6.91 (m, 4 H), 5.17 (s, 4 H), 5.16 (s, 4 H), 3.82 (s, 6 H), 2.62 (s, 4 H).

HRMS (ESI) m/z calcd for $C_{28}H_{28}N_6NaO_{10}$ $[M+Na]^+$ 631.1765, found 631.1764.

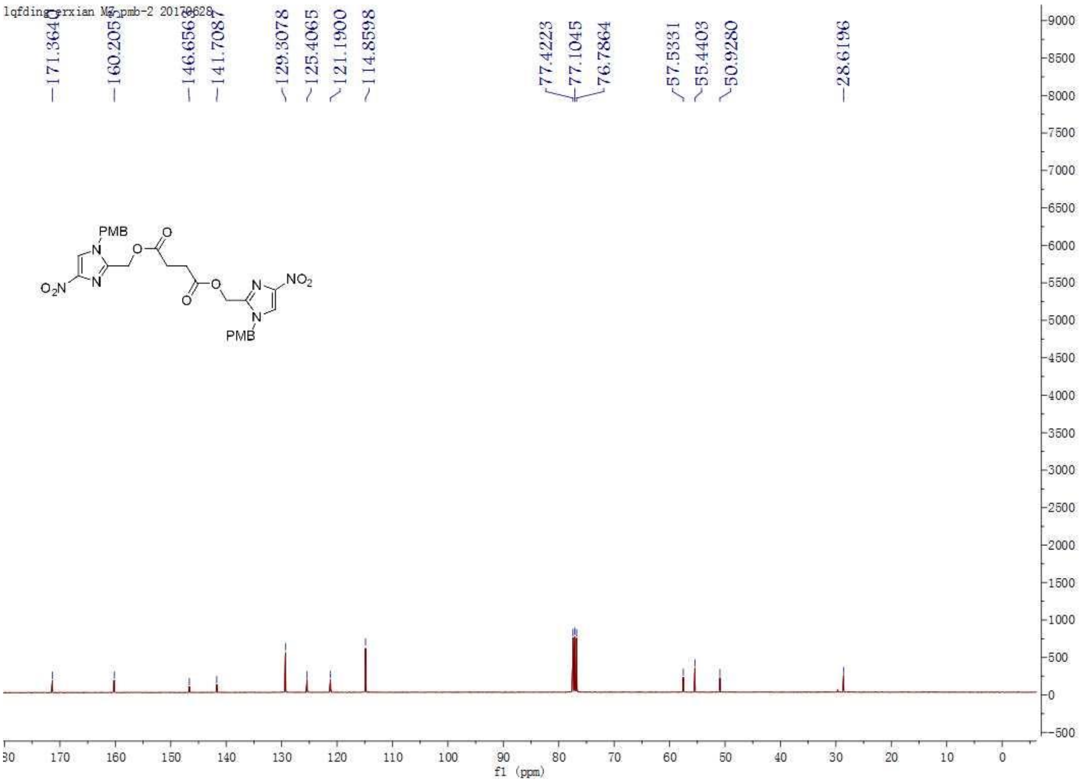

Supplementary Figure 64. ^{13}C NMR spectrum of compound **m7**. ^{13}C NMR (100 MHz, $CDCl_3$) δ 171.36, 160.21, 146.66, 141.71, 129.31, 125.41, 121.19, 114.86, 57.53, 55.44, 50.93, 28.62.

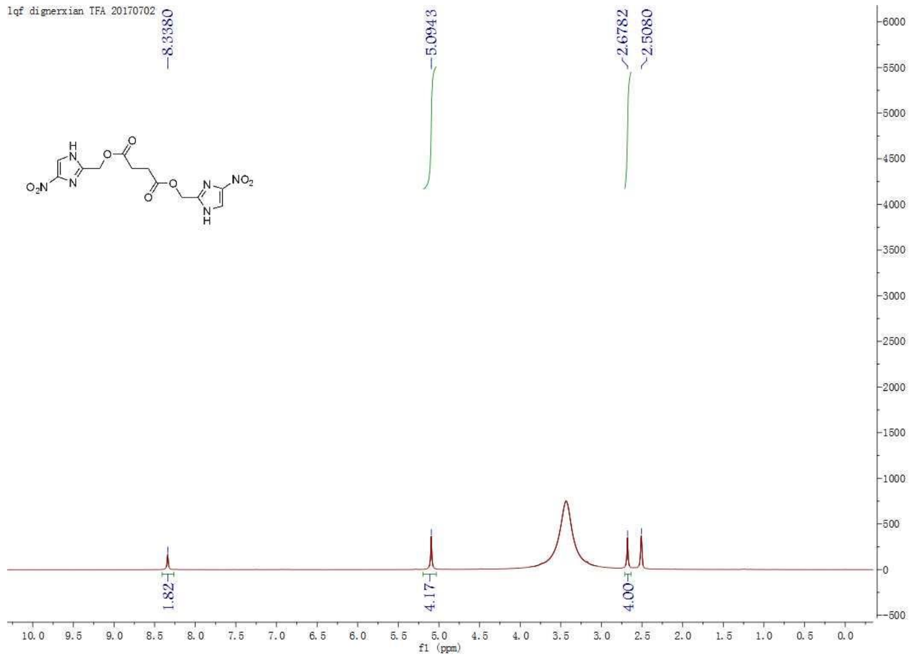

Supplementary Figure 65. ^{1}H NMR spectrum of compound **m8**. A single-neck flask was charged with compound **m7** (0.386 g, 0.635 mmol), TFA (20 mL) and anisole (2.0 mL). The mixture was stirred vigorously at 80 ^{o}C for 12 h before cooled to room temperature. The solution was concentrated, triturated with ethyl ether and filtered to yield **m8** as a light brown solid (0.230 g, 99% yield).

^{1}H NMR (400 MHz, DMSO-d_6) δ 8.34 (s, 2 H), 5.09 (s, 4 H), 2.68 (s, 4 H).

HRMS (ESI) m/z calcd for $C_{12}H_{12}N_6NaO_8$ $[M+Na]^+$ 391.0614, found 391.0611.

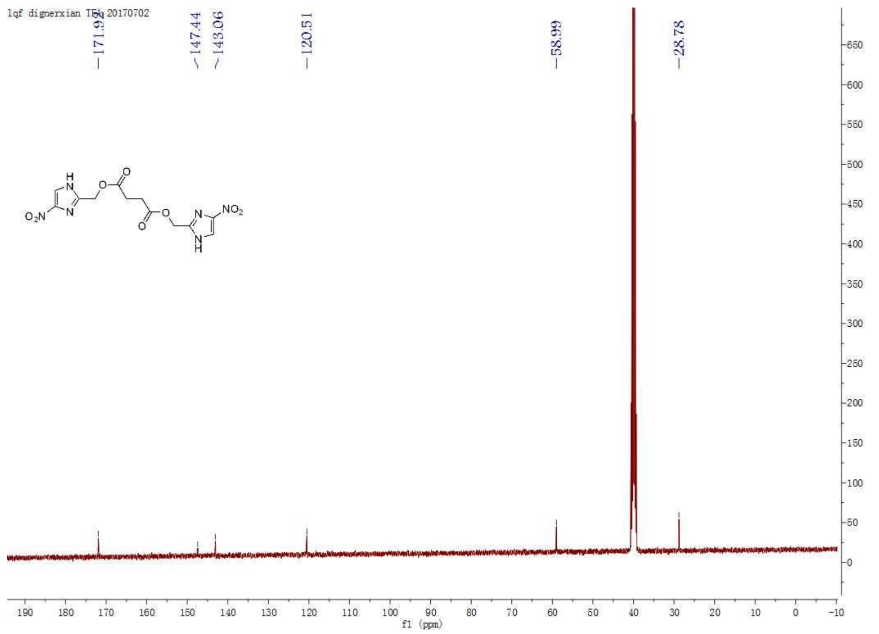

Supplementary Figure 66. ^{13}C NMR spectrum of compound **m8**. ^{13}C NMR (100 MHz, DMSO-d_6) δ 171.92, 147.44, 143.06, 120.51, 58.99, 28.78.

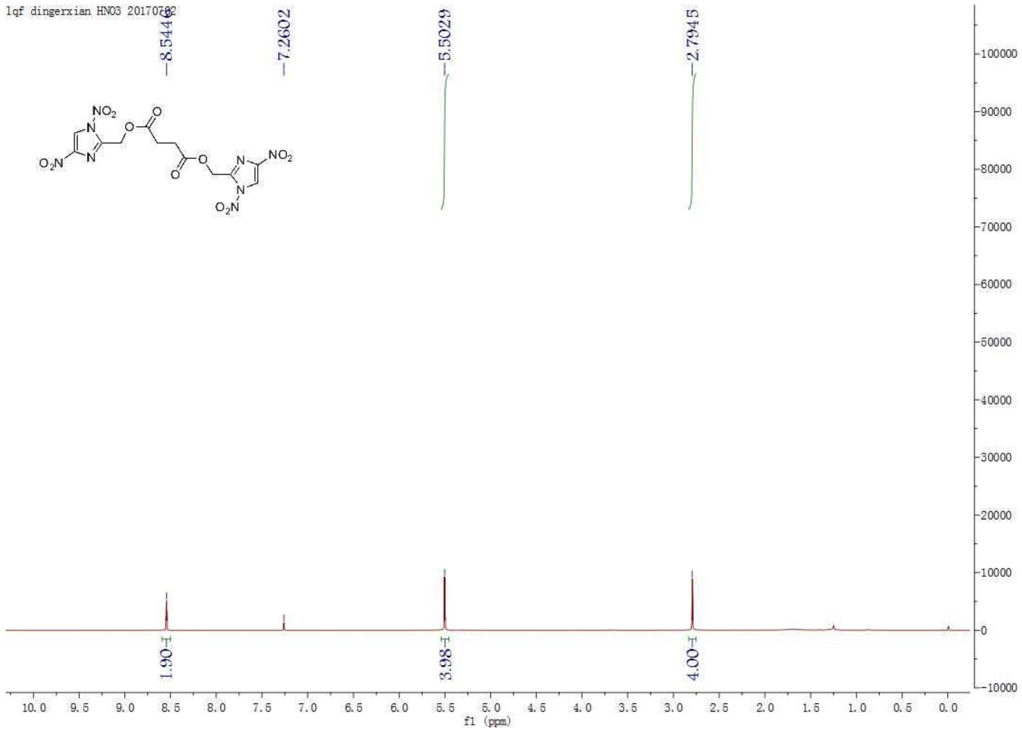

Supplementary Figure 67. ^{1}H NMR spectrum of compound **5a.**

To a single-neck flask cooled in ice bath was added acetic anhydride (0.20 mL, 2.0 mmol) and fuming HNO_3 (0.10 mL, 2.4 mmol). The mixture was stirred for 30 min at 0 ^oC before compound **m8** (0.012 g, 0.033 mmol) was added. The reaction was continued for additional one hour, quenched with the addition of DCM. The mixture was washed with brine and the organic layer was dried with anhydrous Na_2SO_4, concentrated and subjected to flash column chromatography (DCM/MeOH=40/1, v/v) to afford compound **5a** as a light green solid (9.0 mg, 62% yield).

^{1}H NMR (400 MHz, $CDCl_3$) δ 8.54(s, 2 H), 5.50 (s, 4 H), 2.79 (s, 4 H).

HRMS (ESI) m/z calcd. for $C_{12}H_{10}N_8NaO_{12}$ $[M+Na]^+$ 481.0316, found 481.0310.

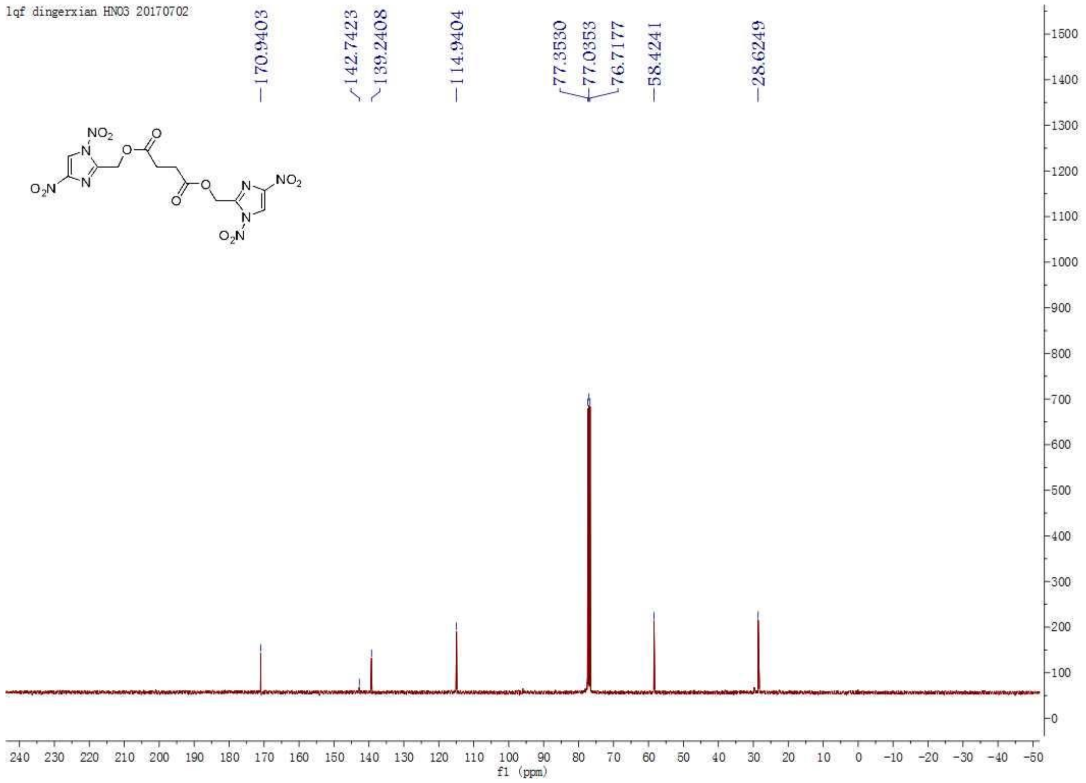

Supplementary Figure 68. ^{13}C NMR spectrum of compound **5a.** ^{13}C NMR (100 MHz, $CDCl_3$) δ 170.94, 142.74, 139.24, 114.94, 58.42, 28.62.

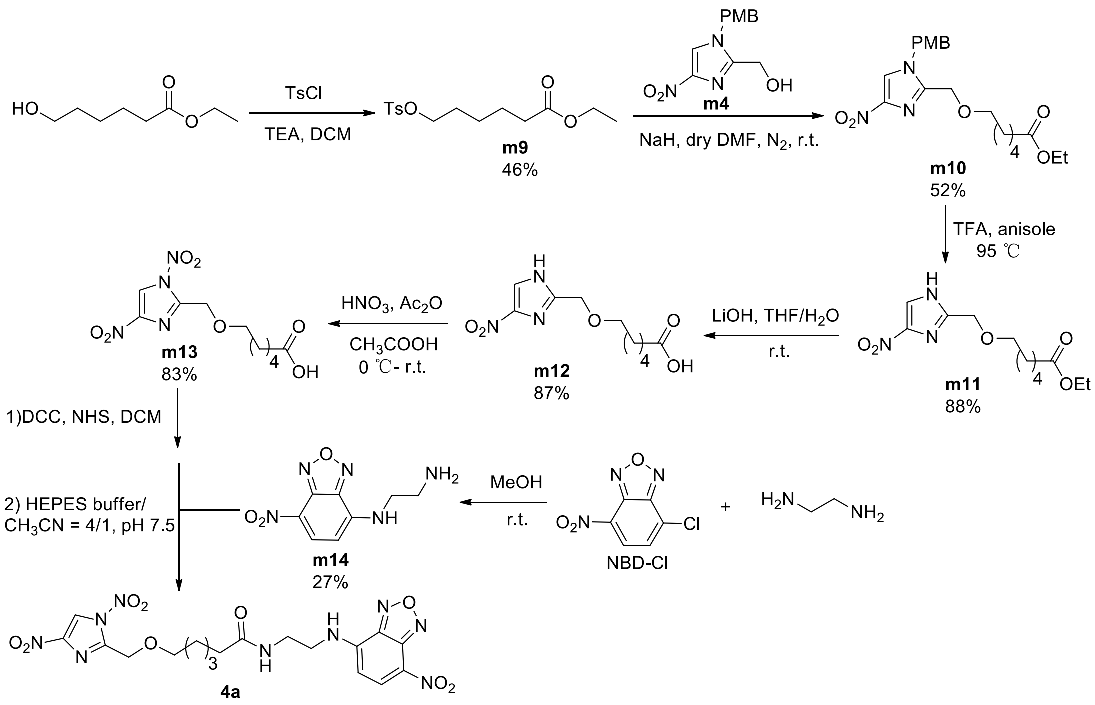

Supplementary Figure 69. Synthesis of compound **4a**.

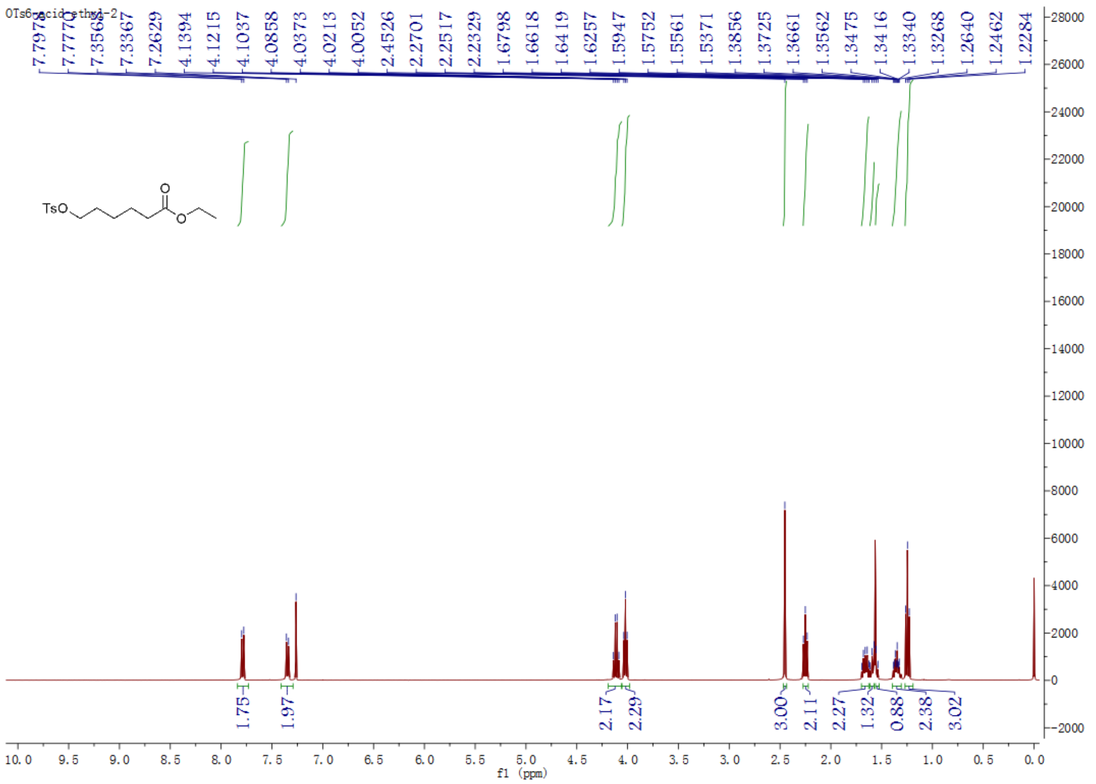

Supplementary Figure 70. ^{1}H NMR spectrum of compound **m9**. To a single-neck flask in ice bath was added compound ethyl 6-hydroxyhexanoate (800 mg, 5.0 mmol), TEA (1.1 mL, 7.5 mmol) and DCM (10.0 mL).The mixture was stirred vigorously followed by the careful addition of TsCl (1143 mg, 6.0 mmol) in DCM (10.0 mL) . Then the reaction was proceeded at r.t. for 5 h. The mixture was concentrated and purified with flash silica gel column chromatography (PE/EA=10/1, v/v) to yield **m9** as clear oil (710 mg, 46% yield).

^{1}H NMR (400 MHz, $CDCl_3$) δ 7.79 (d, *J* = 8.3 Hz, 2H), 7.35 (d, *J* = 8.1 Hz, 2H), 4.11 (q, *J* = 7.1 Hz, 2H), 4.02 (t, *J* = 6.4 Hz, 2H), 2.45 (s, 3H), 2.25 (t, *J* = 7.4 Hz, 2H), 1.70 – 1.61 (m, 2H), 1.59 – 1.55 (m, 2H), 1.39 – 1.33 (m, 2H), 1.25 (t, *J* = 7.1 Hz, 3H).

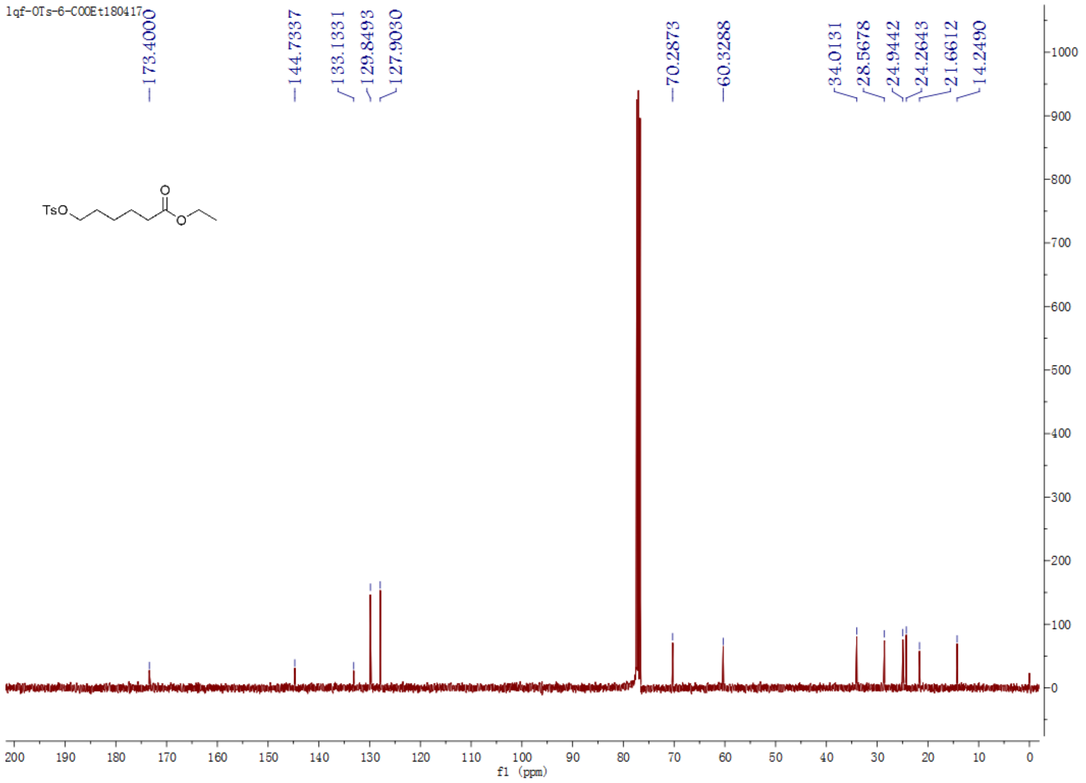

Supplementary Figure 71. ^{13}C NMR spectrum of compound **m9**. ^{13}C NMR (100 MHz, $CDCl_3$) δ 173.40, 144.73, 133.13, 129.85, 127.90, 70.29, 60.33, 34.01, 28.57, 24.94, 24.26, 21.66, 14.25.

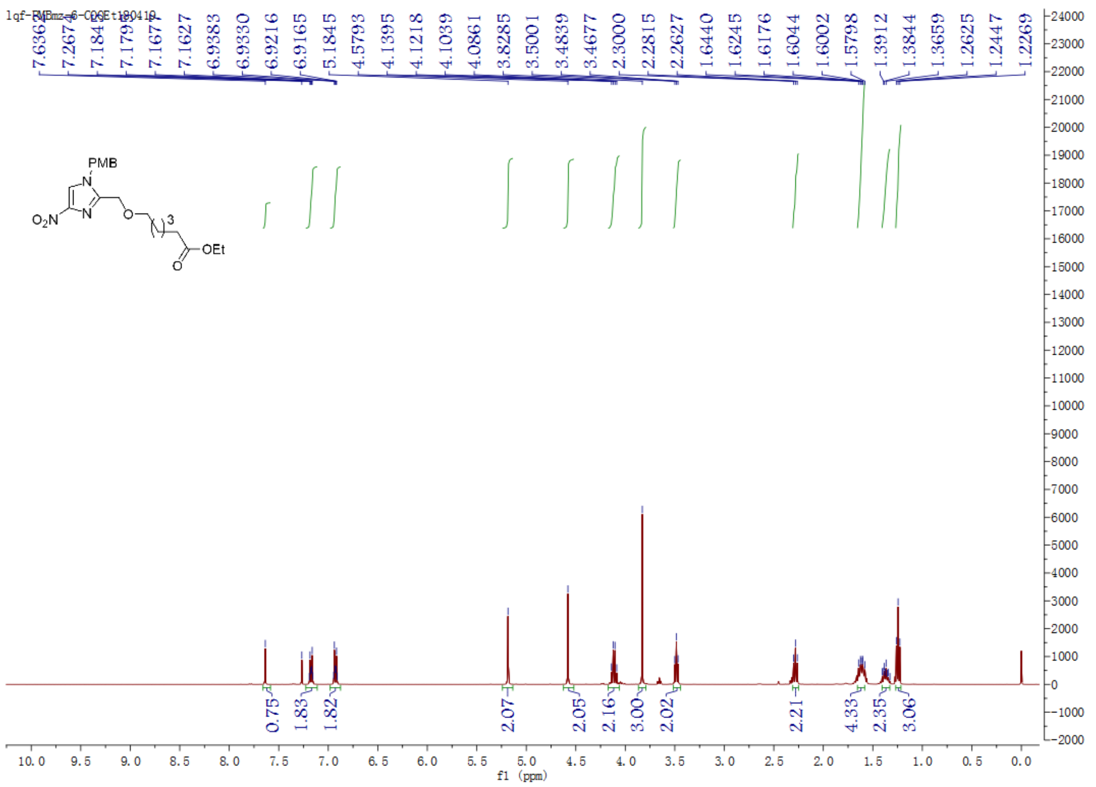

Supplementary Figure 72. ^{1}H NMR spectrum of compound **m10**. To a two-neck flask was added compound **m4** (130 mg, 0.50 mmol) and NaH (18 mg, 0.75 mmol). Then under N_2 atmosphere dry DMF (1 mL) and compound **m9** (235 mg, 0.75 mmol) in DMF (1 mL) was added successively through a syringe. The reaction was continued for 22 h, diluted with DCM (50 mL) and neutralized with saturated NH_4Cl solution. The aqueous layer was extracted with DCM (20 mL×2)。The organic layer was combined, washed with brine (5mL×2) and dried with anhydrous Na_2SO_4. The organic layer was filtered and purified with flash silica gel column chromatography (PE/EA=10/7, v/v) to yield **m10** as green yellow oil (105 mg, 52% yield).

^{1}H NMR (400 MHz, $CDCl_3$) δ 7.64 (s, 1H), 7.23 – 7.11 (m, 2H), 6.98 – 6.88 (m, 2H), 5.18 (s, 2H), 4.58 (s, 2H), 4.11 (q, J = 7.1 Hz, 2H), 3.83 (s, 3H), 3.48 (t, J = 6.5 Hz, 2H), 2.28 (t, J = 7.5 Hz, 2H), 1.66 – 1.58 (m, 4H), 1.41 – 1.33 (m, 2H), 1.24 (t, J = 7.1 Hz, 3H).

HRMS (ESI) m/z calcd. for $C_{20}H_{27}N_3NaO_6$ $[M+Na]^+$ 428.1798, found 428.1799.

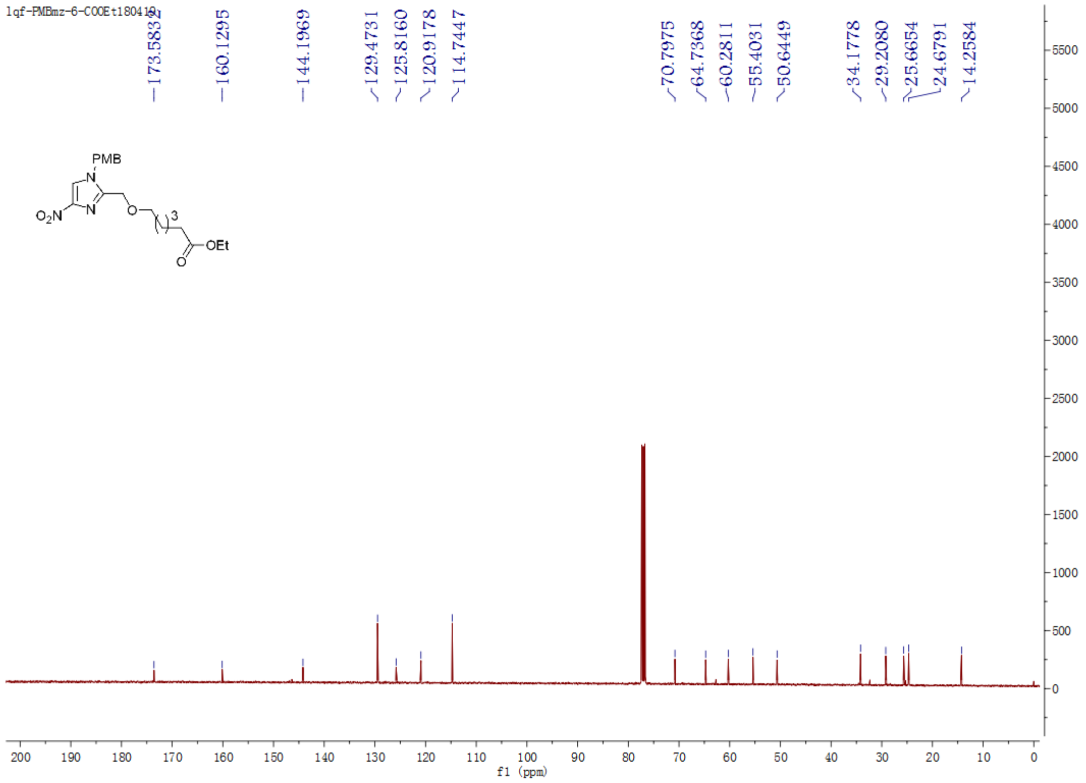

Supplementary Figure 73. ^{13}C NMR spectrum of compound **m10**. ^{13}C NMR (100 MHz, $CDCl_3$) δ 173.58, 160.13, 144.20, 129.47, 125.82, 120.92, 114.74, 70.80, 64.74, 60.28, 55.40, 50.64, 34.18, 29.21, 25.67, 24.68, 14.26.

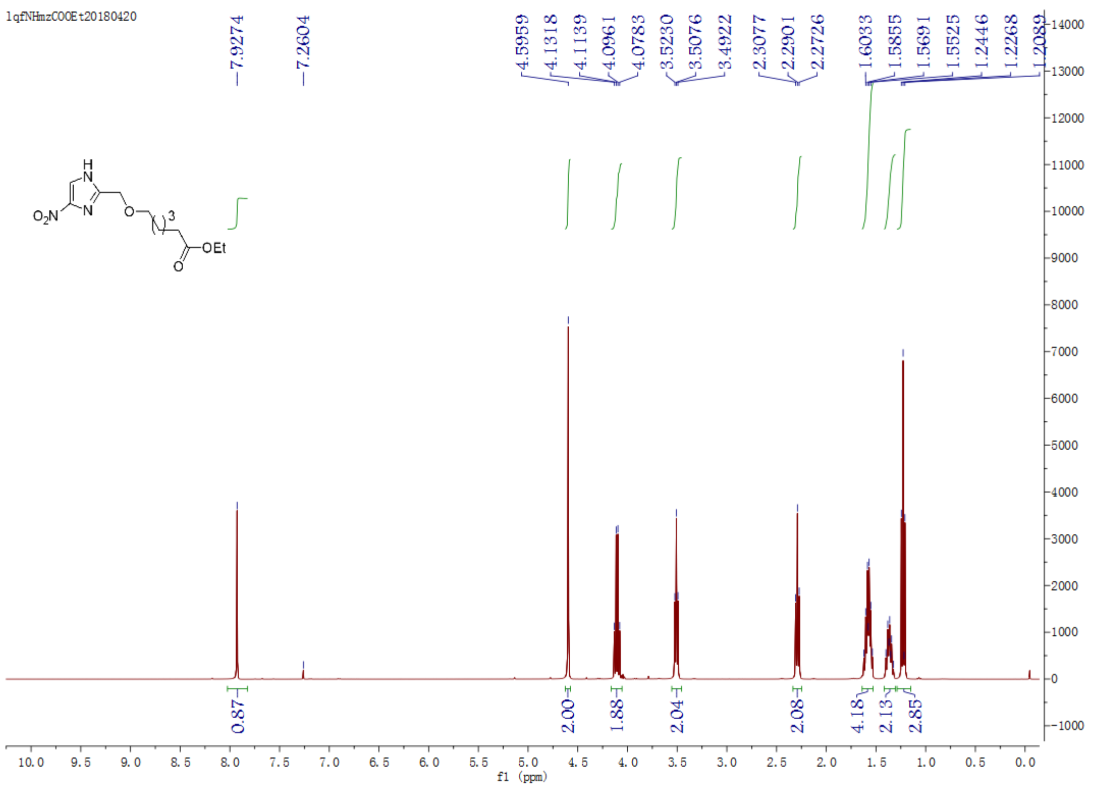

Supplementary Figure 74. ^{1}H NMR spectrum of compound **m11**. To a single-neck flask was added successively compound **m10** (107 mg, 0.26 mmol), TFA (5.0 mL) and anisole (0.5 mL). The reaction was proceeded at 95°C for 2 h until TLC showed the completion. The mixture was concentrated and purified with flash silica gel column chromatography (PE/EA/MeOH=10/5/0.2, v/v/v) to yield **m11** as reddish-brown oil (66 mg, 88% yield).

^{1}H NMR (400 MHz, $CDCl_3$) δ 7.93 (s, 1H), 4.60 (s, 2H), 4.11 (q, J = 7.1 Hz, 2H), 3.51 (t, J = 6.2 Hz, 2H), 2.29 (t, J = 7.0 Hz, 2H), 1.64 – 1.53 (m, 4H), 1.42 – 1.30 (m, 2H), 1.23 (t, J = 7.1 Hz, 3H).

HRMS (ESI) m/z calcd. for $C_{12}H_{19}N_3NaO_5$ $[M+Na]^+$ 308.1222, found 308.1225.

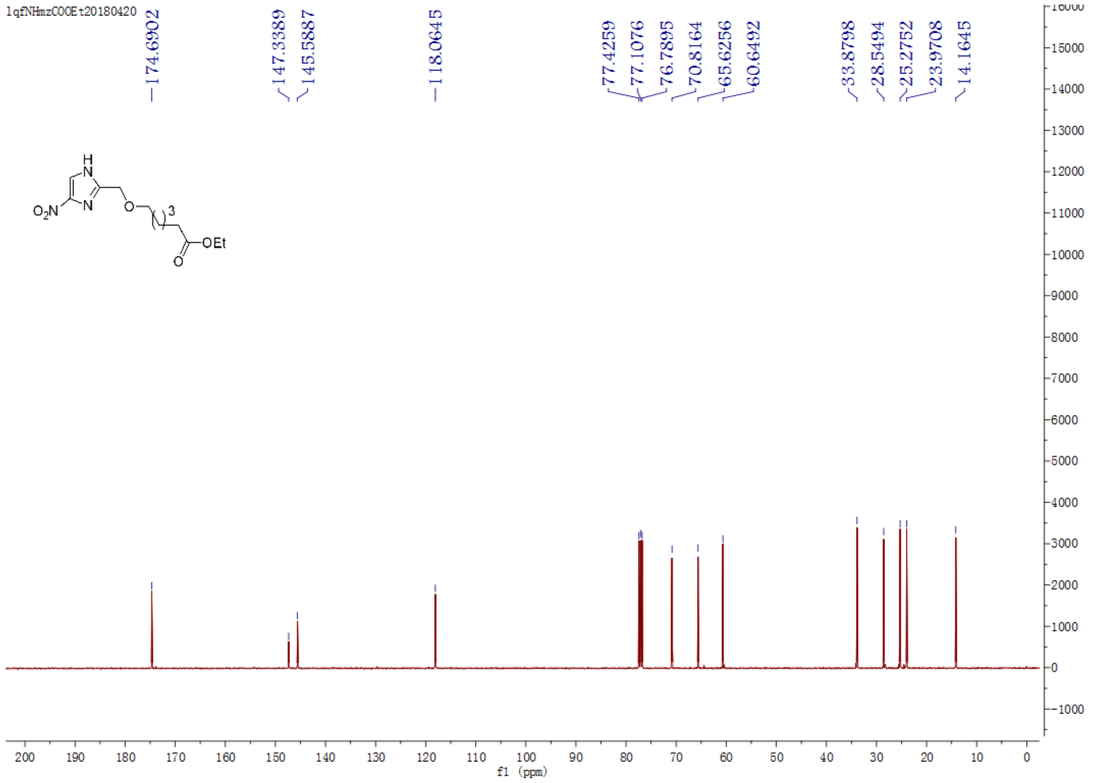

Supplementary Figure 75. ^{13}C NMR spectrum of compound **m11**. ^{13}C NMR (100 MHz, $CDCl_3$) δ 174.69, 147.34, 145.59, 118.06, 77.43, 77.11, 76.79, 70.82, 65.63, 60.65, 33.88, 28.55, 25.28, 23.97, 14.16.

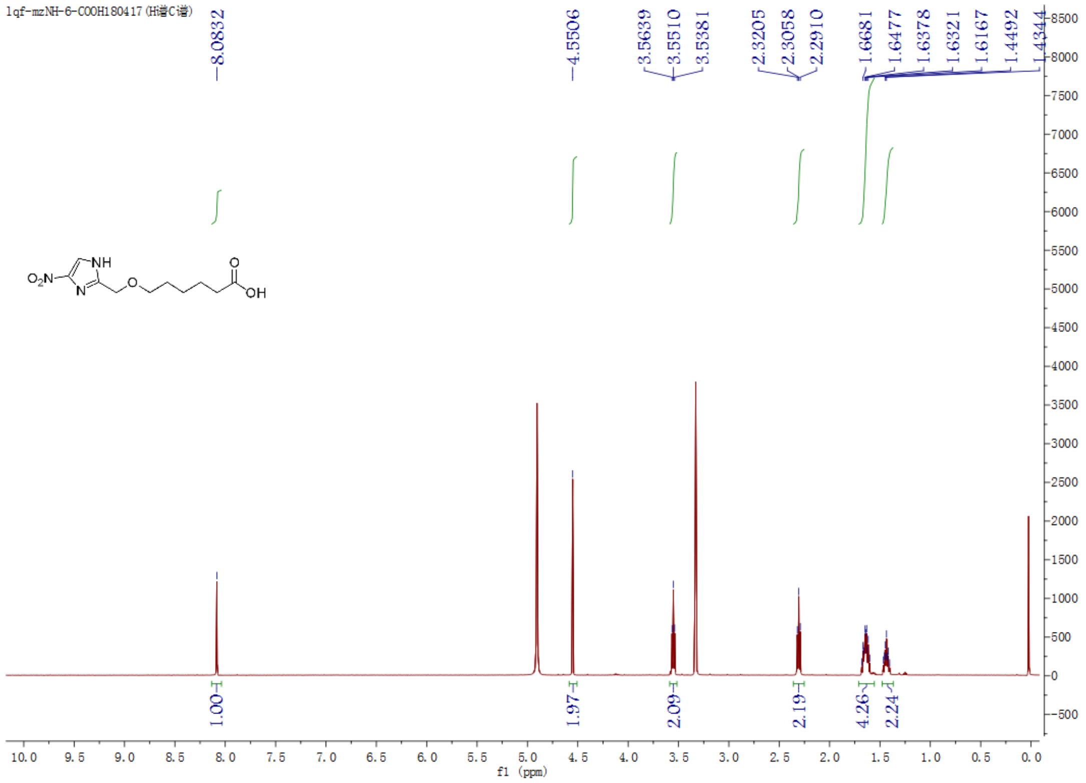

Supplementary Figure 76. ^{1}H NMR spectrum of compound **m12**. To a single-neck flask was added successively compound **m11** (66 mg, 0.23 mmol), THF (1.0 mL), water (1 mL) and $LiOH{\cdot}H_2O$ (20 mg, 0.46 mmol). The mixture was stirred at r.t. for 2 h then neutralized with diluted $NaHCO_3$ solution. The aqueous layer was washed with ether (8 mL×1) and acidified with formic acid, followed by extraction with (15×3 mL). The organic layer was combined, dried with anhydrous Na_2SO_4, filtered, concentrated and dried in vacuum to yield **m12** as a white solid (52 mg, 87% yield).

^{1}H NMR (500 MHz, MeOD) δ 8.08 (s, 1H), 4.55 (s, 2H), 3.55 (t, *J* = 6.4 Hz, 2H), 2.31 (t, *J* = 7.4 Hz, 2H), 1.71 – 1.56 (m, 4H), 1.48 – 1.37 (m, 2H).

HRMS (ESI) m/z calcd. for $C_{10}H_{15}N_3NaO_5$ $[M+Na]^+$ 280.0909, found 280.0906.

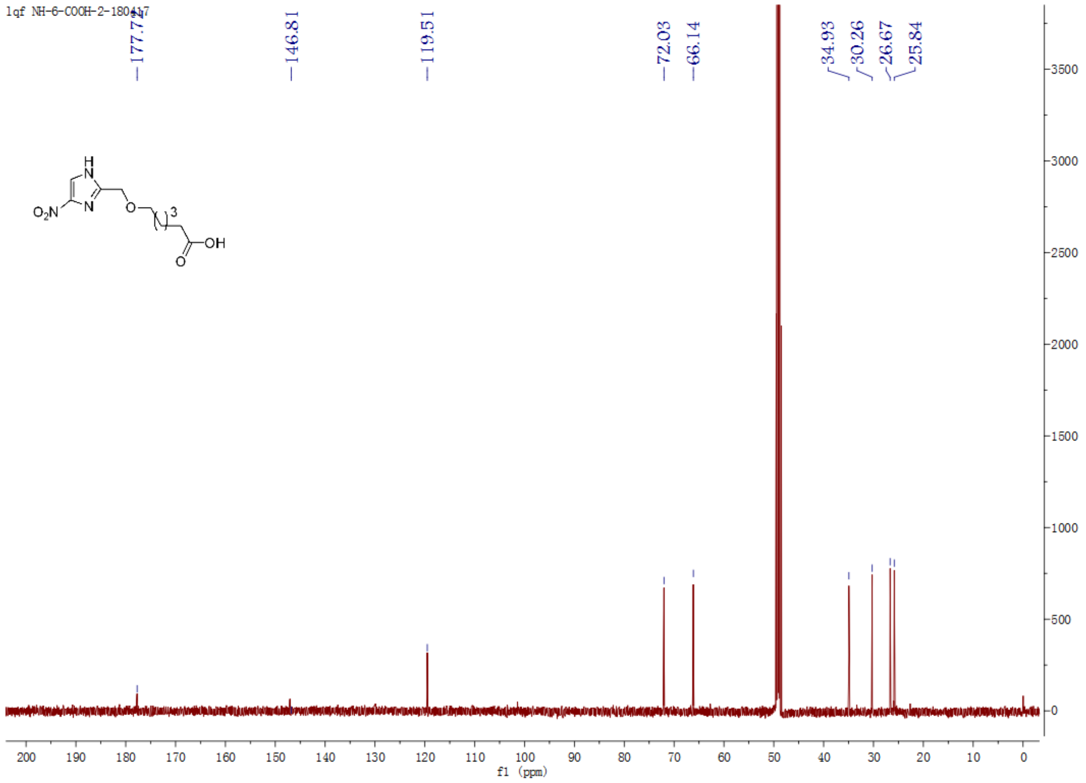

Supplementary Figure 77. ^{13}C NMR spectrum of compound **m12**. ^{13}C NMR (125 MHz, MeOD) δ 177.72, 146.81, 119.51, 72.03, 66.14, 34.93, 30.26, 26.67, 25.84.

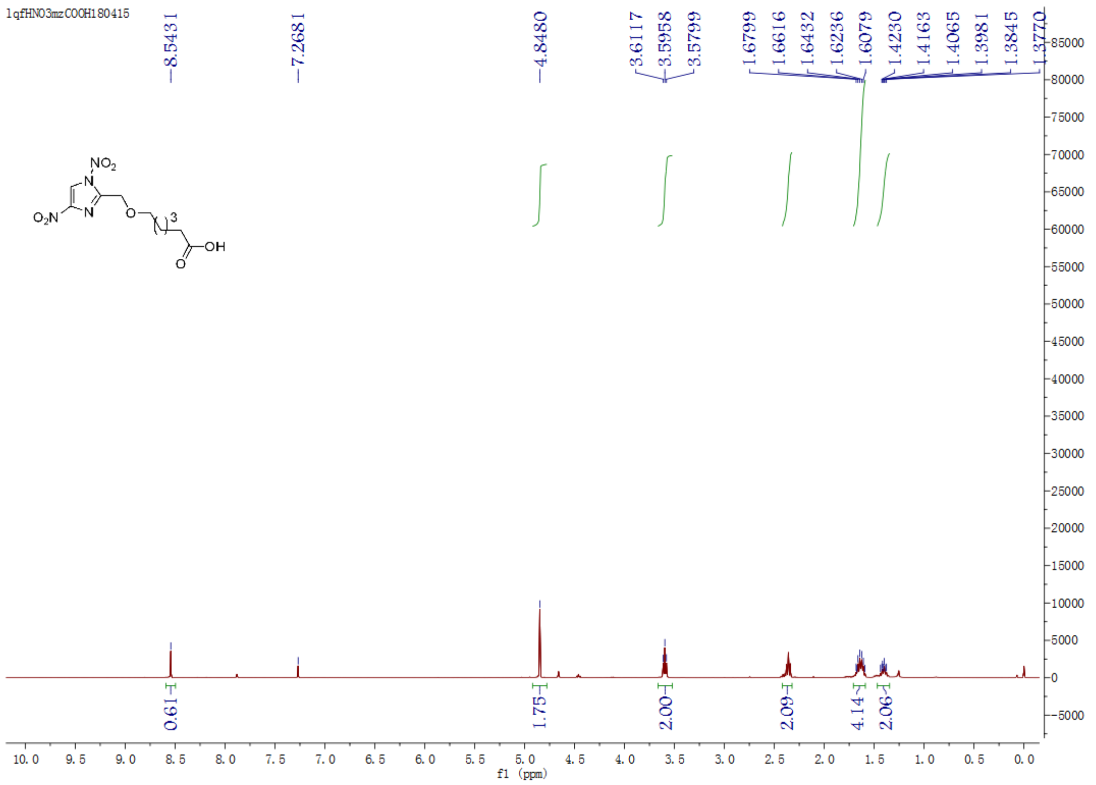

Supplementary Figure 78. ^{1}H NMR spectrum of compound **m13**. To a single-neck flask in ice bath was added CH_3COOH (2 mL), $(CH3CO)_2O$ (130 μL, 1.33 mmol) and HNO_3 (50 μL, 1.10 mmol). The mixture was stirred at r.t. for 2 h, followed by the addition of compound **m12** (30 mg, 0.12 mmol) under 0°C. Then it was transfer to r.t. and stirred for 8 h until the completion of the starting material. The solution was extracted with DCM (20 mL×3) and the extraction was dried with anhydrous Na_2SO_4, filtered, concentrated and dried in vacuum to yield **m13** as a light yellow solid (30 mg, 83% yield).

^{1}H NMR (400 MHz, $CDCl_3$) δ 8.54 (s, 1H), 4.85 (s, 2H), 3.60 (t, J = 6.4 Hz, 2H), 2.36 (t, J = 7.4 Hz, 2H), 1.68 – 1.59 (m, 4H), 1.44 – 1.37 (m, 2H).

HRMS (ESI) m/z calcd. for $C_{10}H_{14}N_4NaO_7$ $[M+Na]^+$ 325.0760, found 325.0744.

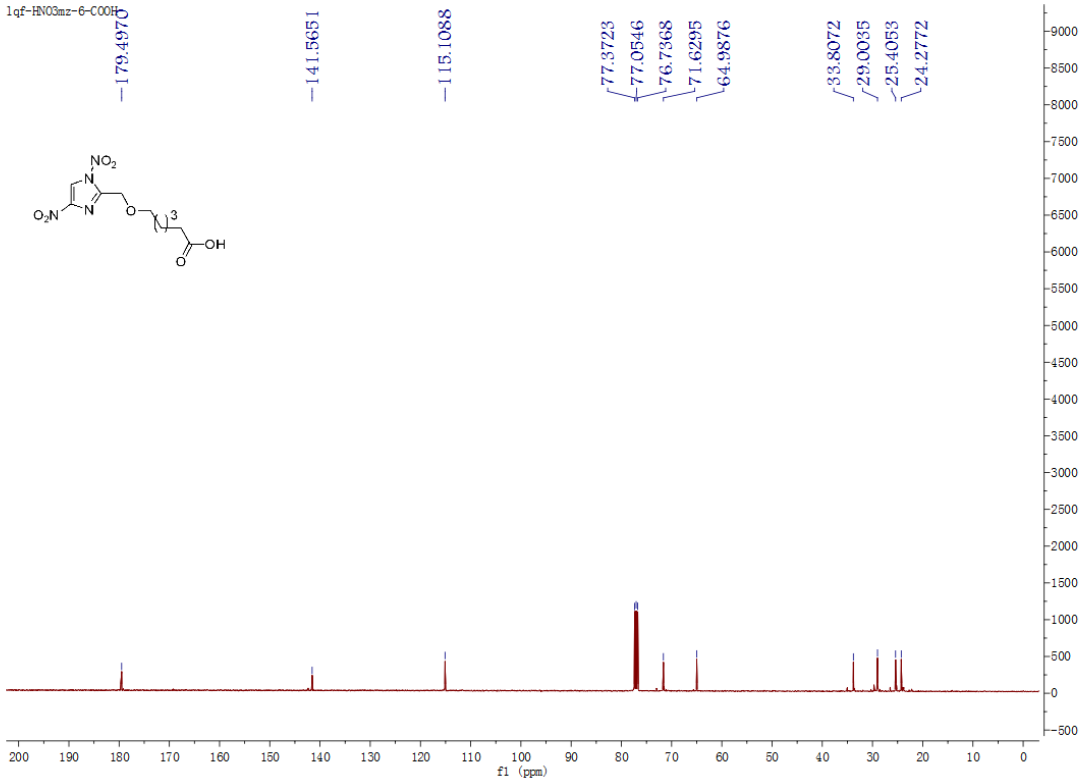

Supplementary Figure 79. ^{13}C NMR spectrum of compound **m13**. ^{13}C NMR (100 MHz, $CDCl_3$) δ 179.50, 141.57, 115.11, 71.63, 64.99, 33.81, 29.00, 25.41, 24.28.

-

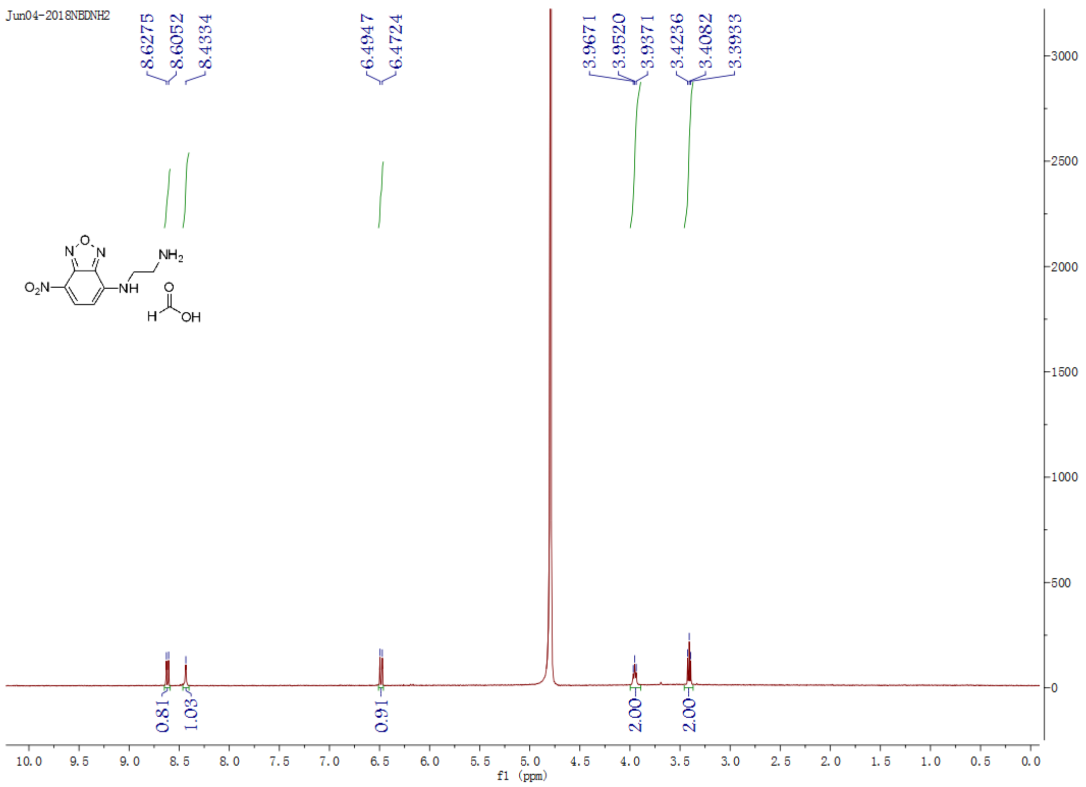

Supplementary Figure 80. ^{1}H NMR spectrum of compound **m14**. To a single-neck flask was added successively compound 4-chloro-7-nitrobenzo[c][1,2,5]oxadiazole **(**NBD-Cl, 200 mg, 1.0 mmol), MeOH (20 mL) and ethane-1,2-diamine (1.3 mL, 20.0 mmol). The mixture was stirred at r.t. overnight, concentrated in vacuum and subjected to preparative HPLC to yield **m14** as a yellow green solid as formic acid salt (72 mg, 27% yield). ^{1}H NMR (400 MHz, D_2O) δ 8.62 (d, *J* = 8.9 Hz, 1H), 6.48 (d, *J* = 8.9 Hz, 1H), 3.95 (t, *J* = 6.0 Hz, 2H), 3.41 (t, *J* = 6.1 Hz, 2H). The peak at 8.43 ppm was assigned to the proton of aldehyde group of formic acid.

Synthesis of compound 6-((1,4-dinitro-1H-imidazol-2-yl)methoxy)-N- (2-((7-nitrobenzo[c][1,2,5]oxadiazol-4-yl)amino)ethyl)hexanamide (4a): To a single-neck flask was added successively compound **m13** (3.6 mg, 0.012 mmol), DCC (3.0 mg, 0.012 mmol), NHS (1.4 mg, 0.012 mmol) and DCM (2 mL). The mixture was stirred at r.t. for 2 h before concentrated in vacuum. The resulting solid was redissolved in CH_3CN(0.5 mL) and HEPES buffer (2 mL, pH 7.5, approximately 160 mM final concentration). To the solution was added compound **m14** (2.2 mg, 0.01 mmol). The reaction was stirred at r.t. for 0.5 h before extracted with EA (40 mL) and washed with brine (5 mL×1). The organic layer was dried with anhydrous Na_2SO_4, filtered, concentrated and subjected to preparative TLC to afford **4a** as a brown green solid (1.6 mg, 32% yield).

HRMS (ESI) m/z calcd. for $C_{18}H_{21}N_9NaO_9$ $[M+Na]^+$ 530.1360, found 530.1363.

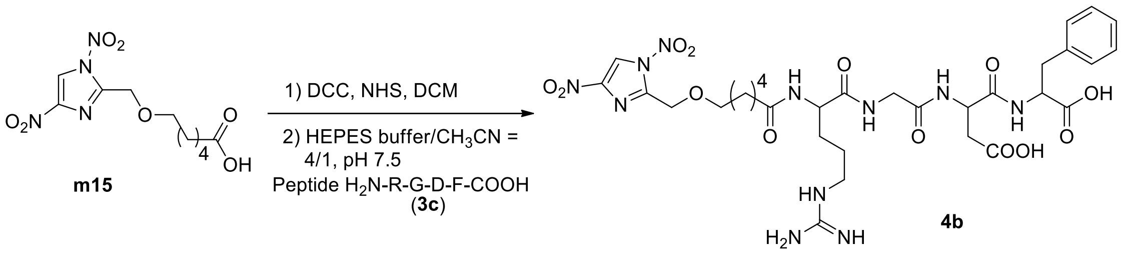

Supplementary Figure 81. Synthesis of compound **4b**. Refer to the procedure for preparing **4a**. White solid (1.5 mg, 20% yield). HRMS (ESI) m/z calcd. for $C_{31}H_{43}N_{11}NaO_{13}$ $[M+Na]^+$ 800.2940, found 800.2947

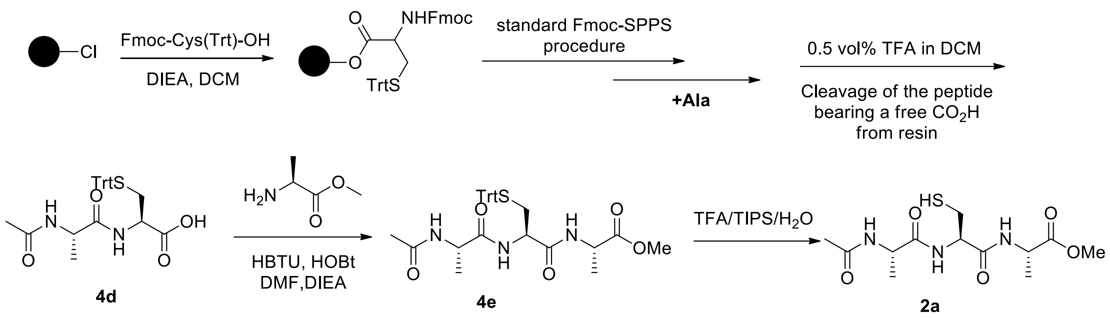

Supplementary Figure 82. Preparation of peptide **2a** by hybrid solid/solution phase synthesis. Peptide **4d** was prepared following the general Fmoc-SPPS procedure starting from 2-chlorotrityl resin (0.5 mmol). After being cleaved from the resin, the crude **4d** was coupled with *L*-Ala-OMe under the general coupling condition in DMF to give compound **4e** as white solid (90.0 mg). Then peptide **4e** was treated with TFA/TIPS/H_2O at r.t. for 3 h and purified by preparative HPLC to obtain peptide **2a** (white solid, 28.0 mg, 18% yield overall). Peptide **2a** was identified by ESI-HRMS. HRMS (ESI) m/z calcd. for $C_{12}H_{21}N_3O_5SNa$ $[M+Na]^+$ 342.1100, found 342.1101.

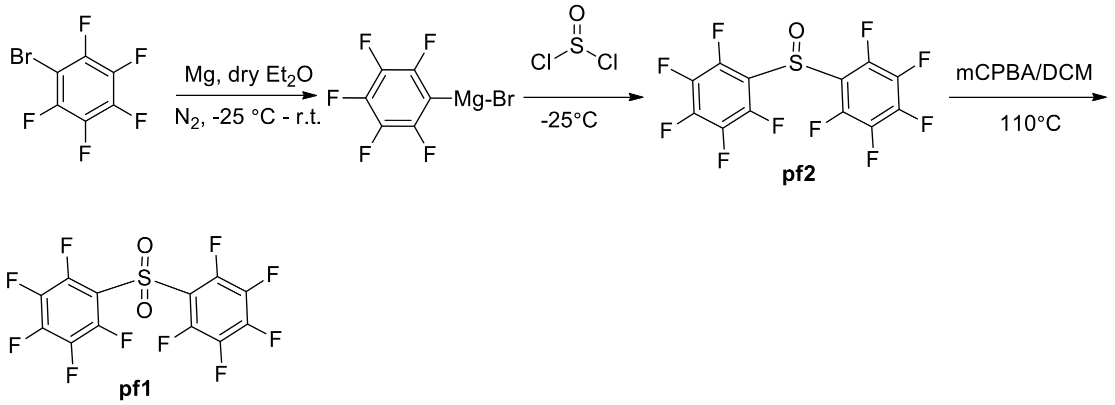

Supplementary Figure 83. Synthesis of compound **pf1**.

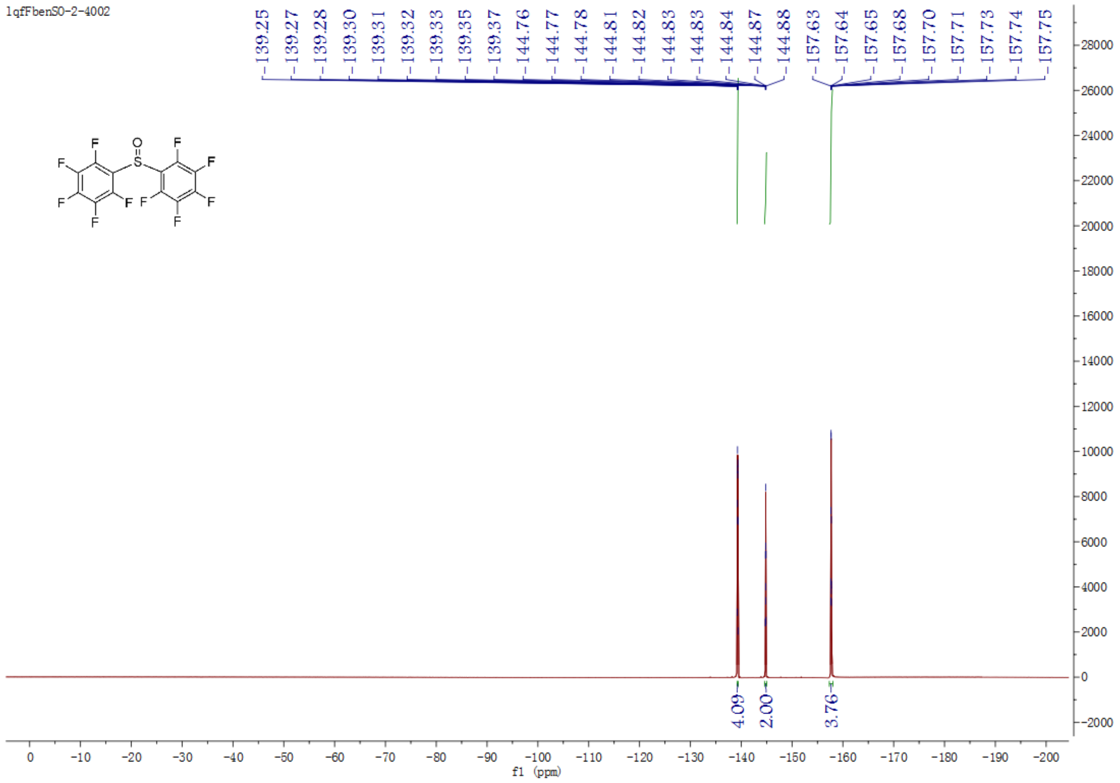

Supplementary Figure 84. ^{19}F NMR spectrum of compound **pf2**.

Synthesis of compound 6,6'-sulfinylbis(1,2,3,4,5-pentafluorobenzene) (pf2). Under -25˚C and N_2 atmosphere, bromopentafluorobenzene (0.62 mL, 5.0 mmol) was added dropwise to a suspension of magnesium turnings (128 mg, 5.2 mmol) in dry Et_2O (2.0 mL). After completion of addition, the mixture was transferred to room temperature and stirred for an additional 1 h. Then the reaction flask was cooled to -25˚C before a solution of thionyl chloride (0.17 mL, 2.4 mmol) in 1 mL dry Et_2O was added gently. The reaction was continued for 4 h at r.t. before quenched with brine and DCM. The aqueous phase was extracted with DCM (20 mL × 2). The organic layer was combined, dried with anhydrous Na_2SO_4, concentrated in vacuum and purified with silica gel chromatography [elute from petroleum ether (PE) to mixed solvent PE/DCM = 12/1, v/v] to give compound **pf2** as a light brown solid (0.53 g, 56% yield).

^{19}F NMR (377 MHz, $CDCl_3$) δ -139.19 – -139.41 (m, 4F), -144.59 – -145.03 (m, 2F), -157.34 – -158.03 (m, 4F).

MS (EI): 382.0, 366.0, 347.0, 215.0, 199.0, 183.0, 155.0, 117.0.

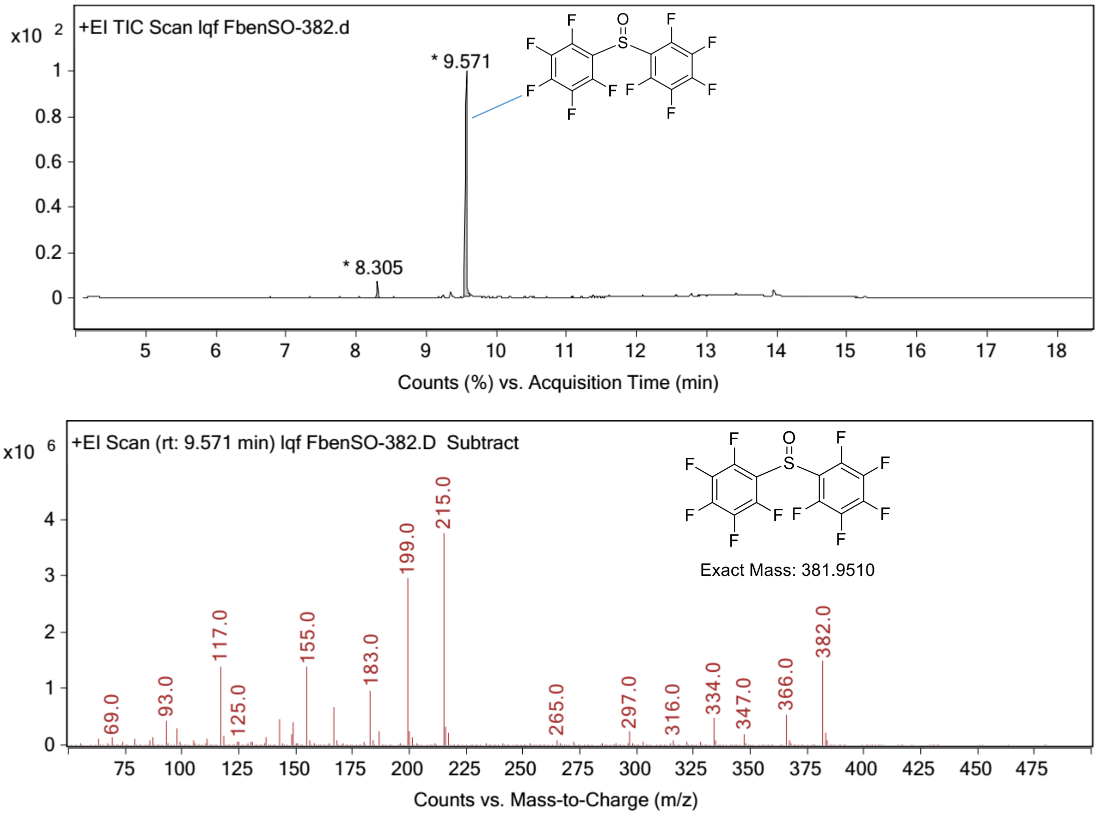

Supplementary Figure 85. GC-MS spectrum of compound **pf2**.

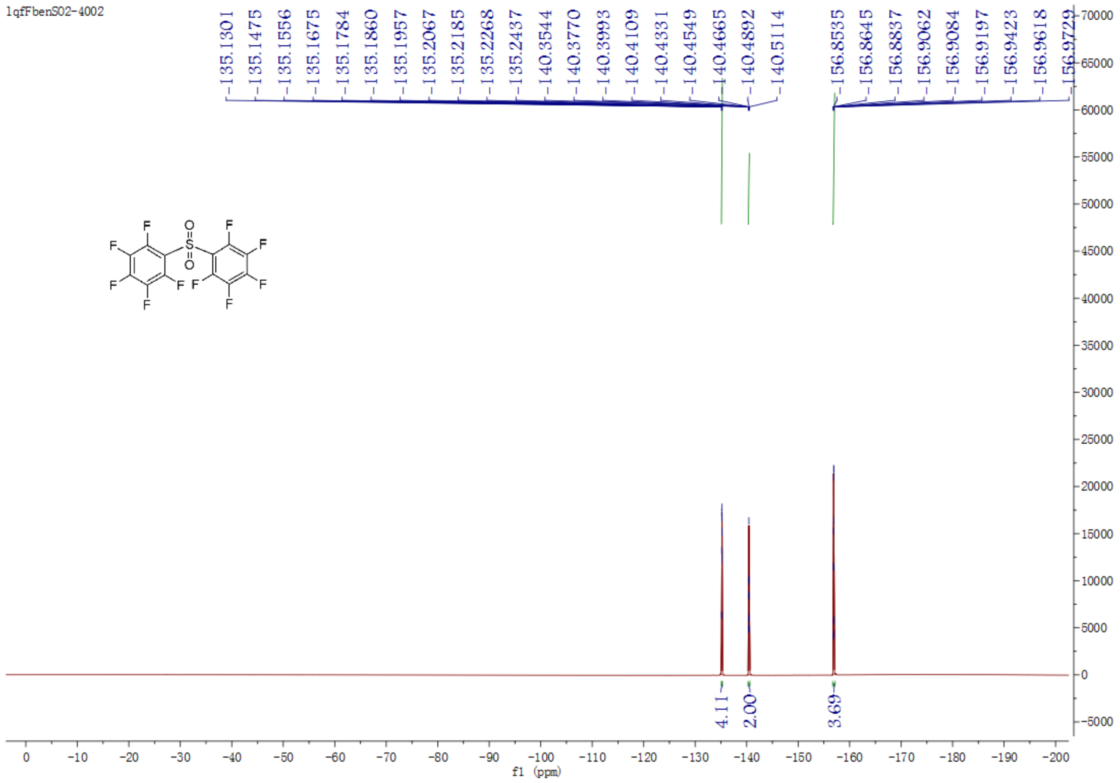

Supplementary Figure 86. ^{19}F NMR spectrum of compound **pf1**.

Synthesis of compound 6,6'-sulfonylbis(1,2,3,4,5-pentafluorobenzene) (pf1). Referring to the reported method,[3] a heavy wall pressure vessel (Synthware glass) was charged with compound **pf2** (38.2 mg, 0.10 mmol, 1.0 eq.) and 3-chlorobenzoic acid (69.0 mg, 0.40 mmol, 4.0 eq.). The mixture was suspended in 2 mL of dichloromethane and the tube was sealed with a Teflon cap. The reaction tube was placed in a preheated oil bath (110 °C) behind a blast shield and vigorously stirred for 24 h. At this time, the reaction vessel was cooled to room temperature and the solution was concentrated under reduced pressure. After concentration, the crude product was purified by silica gel column chromatography (PE/DCM = 15/2, v/v) to provide the title compound **pf1** as a white solid (31.0 mg, 78%).

^{19}F NMR (377 MHz, $CDCl_3$) δ -135.09 – -135.29 (m, 4F), -140.43 (m, 2F), -156.69 – -157.18 (m, 4F).

MS (EI): 398.0, 231.0, 215.0, 199.0, 183.0, 167.0, 155.0, 117.0.

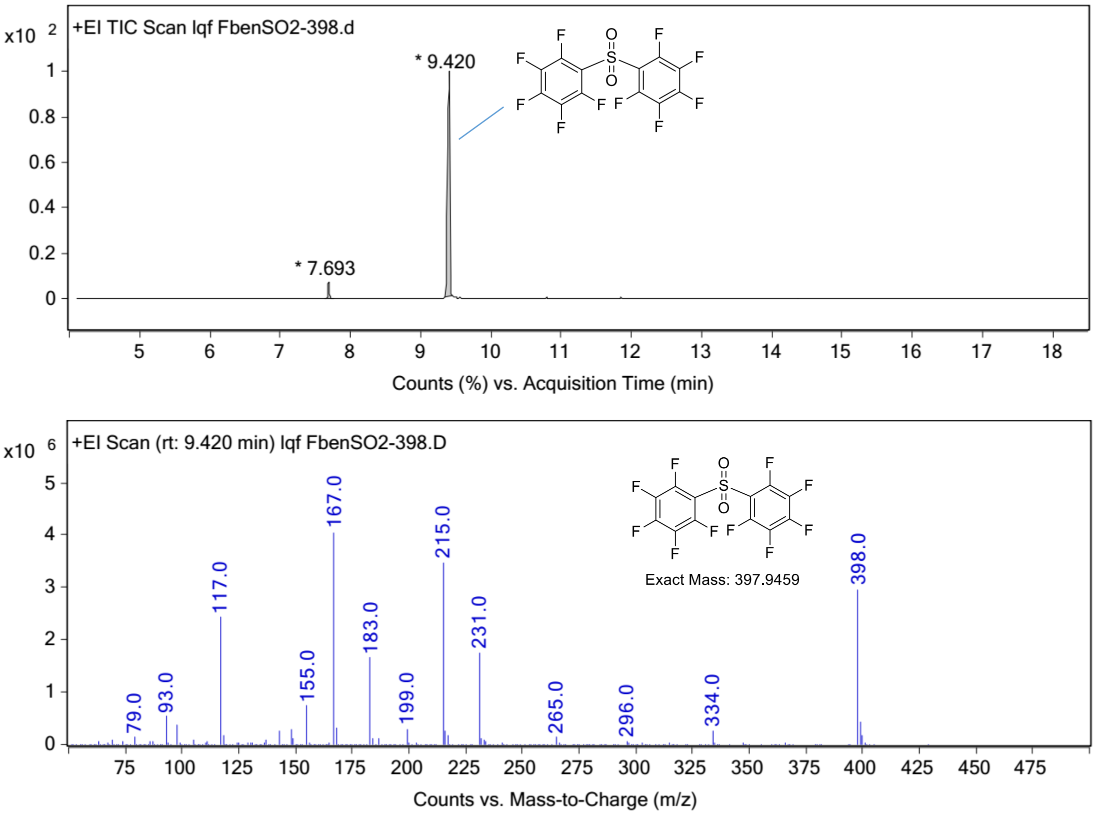

Supplementary Figure 87. GC-MS spectrum of compound **pf1**.

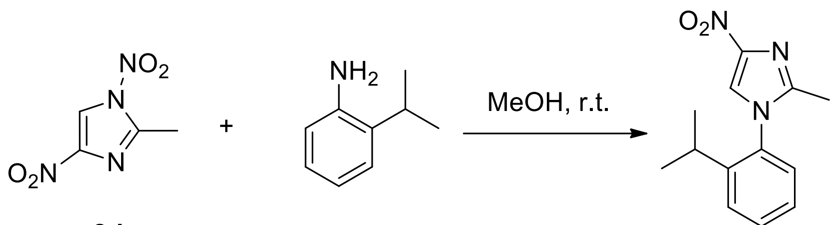

Supplementary Figure 88. Preparation of compound **1f**. Incubation of compound **2d** (34.4 mg, 0.2 mmol) with 2-isopropylaniline (54.0 mg, 0.4 mmol) in MeOH (6 mL) at r.t. for 4 h until TLC showed the completion of compound **2d**. The solution was concentrated in vacuum and the residue was subjected to silica gel column chromatography (PE/EA = 6/1, v/v) to give compound **1f** as white solid (35 mg, 72% yield).

^{1}H NMR (400 MHz, MeOD) δ 8.20 (s, 1H), 7.66 – 7.55 (m, 2H), 7.46 – 7.38 (m, 1H), 7.35 – 7.32 (m, 1H), 2.54 (m, *J* = 6.9 Hz, 1H), 2.19 (s, 3H), 1.22 (d, *J* = 6.9 Hz, 3H), 1.20 (d, *J* = 6.9 Hz, 3H).

^{13}C NMR (100 MHz, MeOD) δ 144.90, 144.63, 143.89, 131.75, 129.32, 125.74, 125.51, 125.49, 120.37, 26.37, 21.67, 20.35, 10.13.

HRMS (ESI) m/z calcd. for $C_{13}H_{15}N_3NaO_2$ $[M+Na]^+$ 268.1062, found 268.1058.

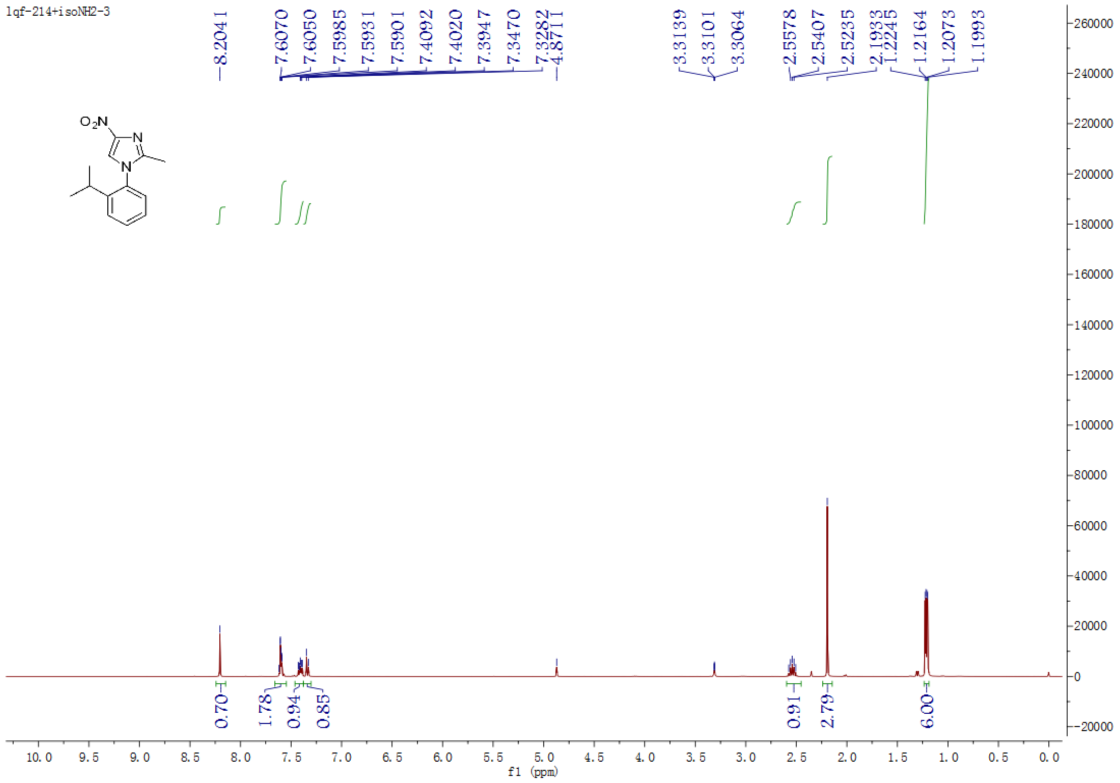

Supplementary Figure 89. ^{1}H NMR spectrum of compound **1f.**

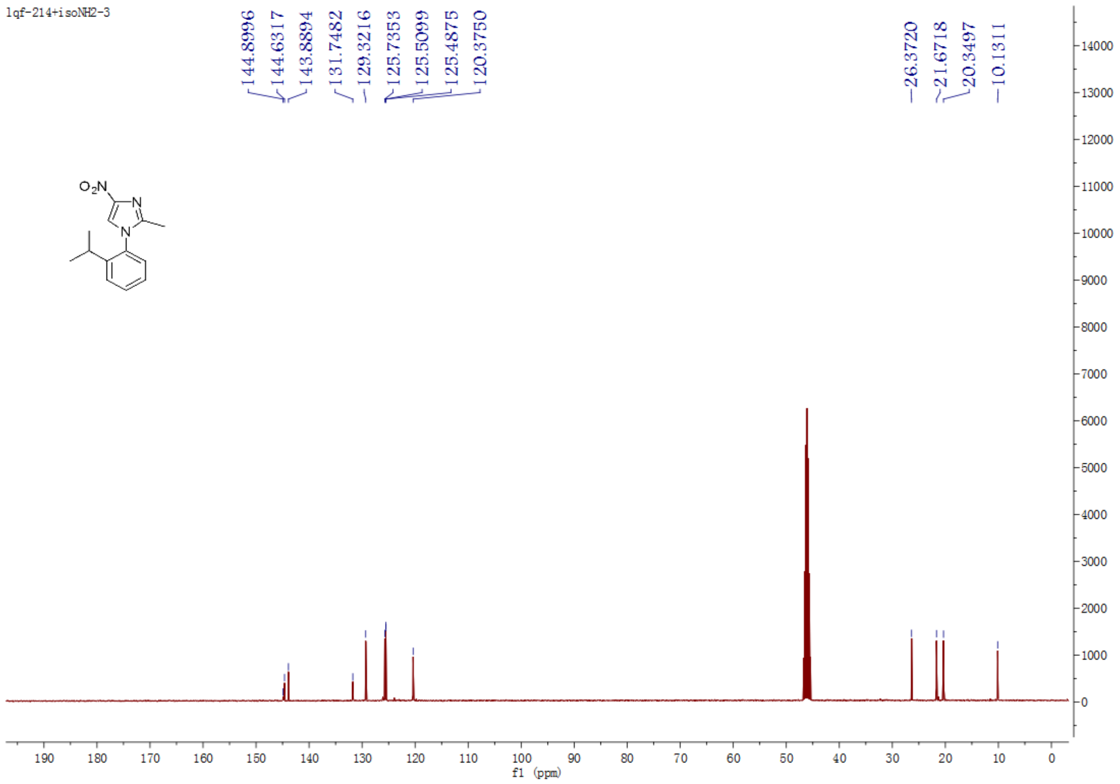

Supplementary Figure 90. ^{13}C NMR spectrum of compound **1f.**

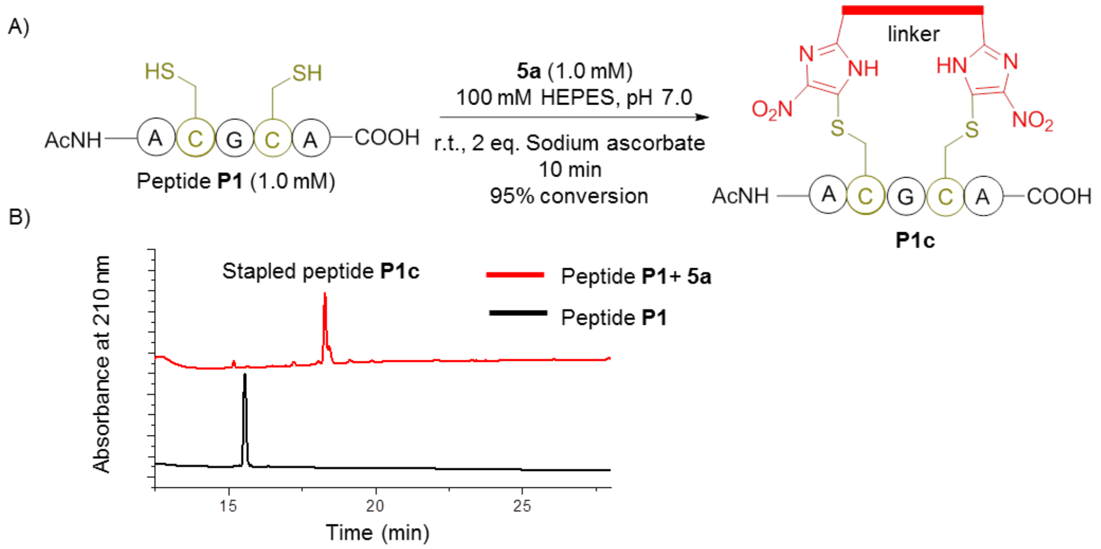

Supplementary Figure 91. Macrocyclization of peptide **P1** with bis(1,4-DNIm) **5a**. A) Reaction scheme; B) HPLC spectrum of the control experiment and the reaction mixture. Conversion is determined by using the peak AUC (Area Under Curve) of the linear peptide at 210 nm. MS data for **P1**: HRMS (ESI) m/z calcd. for $C_{16}H_{27}N_5NaO_7S_2$ $[M+Na]^+$ 488.1250, found 488.1240. **P1c**: HRMS (ESI) m/z calcd. for $C_{28}H_{36}N_{11}O_{15}S_2$ $[M+H]^+$ 830.1834, found 830.1830.

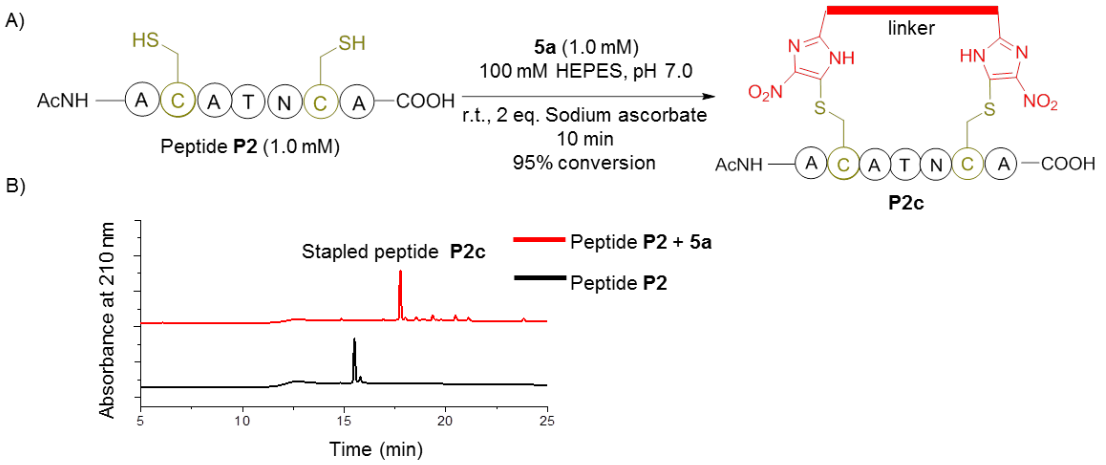

Supplementary Figure 92. Macrocyclization of peptide **P2** with bis(1,4-DNIm) **5a.** A) Reaction scheme; B) HPLC spectrum of the reaction mixture. Conversion is determined by using the peak AUC of the linear peptide at 210 nm. Analytical data for **P2**: HRMS (ESI) m/z calcd. for $C_{25}H_{42}N_8NaO_{11}S_2$ $[M+Na]^+$ 717.2312, found 717.2299. **P2c**: HRMS (ESI) m/z calcd. for $C_{37}H_{51}N_{14}O_{19}S_2$ $[M+H]^+$ 1059.2896, found 1059.2858.

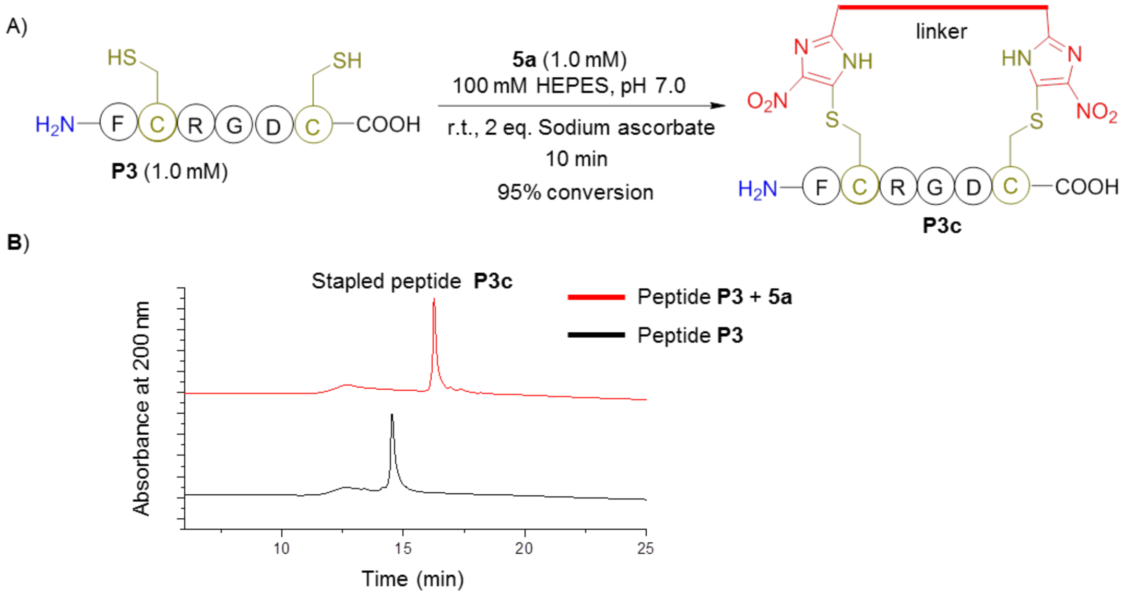

Supplementary Figure 93. Macrocyclization of peptide **P3** containing N-terminal free amino with bis(1,4-DNIm) **5a**. A) Reaction scheme; B) HPLC spectrum of the reaction. Conversion is determined by using the peak AUC of the linear peptide at 200 nm. Analytical data for **P3**: HRMS (ESI) m/z calcd for $C_{27}H_{42}N_9O_9S_2$ $[M+H]^+$ 700.2547,

found 700.2537. **P3c**: HRMS (ESI) m/z calcd for $C_{39}H_{50}N_{15}O_{17}S_2$ $[M+H]^+$ 1064.2951, found 1064.2949.

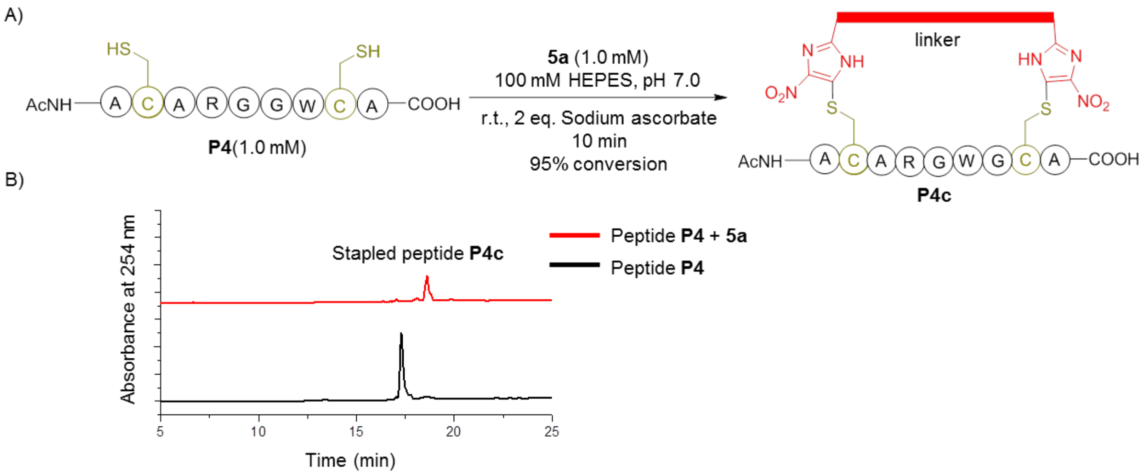

Supplementary Figure 94. Macrocyclization of peptide **P4** with bis(1,4-DNIm) **5a**. A) Reaction scheme; B) HPLC spectrum of the reaction mixture. Conversion is determined by using the peak AUC of the linear peptide at 254nm. Analytical data for **P4**: HRMS (ESI) m/z calcd. for $C_{38}H_{58}N_{13}O_{11}S_2$ $[M+H]^+$ 936.3820, found 936.3805. **P4c**: HRMS (ESI) m/z calcd. for $C_{50}H_{66}N_{19}O_{19}S_2$ $[M+H]^+$ 1300.4224, found 1300.4201.

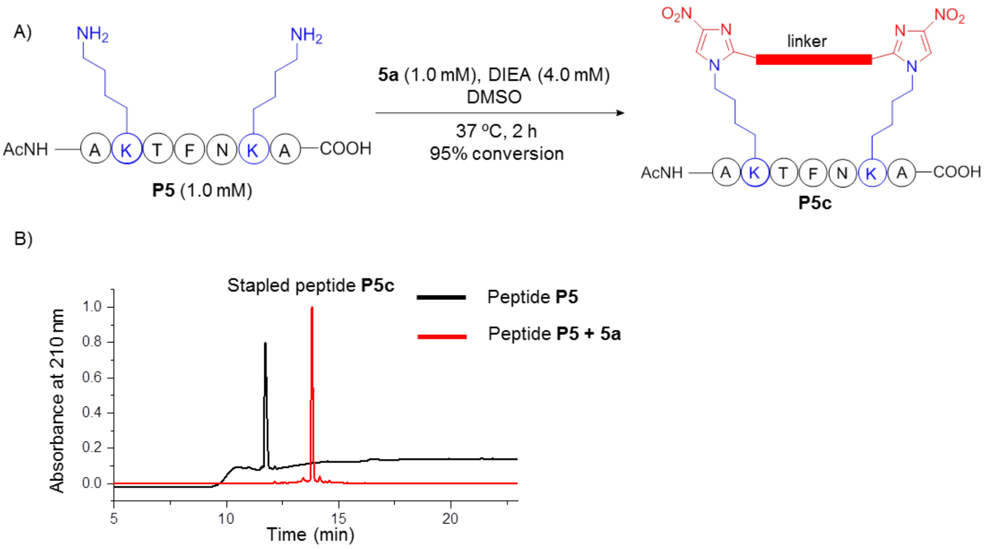

Supplementary Figure 95. Macrocyclization of peptide **P5** with bis(1,4-DNIm) **5a.** A) Reaction scheme; B) HPLC spectrum of the reaction mixture. Analytical data for **P5**: HRMS (ESI) m/z calcd. for $C_{37}H_{61}N_{10}O_{11}$ $[M+H]^+$ 821.4521, found 821.4519. **P5c**: HRMS (ESI) m/z calcd. for $C_{49}H_{65}N_{14}O_{19}$ $[M-H]^-$ 1153.4550, found 1153.4557.

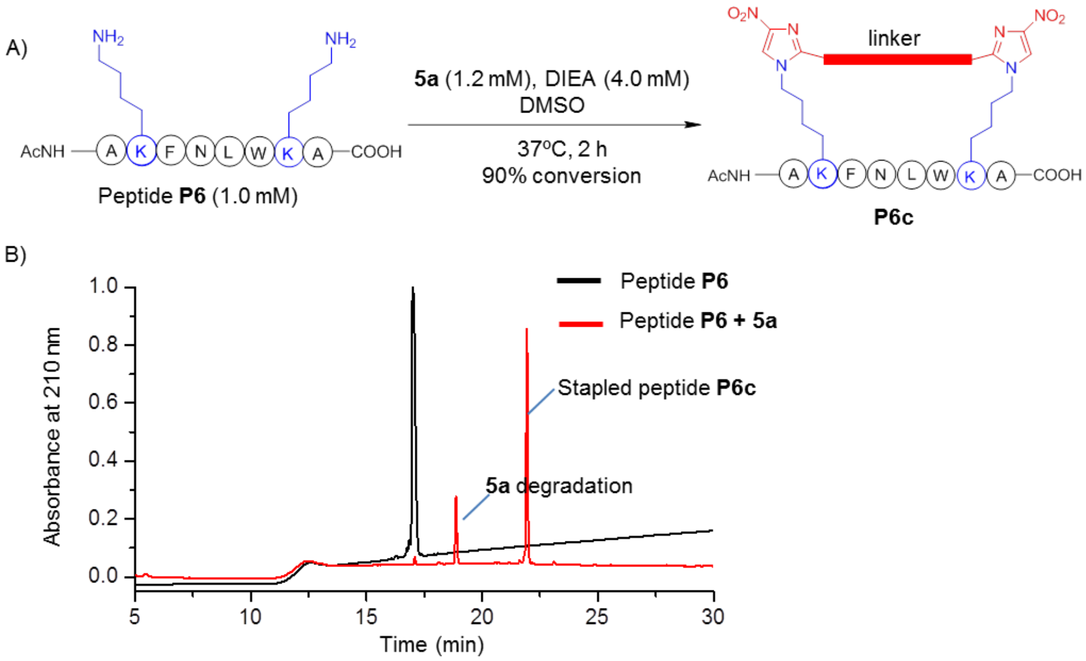

Supplementary Figure 96. Macrocyclization of peptide **P6** with bis(1,4-DNIm) **5a.** A) Reaction scheme; B) HPLC spectrum of the reaction mixture. Analytical data for **P6**: HRMS (ESI) m/z calcd. for $C_{50}H_{75}N_{12}O_{11}$ [M+H]+ 1019.5678, found 1019.5662. **P6c**: HRMS (ESI) m/z calcd. for $C_{62}H_{80}N_{16}NaO_{19}$ $[M+Na]^+$ 1375.5683, found 1375.5675.

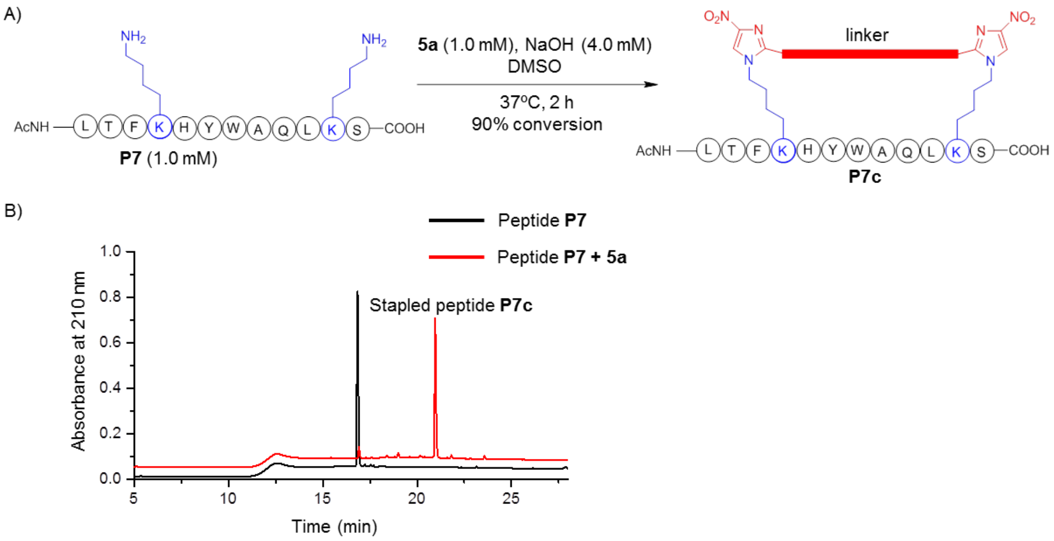

Supplementary Figure 97. Macrocyclization of peptide **P7** with bis(1,4-DNIm) **5a**. A) Reaction scheme; B) HPLC spectrum of the reaction mixture. Analytical data for **P7**: HRMS (ESI) m/z calcd. for $C_{76}H_{112}N_{18}O_{18}$ $[M+2H]^{2+}$ 782.4201, found 782.4195. **P7c**: HRMS (ESI) m/z calcd. for $C_{88}H_{117}N_{22}NaO_{26}$ $[M+H+Na]^{2+}$ 960.4203, found 960.4181.

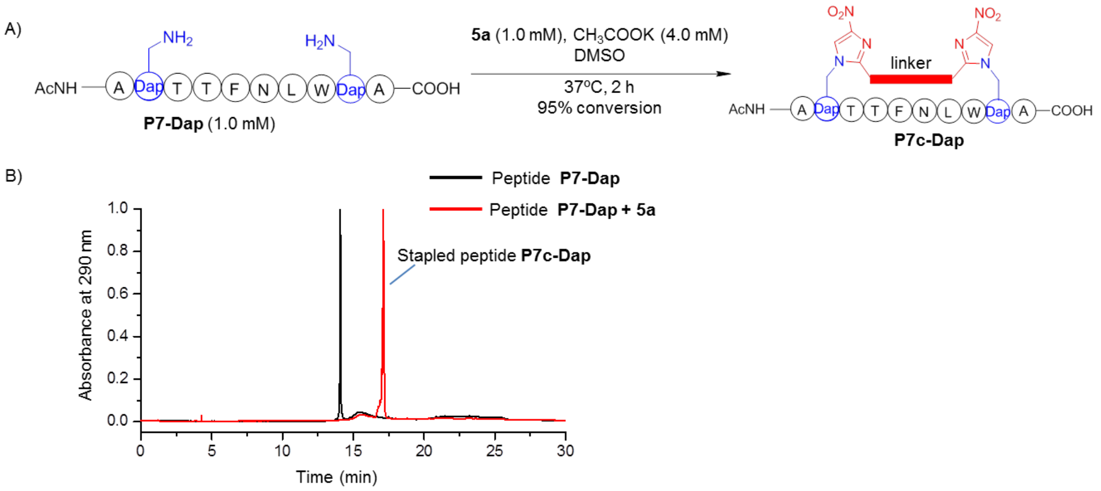

Supplementary Figure 98. Macrocyclization of peptide **P7-Dap** with bis(1,4-DNIm) **5a**. A) Reaction scheme; B) HPLC spectrum of the reaction mixture. Analytical data for **P7-Dap**: HRMS (ESI) m/z calcd. for $C_{52}H_{76}N_{14}NaO_{15}$ $[M+Na]^+$ 1159.5512, found 1159.5501. **P7c-Dap**: HRMS (ESI) m/z calcd. for $C_{64}H_{83}N_{18}O_{23}$ $[M+H]^+$ 1471.5878, found 1471.5871

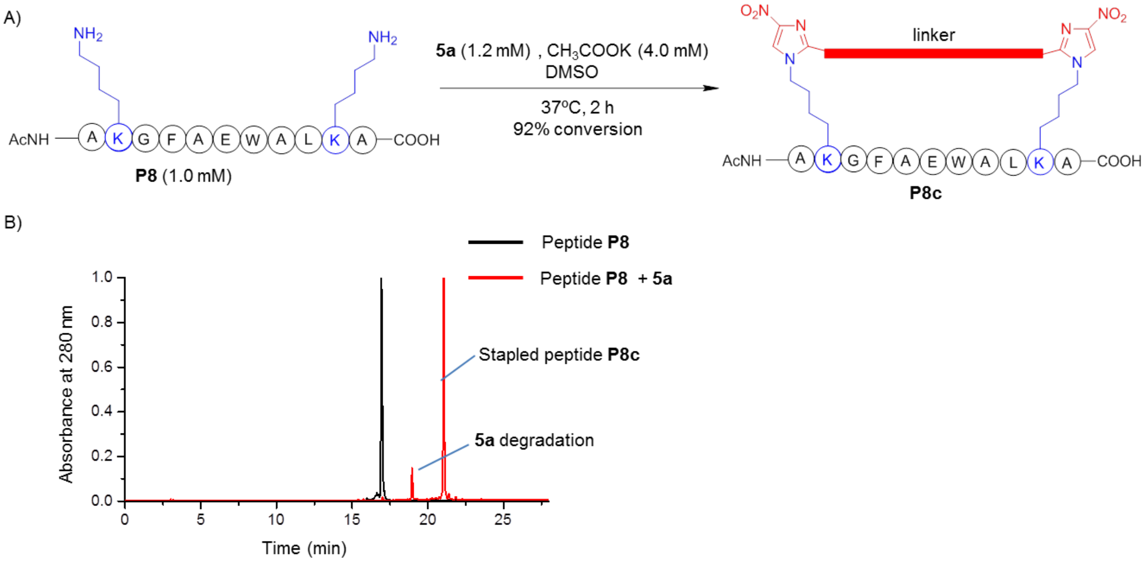

Supplementary Figure 99. Macrocyclization of peptide **P8** with bis(1,4-DNIm) **5a.** A) Reaction scheme; B) HPLC spectrum of the reaction mixture. Analytical data for **P8**: HRMS (ESI) m/z calcd. for $C_{62}H_{94}N_{15}O_{17}$ $[M+Na]^+$ 1320.6952, found 1320.6958. **P8c**: HRMS (ESI) m/z calcd. for $C_{74}H_{101}N_{19}O_{25}$ $[M+2H]^{2+}$ 827.8608, found 827.8600

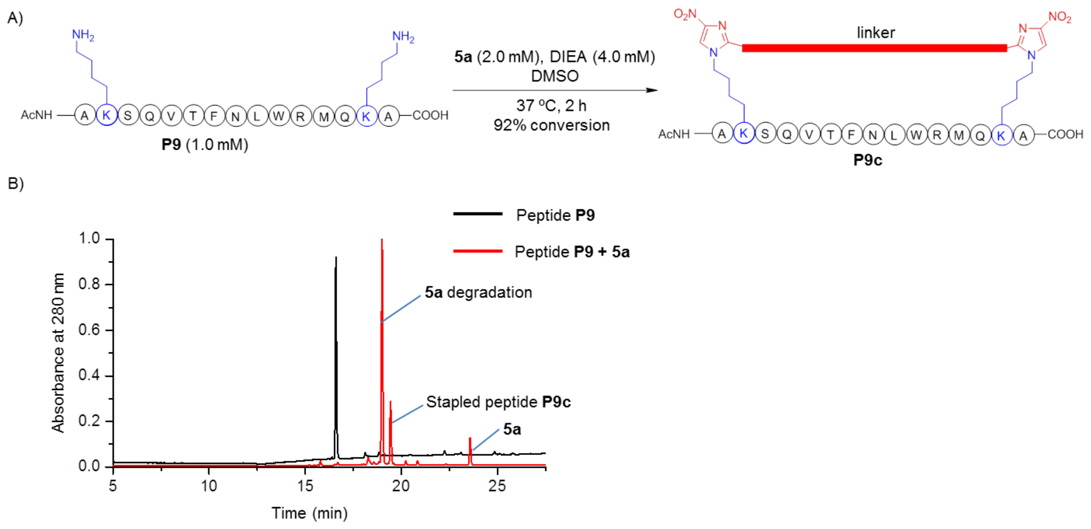

Supplementary Figure 100. Macrocyclization of peptide **P9** with bis(1,4-DNIm) **5a**. A) Reaction scheme; B) HPLC spectrum of the reaction mixture. Analytical data for **P9**: HRMS (ESI) m/z calcd. for $C_{83}H_{134}N_{24}O_{22}S$ $[M+2H]^{2+}$ 925.4913, found 925.4914. **P9c**: HRMS (ESI) m/z calcd. for $C_{95}H_{139}N_{28}NaO_{30}S$ $[M+H+Na]^{2+}$ 1103.4915, found 1103.4884.

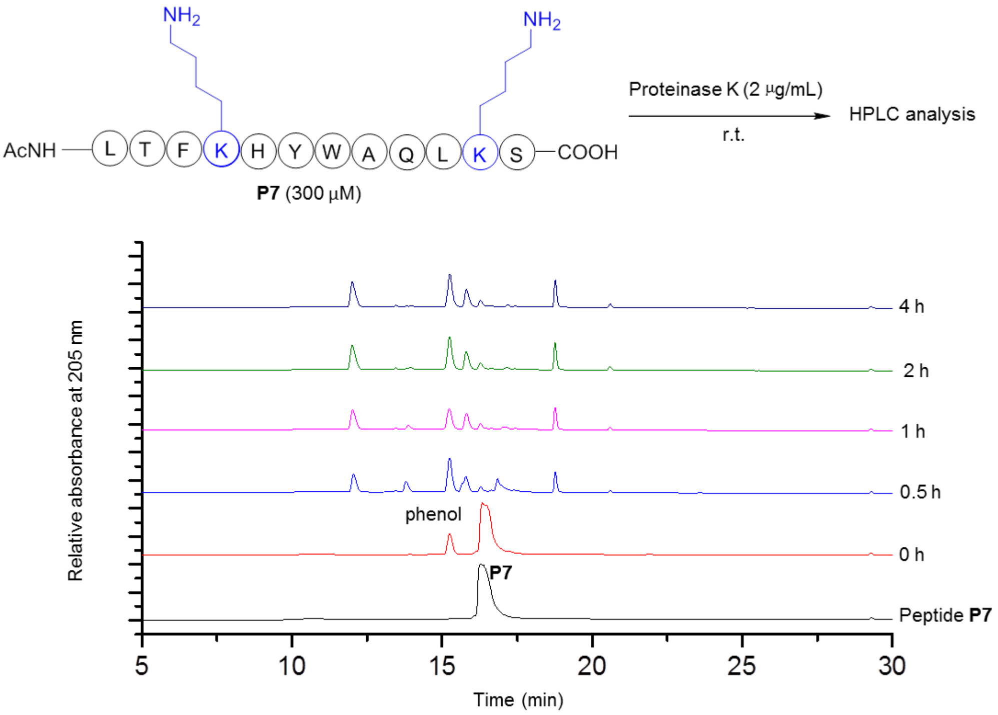

Supplementary Figure 101. HPLC spectrum of linear peptide **P7** under Proteinase K digestion. A 600 μL reaction mixture composed of 20 mM phosphate buffer pH 7.0, 300 μM peptide **P7**, and 2 μg/mL Proteinase K was incubated at room temperature for 4 hours. At time 0, 0.5 h, 1 h, 2 h, and 4 h, 100 μL of the crude reaction was quenched by addition of 1 μL 20% HCOOH before subjected to HPLC analysis. For quantification, phenol (final concentration 500 μM) was added as an internal standard.

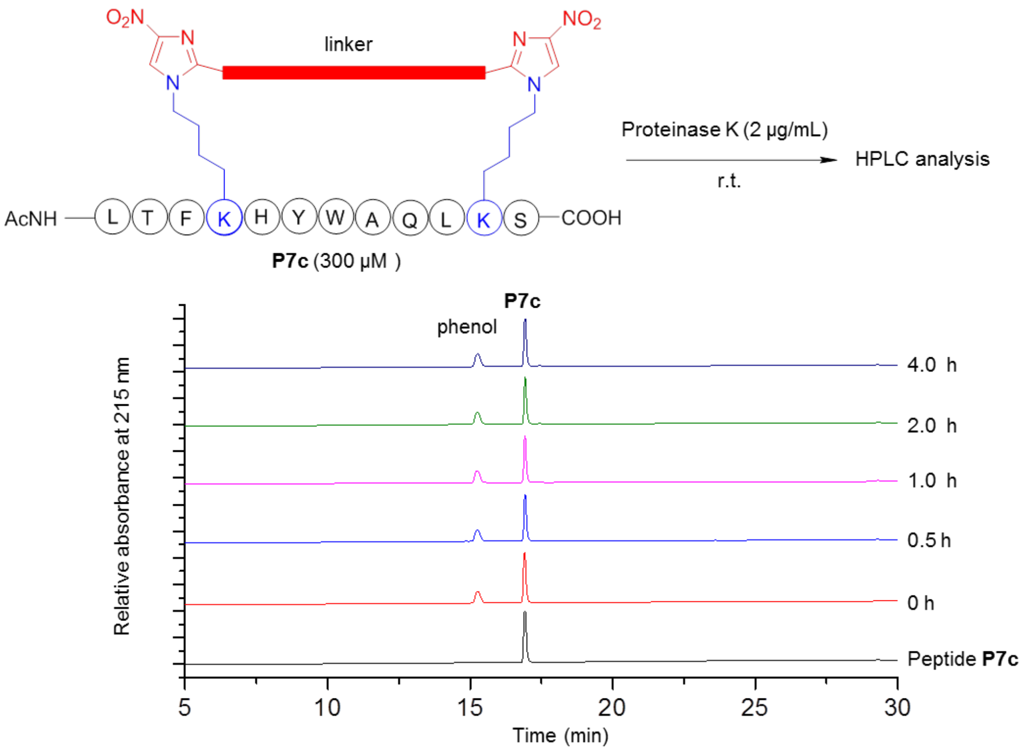

Supplementary Figure 102. HPLC spectrum of cyclic peptide **P7c** under Proteinase K digestion. A 600 µL reaction mixture composed of 20 mM phosphate buffer pH 7.0, 300 µM peptide **P7c**, and 2 µg/mL Proteinase K was incubated at room temperature for 4 hours. At time 0, 0.5 h, 1 h, 2 h, and 4 h, 100 µL of the crude reaction was quenched by addition of 1 µL 20% HCOOH before subjected to HPLC analysis. For quantification, phenol (final concentration 500 µM) was added as an internal standard.

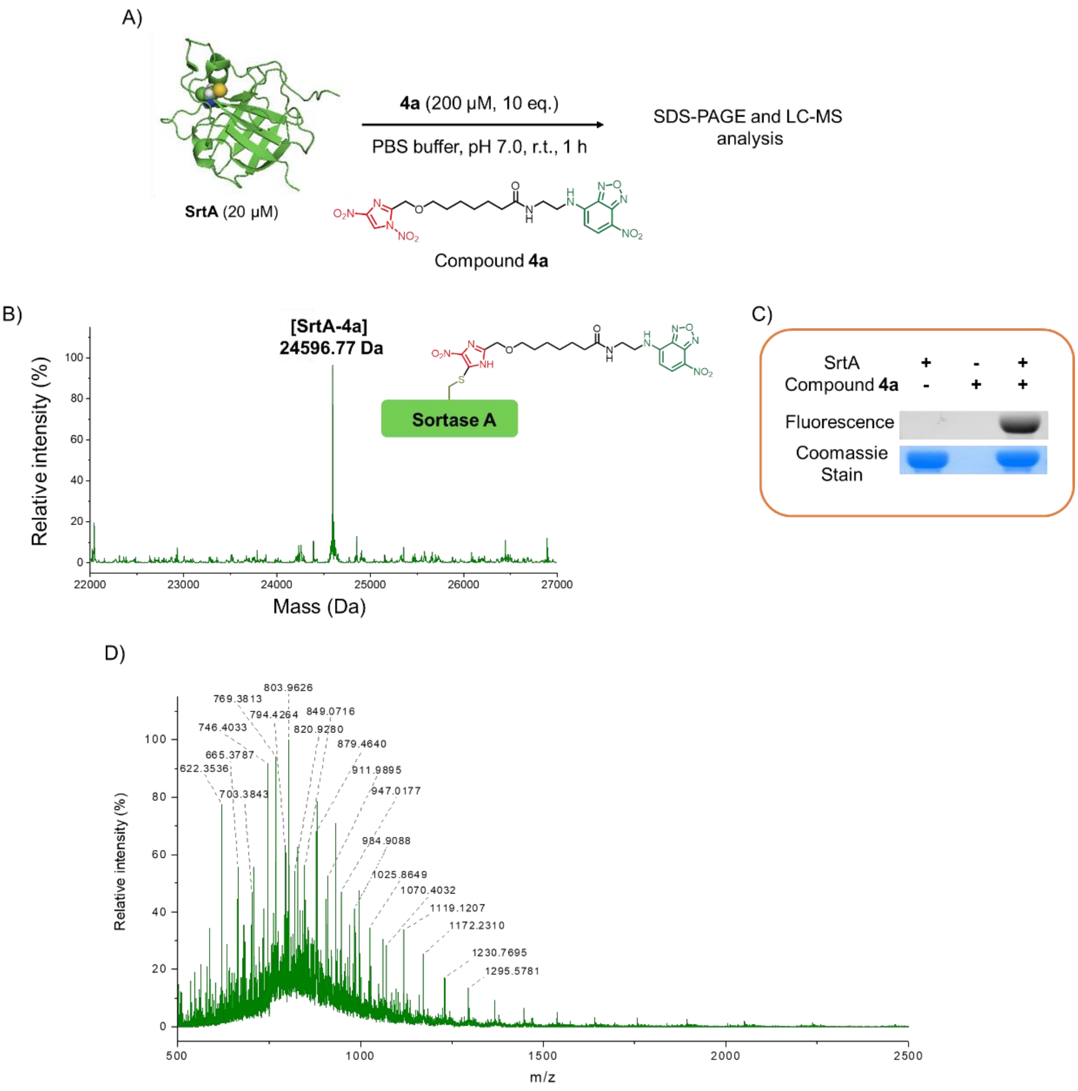

Supplementary Figure 103. SDS-PAGE and LC-MS analysis of SrtA modification by compound **4a**. A) Protein SrtA modified by **4a**. B) MS spectrum of modified SrtA by **4a**. Molecular mass (Average): SrtA calcd. 24136.03 Da; SrtA-**4a** calcd. 24596.44 Da (+460.41 Da), observed 24596.77 Da. C) SDS-PAGE analysis of the reaction. D) Combined ion series of the reaction of SrtA modified by compound **4a**.

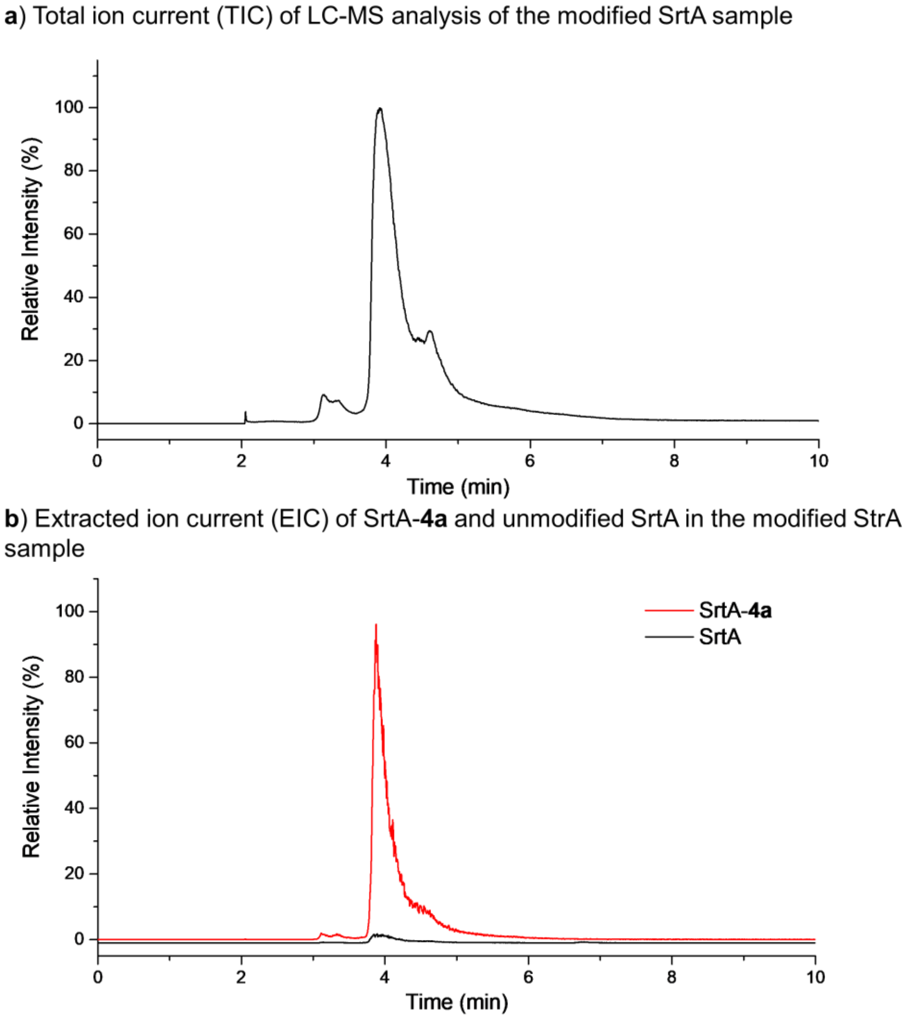

Supplementary Figure 104. LC-MS chromatogram of SrtA samples that are modified by compound **4a**. A) TIC trace of **4a**-modified SrtA sample. B) EIC trace of unmodified and modified SrtA proteins. $[SrtA+29H]^{29+}$ with a calculated mass of 833.2778 Da was selected as the monitor ion for unmodified SrtA; [SrtA-**4a**+29H]$^{29+}$ with a calculated mass of 849.1635 Da was selected as the monitor ion of modified SrtA.

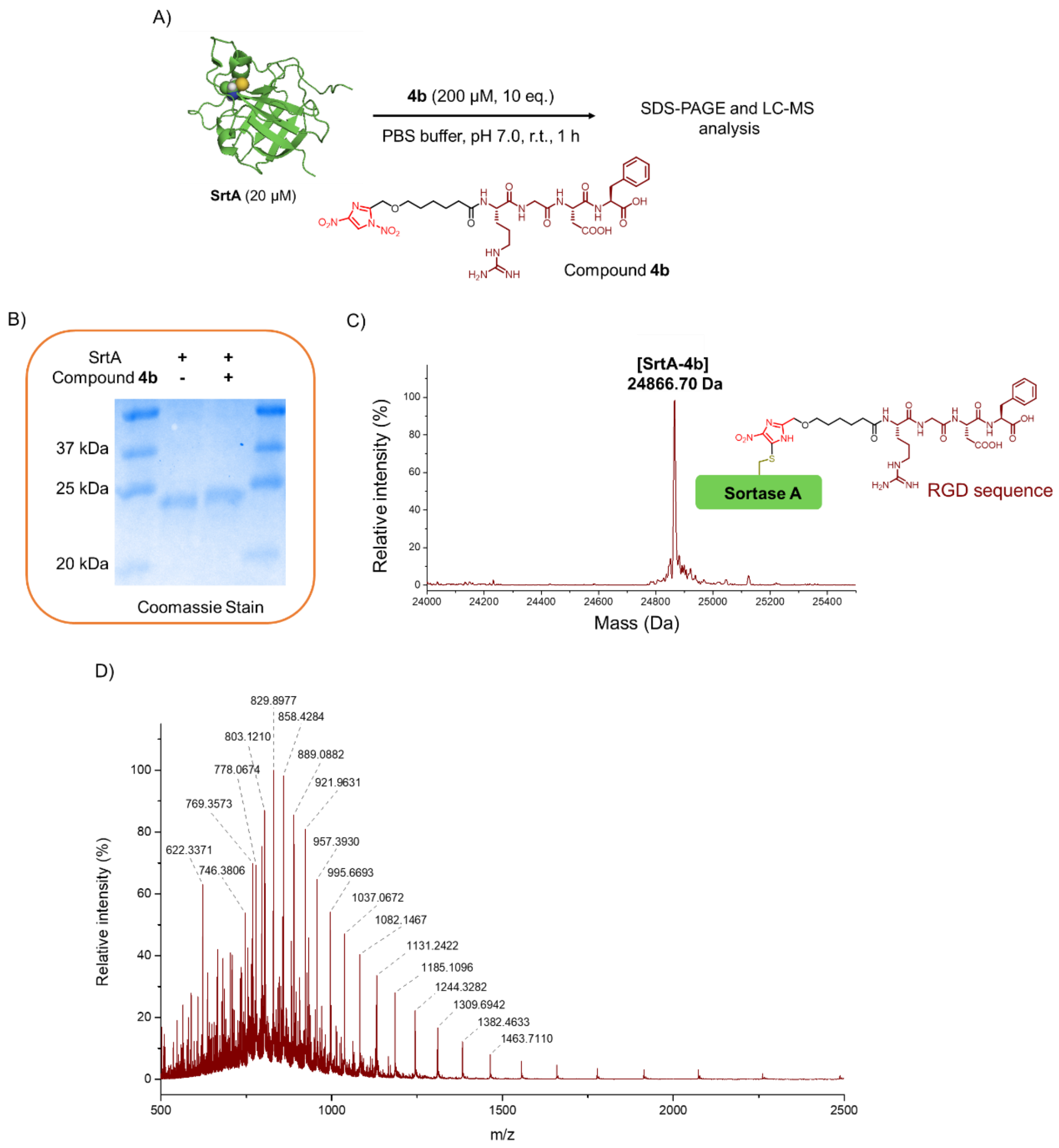

Supplementary Figure 105. SDS-PAGE and LC-MS analysis of SrtA modification by compound **4b**. A) Protein SrtA modified by **4b**; B) SDS-PAGE analysis of the reaction. C) LC-MS analysis of modified SrtA by **4b**. Molecular mass (Average): SrtA calcd. 24136.03 Da; SrtA-**4b** calcd. 24866.77 Da (+730.74 Da), observed 24866.70 Da. D) Combined ion series of the reaction of SrtA modified by compound **4b**.

a) Total ion current (TIC) of LC-MS analysis of the modified SrtA sample

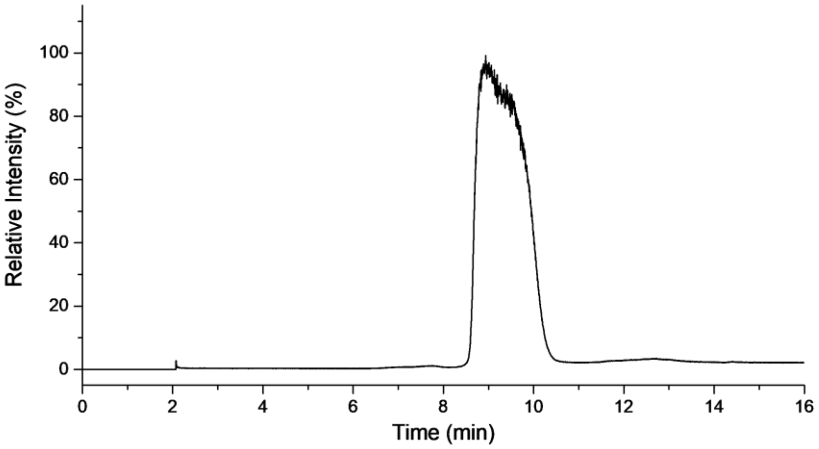

b) Extracted ion current (EIC) of SrtA-**4b** and unmodified SrtA in the modified StrA sample

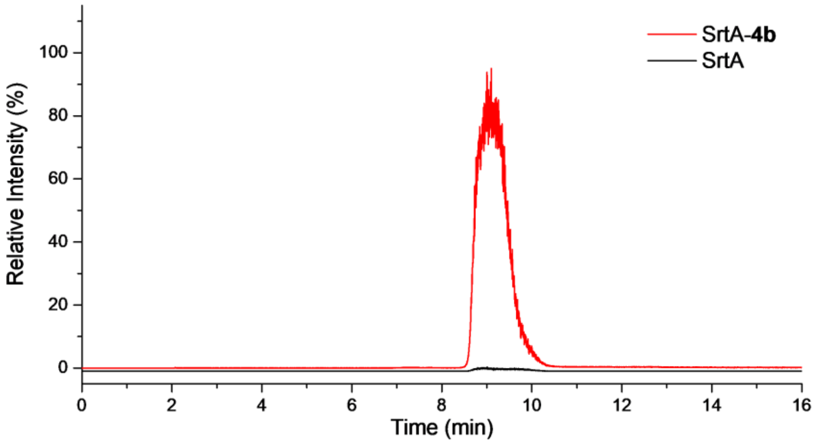

Supplementary Figure 106. LC-MS chromatogram of SrtA samples that are modified by compound **4b**. A) TIC trace of **4b**-modified SrtA sample. B) EIC trace of unmodified and modified SrtA proteins. $[\mathrm{SrtA+29H}]^{29+}$ with a calculated mass of 833.2778 Da was selected as the monitor ion for unmodified SrtA; [SrtA-**4b**+29H]$^{29+}$ with a calculated mass of 858.4632 Da was selected as the monitor ion of **4b**-modified SrtA.

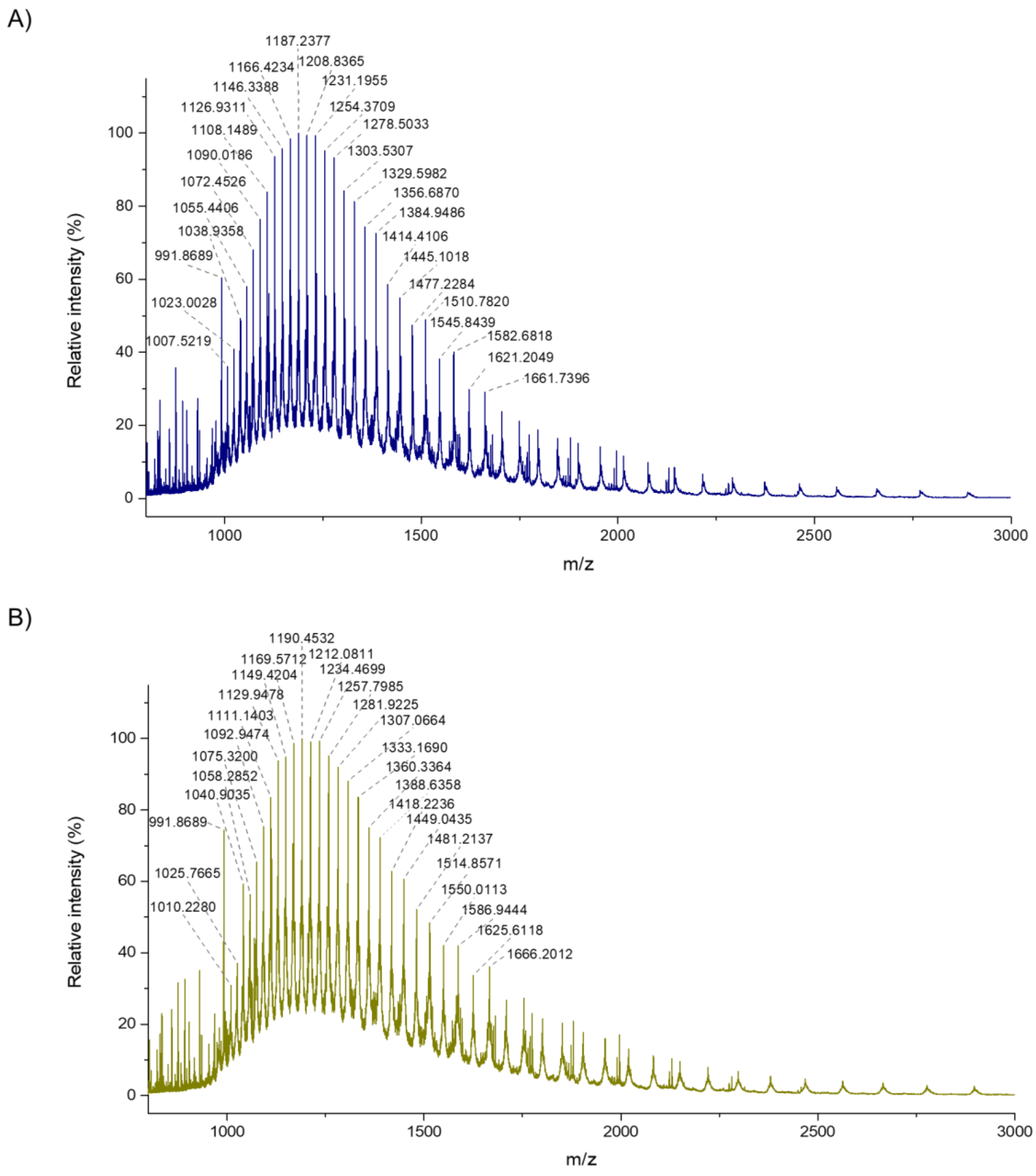

Supplementary Figure 107. MS spectrum of BSA and BSA-**4c** conjugate. A) Combined ion series of BSA. B) Combined ion series of BSA-**4c** conjugate.

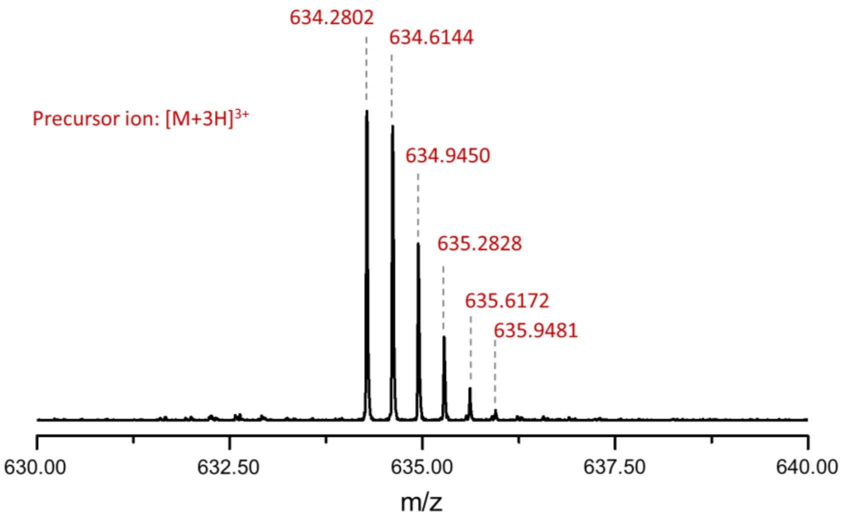

Precursor ion	Calculated m/z	Observed m/z	Relative deviation (ppm)
$[M+3H]^{3+}$	634.2810	634.2802	-1.2613

Supplementary Figure 108. Triply charged precursor ion of the cysteine modified peptide SQYLQQ<u>C</u>PFDEHVK from BSA. $[M+3H]^{3+}$ m/z calculated 634.2810, observed 634.2802. The underscore relates to the modified amino acid.

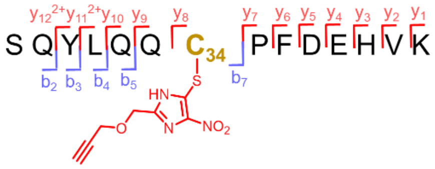

	Calculated m/z	Observed m/z	Relative deviation (ppm)
b2	216.0979	216.0951	-12.9571
b3	379.1613	379.1597	-4.2198
b4	492.2453	492.2438	-3.0473
b5	620.3039	620.3079	6.4485
b7	1030.4048	1030.4045	-0.2911
y1	147.1128	147.1127	-0.6798
y2	246.1812	246.1812	0.0000
y3	383.2402	383.2401	-0.2609
y4	512.2828	512.2838	1.9520
y5	627.3097	627.3040	-9.0864
y6	774.3781	774.3798	2.1953
y7	871.4309	871.4342	3.7869
y8	1153.4732	1153.4789	4.9416
y9	1281.5318	1281.5391	5.6963
y10	1409.5903	1409.5861	-2.9796
$y11^{2+}$	761.8408	761.8453	5.9067
$y12^{2+}$	843.3725	843.3745	2.3714

Supplementary Figure 109. MS/MS analysis of the cysteine modified peptide SQYLQQCPFDEHVK from BSA. The underscore relates to the modified amino acid. The b and y ions are listed in the table and marked in the spectrum. The amino acid sequence of this peptide is shown on top with cysteine residue highlighted in gold.

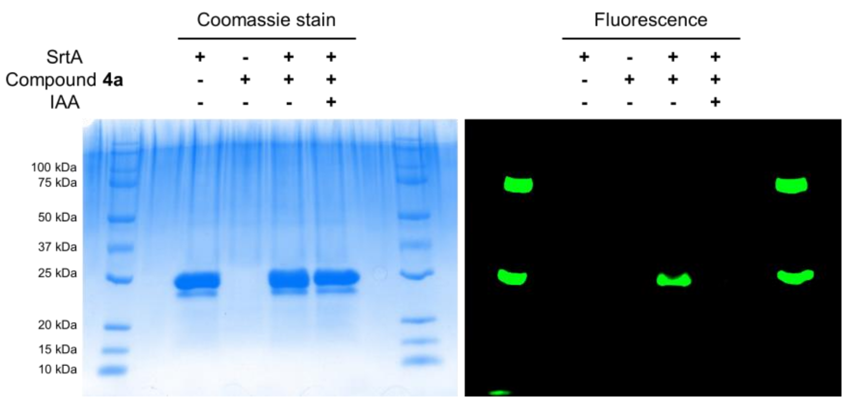

Supplementary Figure 110. SDS-PAGE analysis of the fluorescent labelling of SrtA or iodoacetamide-treated SrtA by compound **4a**.

Supplementary References

1 Bulusu, S. *et al.* Thermal Rearrangement of 1,4-Dinitroimidazole to 2,4-Dinitroimidazole: Characterization and Investigation of the Mechanism by Mass Spectrometry and Isotope Labeling. *J. Phys. Chem.* **99**, 5009-5015 (1995).

2 Bhaumik, K. & Akamanchi, K. G. 2,4-Dinitroimidazole: Microwave assisted synthesis and use in synthesis of 2,3-dihydro-6-nitroimidazo[2,1-b]oxazole analogues with antimycobacterial activity. *J. Heterocyclic Chem.* **41**, 51-55 (2004).

3 Lautrette, G., Touti, F., Lee, H. G., Dai, P. & Pentelute, B. L. Nitrogen Arylation for Macrocyclization of Unprotected Peptides. *J. Am. Chem. Soc.* **138**, 8340-8343 (2016).
